# Advances in Shoulder Pain Imaging: A Narrative Review of Current Practice and Emerging Trends

**DOI:** 10.2174/0115734056441053251112071648

**Published:** 2025-11-29

**Authors:** António Proença Caetano, André Barros, Eduardo Carpinteiro, Augusto Gaspar, Marina Ribeiro, Fernando Gonçalves, Pedro Soares Branco, Vasco Vogado Mascarenhas

**Affiliations:** 1 Interventional Radiology Unit, Unidade Local de Saúde – São José, Lisboa, Portugal; 2 Radiology Department, Hospital da Luz, Lisbon, Portugal; 3 Nova Medical School, Faculdade de Ciências Médicas, Universidade Nova de Lisboa, Lisboa, Portugal; 4 Department of Orthopedic, Hospital da Luz, Lisbon, Portugal; 5 Department of Radiology, Hospital da Luz, Lisbon, Portugal; 6 Department of Physical Rehabilitation, Unidade Local de Saúde – São José, Lisbon, Portugal Nova Medical School, Lisbon, Portugal

**Keywords:** Advanced imaging, Magnetic resonance imaging, Magnetic resonance arthrography, Shoulder, Rotator cuff, Quantitative imaging, Fat quantification, Deep learning, Artificial intelligence, T2 mapping, Cine-MRI, Dynamic-MRI

## Abstract

Shoulder pain is among the most frequent musculoskeletal complaints and remains a significant therapeutic challenge in clinical practice. A wide spectrum of conditions may contribute to this symptom, including rotator cuff tendinosis or tears, calcific tendinopathy, labral or capsuloligamentous injuries and degenerative changes of the glenohumeral joint.

Accurate diagnosis requires an integrated approach that combines clinical history, physical examination, and imaging. However, variability in examination technique and interpretation often limits the reliability of clinical assessment alone. Diagnostic imaging plays a crucial role in evaluating the shoulder joint and its surrounding soft-tissue structures. Magnetic resonance imaging has become the gold standard for shoulder evaluation due to its high resolution and superior soft-tissue contrast, allowing for a detailed assessment of tendons, muscles, cartilage, and bone marrow. Magnetic resonance arthrography further enhances sensitivity for labroligamentous and cartilage injuries, and remains essential in many clinical scenarios.

Recent technological advancements, such as radial imaging, kinematic or cine-MRI, 3D acquisition and reconstruction, dynamic contrast-enhanced sequences, ultrashort time-to-echo imaging, T2 mapping, and fat quantification, are expanding the diagnostic capabilities of MRI and promoting a shift from qualitative to quantitative evaluation of tissue integrity. Additionally, demand for faster imaging has driven the development of accelerated acquisition techniques that retain diagnostic image quality with shorter acquisition times.

Emerging artificial intelligence-driven tools are beginning to influence every stage of imaging, from protocol optimization to automated segmentation and the extraction of quantitative biomarkers. These innovations promise to improve diagnostic accuracy, streamline workflows, and usher in a new era of patient-specific care in shoulder pain imaging.

## 1 INTRODUCTION

The shoulder is a complex joint with a wide range of motion (ROM), making it susceptible to numerous injuries and conditions. Shoulder disorders are common in both young and older individuals, with the incidence and prevalence of pain in the population ranging from 7.7-62% and 0.67-55%, respec-tively [1]. The rotator cuff (RC) tendons are the primary dynamic stabilizers of the glenohumeral joint (GHJ), and rotator cuff tears (RCTs) are a frequent cause of shoulder pain, increa-sing in prevalence with age and affecting more than 50% of individuals over 80 years [2]. Importantly, RCTs are present in up to 70% of patients who seek medical care for shoulder pain [3].

Other causes of shoulder pain include RC tendinitis with or without calcific deposits, long head of the biceps (LHB) tendinosis, and bursae inflammation, which may be associated with conditions, such as shoulder impingement, instability, labral tears, joint inflammation and/or effusion (Table **1** and Fig. (**1**) [4-17].

Diagnosis of shoulder disorders is fundamental to guide treatment, and imaging has become a vital tool in assessing both osseous and soft tissue abnormalities. Imaging of the shoulder should follow a dedicated history and physical examination by an experienced clinician. Magnetic resonance imaging (MRI) has become the gold standard for musculo-skeletal evaluation due to its excellent spatial resolution and tissue contrast capabilities. However, it may not always correlate with clinical symptoms. Adequate integration of patient history, examination, and an expert analysis of imaging findings is necessary to avoid misdiagnosis and misinterpretation. Comprehensively, the structural and functional complexity underlying the GHJ and surrounding structures has prompted the development of new MRI techniques with higher diagnostic accuracy.

This review summarizes the state-of-the-art MRI techniques with direct clinical applications for assessing shoulder pain, examining their strengths and limitations, and current challenges for clinical implementation (Table **2**) [18-44]. The literature included in this review was gathered through a structured search and selection process. A comprehensive search of the PubMed database was performed, focusing on studies published in English up to December 2024. Keywords and MeSH terms included shoulder pain, rotator cuff, MRI, advanced imaging, quantitative MRI, ultrashort echo time, and magnetic resonance spectroscopy, among others. Reference lists of relevant articles and reviews were also screened to identify additional studies. Both original research and review papers describing the clinical application or validation of advanced MRI techniques in shoulder pathology were considered for inclusion. The evidence was synthesized qualitatively, emphasizing clinical relevance, technical principles, strengths, limitations, and challenges for implementation in daily practice. Tables **S1** [4, 18, 22, 33, 41-71], **S2** [72-128], **S3** [19-21, 23-32, 34-40, 114, 129-183] and **S4** [25, 184-222] provide a summary of the recent studies and reviews included. The findings of this study aim to guide radiologists and orthopedic surgeons through the most promising innovations, organized according to imaging technique and clinical relevance.

## 2 FROM DIAGNOSIS TO TREATMENT

**Fig. (2):**
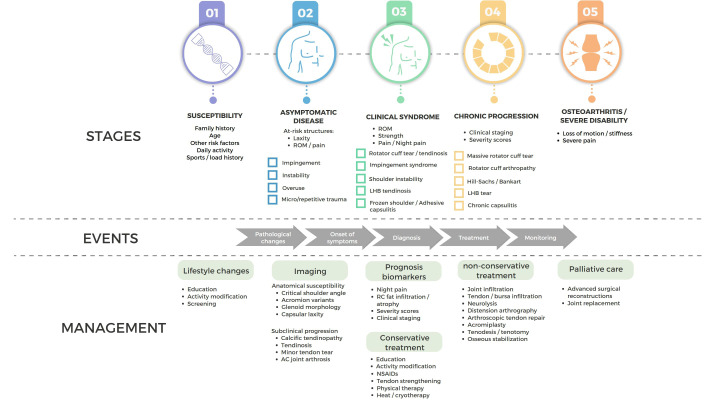
Natural history and management of major causes of shoulder pain.

An accurate diagnosis of the underlying musculoskeletal conditions causing pain and disability is necessary to guide treatment and prevent irreversible damage (Fig. **2**). Knowledge of the bony and soft tissue anatomic features, shoulder dynamics, and epidemiological risk factors that increase disease susceptibility (such as age, sex, daily/sports activities, laxity, diabetes mellitus, and hypercholesterolemia) is fundamental to performing a patient-centered and rational analysis. The initial evaluation of a patient with shoulder pain includes a detailed history taking and physical examination. When considering the range of differential diagnoses, clinical signs and physical tests, albeit useful and aimed at specific pathologies, often overlap and have shown limited specificity and reliability [223-227].

Impingement, a dynamic disturbance at the shoulder occurring during arm movement, may be classified into four types according to the site of soft-tissue entrapment: subacromial or external, subcoracoid, posterosuperior or anterosuperior inner impingement [4, 65]. Subacromial impingement (SAI) is the most common type, and it is thought to result from external compression by surrounding structures and/or intrinsic factors such as tendon degeneration. Several predisposing structural changes detectable on MRI include variations in acromion configuration, acromioclavicular joint abnormalities, and coracoacromial ligament (CAL) changes [65]. Intrinsic factors include damage to the RC tendons, particularly the supraspinatus (SSP), which increases tensile overload and promotes superior humeral head migration, perpetuating SAI. Some studies, however, have not supported the SAI theory and failed to find an association between anatomical characteristics and RCT [228-231]. Subcoracoid impingement is rare and refers to compression of the subscapularis (SSC) tendon between the humeral head and coracoid.

RC tendons may sustain microtrauma caused by impingement, acute traumatic lesions or repetitive overload, leading to degeneration (tendinosis), calcific deposit formation, and tears. The LHB tendon may similarly suffer degeneration and spontaneous rupture, and be a cause of significant disability. The subacromial bursa, a small fluid-filled sac that reduces tendon attrition and wear, may also become inflamed due to repetitive microtrauma or migration of tendon calcifications, leading to acute bursitis [232].

Shoulder instability may be atraumatic (multidirectional instability) or secondary to a traumatic event resulting in irreversible injury of the static and dynamic stabilizers of the GHJ [233]. Increased capsular or ligamentous laxity, as well as repetitive microtrauma from overuse, may lead to morphological changes that predispose individuals to instability and dislocation events.

Adhesive capsulitis (AC), also known as frozen shoulder, is an idiopathic condition that courses with shoulder rigidity due to synovial inflammation, capsular thickening and fibrosis, causing gradual loss of active and passive GHJ motion. Secondary capsulitis may be associated with previous trauma, calcific tendinitis or acute aggravation. Although generally regarded as a self-limiting disease, many patients progress to chronic disability and pain if left untreated.

### 2.1 Imaging Techniques

#### 2.1.1 Conventional Radiography

The shoulder is the second most frequently imaged joint after the knee. Conventional Radiography (CXR) plays a vital role in detecting structural changes in the shoulder joints and supporting soft tissues, particularly in emergency settings, to identify fractures and dislocations. Shoulder anteroposterior and lateral (scapular “Y”) radiographs are also routinely performed during early evaluation of non-traumatic shoulder pain and are recommended as the first imaging modality [234, 235]. Radiographs may reveal direct and indirect signs of injury, such as joint space narrowing in GHJ osteoarthritis or a reduced acromiohumeral distance (AHD) in RC pathology. However, they may otherwise appear normal in conditions like LHB tendinosis and AC.

#### 2.1.2 Computed Tomography

Computed tomography (CT) is often used in complex shoulder fractures for surgical planning. However, it also plays a role in elective evaluations for bone characterization, such as in planning shoulder arthroplasty and quantifying structural damage in recurrent shoulder instability [236-238]. CT is further indicated for assessment of post-operative complications and inflammatory or septic arthritis. It is the most sensitive modality for detecting soft-tissue calcifications, and indirect signs of tendon injury may be apparent. Still, its low contrast resolution for soft tissue makes it less favorable for detailed tendon evaluation.

#### 2.1.3 Ultrasound

Ultrasound offers multiple advantages over other imaging modalities, as it is radiation-free, widely available, and cost-effective, and is performed in real-time, with excellent soft-tissue contrast resolution for musculoskeletal evaluation [123, 239, 240]. Modern high-frequency probes reveal remarkable detail, allowing for the depiction of the fibrillar components of ligaments, tendons, and muscles. Functional modalities, such as elastography, are paving the way for a further understanding of tissue quality beyond purely structural changes.

The accuracy of ultrasound for RCT detection has been extensively studied and compared to MRI; some studies have reported equivalent diagnostic performance [241, 242] and strong agreement between the two modalities [239].

Minimally invasive procedures can also be easily performed with great precision under ultrasound guidance and have shown better results in terms of accuracy and clinical efficacy compared to palpation-guided techniques [243, 244].

#### 2.1.4 Magnetic Resonance Imaging and Arthrography

MRI is an indispensable tool in musculoskeletal imaging and remains the most valuable modality for diagnosing shoulder lesions [240], due to its multiplanar capabilities and high soft-tissue contrast resolution, which ultimately demonstrate high sensitivity and specificity for injury detection. MRI is the gold standard technique for assessing soft-tissue structures around the shoulder, including the RC tendons, LHB tendon, labrum, capsule, and ligaments.

Imaging at 1.5 Tesla (T) is generally sufficient for gross assessment, but 3.0 T systems provide higher spatial resolution and signal-to-noise ratio (SNR), allowing for the detailed visualization of small structures with clinical significance [47] Fig. (**3A-G**).

MRI is indicated in acute settings where there is suspicion for RCT, dislocation, contusion or occult fractures. Chronic pain or dysfunction unresponsive to conservative treatment also warrants MRI evaluation, which may detect labral lesions, tendinosis, bursitis or osteoarthritis.

The use of diluted intra-articular gadolinium-based contrast agents (or saline alone) in conjunction with refined imaging protocols, such as magnetic resonance arthrography (MRA), provides improved delineation of cartilage, labrum, and capsular structures, resulting in enhanced diagnostic accuracy. This technique is safe and has been widely used in specialized musculoskeletal centers.

### 2.2 Treatment

First-line treatment for chronic shoulder pain often includes medication (acetaminophen, non-steroidal anti-inflammatory drugs, muscle relaxants), activity modification, and rehabilitation exercises. When conservative treatment fails, a wide range of minimally invasive and surgical procedures are available to achieve clinical improvement.

In AC, the primary goals are pain relief and restoration of mobility. Anti-inflammatory drugs and physical therapy are the mainstays of treatment, but intra-articular corticosteroid injections may be employed to reduce capsular inflammation and promote ROM rehabilitation. If these measures are insufficient, surgical manipulation under anesthesia or percutaneous joint hydrodistension with saline has been shown to yield favorable outcomes [30].

Physical rehabilitation is employed in shoulder instability to strengthen the dynamic GHJ stabilizers (RC and other scapular muscles). For recurrent instability, capsular reconstruction, fixation, bone transfer or grafting may be performed using arthroscopic or open techniques.

The rate of definitive improvement within 1 year of conservative RCT treatment ranges from 33% to 92% [31]. Local corticosteroid injections may relieve pain, but their benefits remain controversial [32], as they improve symptoms but do not promote tendon healing or repair. Autologous blood products, such as platelet-rich plasma, have been explored in RCTs, LHB tendinosis, GHJ osteoarthritis and subacromial impingement, with mixed results [33].

Arthroscopic rotator cuff tear repair (ARCR) is considered the standard intervention for patients unresponsive to conservative treatments, but is associated with a high re-tear rate of 7,2-94% [34]. Surgical treatment strategies vary depending on the underlying pathology and potential causes of impingement [35], and may include tendon suture (RCT), surgical tendon or ligament release (*e.g*., LHB tendon, coracoacromial ligament), osteoplasty, and decompression (*e.g*., acromioplasty, spur debridement), among others. Advanced GHJ osteoarthritis and RC arthropathy may be managed with shoulder arthroplasty, muscle/tendon transfer or combined techniques.

## 3 ANATOMY AND IMAGING

### 3.1 Osseous Morphology

Bone morphology is a critical component of the evaluation in patients with suspected SAI. Several measurements have been proposed in the literature to assess humeral head and scapular morphology: the acromiohumeral distance, critical shoulder angle (CSA), acromial index (AI), lateral angle and lateral slope of the acromion, acromial slope and tilt, acromion-greater tuberosity impingement index, acromial angulation, acromial shape and glenoid version angle [51, 83, 104]. These parameters can be measured both on and in commonly performed anteroposterior and lateral radiographs and/or MRI [104, 105]. However, care should be taken when evaluating AHD as it appears significantly smaller on MRI compared to AP radiographs and should not be utilized to assess GHJ centering and subacromial space width [88]. In this regard, a volumetric MRI-based assessment of the subacromial space has been described [96].

RCT is associated with high AI, CSA, acromion-greater tuberosity impingement index, acromial angulation, glenoid retroversion, and low AHD and lateral angle [94, 104]. Acromial infero-lateral tilt may impact SAI [85], and a down-slope acromion has been observed more frequently in patients with shoulder pain [102]. The CSA, in particular, is a recently described parameter positively associated with degenerative shoulder pathologies (RCT and osteoarthritis) [245]. It is defined as the angle between the glenoid axis and the line connecting the inferior edge of the glenoid and the lateral edge of the acromion; anormal CSA ranges from 30-35º (<30º is associated with osteoarthritis, and >35º is associated with RC tears) [54, 95, 246] [230].

The association between acromial type as described by Bigliani *et al*. [247] and RCTs has been challenged in recent studies [248-250]. Several alternative measurements have been proposed to better assess the relationship between acromial morphology and RCTs, including acromial height, AI, AHD, lateral angle of the acromion or CSA. In agreement, a study conducted by Ma *et al*. showed that the combined application of humeral and scapular morphological parameters, namely the CSA, AI, greater tuberosity angle, and double-circle radius ratio, accurately diagnosed and predicted the risk of developing RCT [74]. A recent meta-analysis concluded that degenerative RCT is significantly associated with AI, CSA and the lateral angle of the acromion [81]. Importantly, high acromial slope and low AHD have been linked to re-tear following RCT repair [98]. Shoulder function and pain are best predicted by the subdeltoid bursa width in the coronal MRI plane and the presence of a “halo-sign” around the LHB [99].

Several studies have reported associations between coracoid morphology, coracohumeral parameters, coracohumeral ligament, lesser tuberosity morphology, and cysts with subcoracoid impingement or SSC tears [79, 84, 92, 93, 100, 107, 251-254], while others have found no such correlation [80, 97, 103]. Anecdotal reports have devised new parameters to evaluate shoulder impingement, with interesting results [80, 86, 108].

Evaluation of recurrent anterior shoulder instability includes imaging assessment of the proximal humeral head for structural changes, such as a Hill-Sachs lesion (HSL), and of the glenoid for surface bone loss (bony Bankart), both of which predispose to recurrent instability. Notably, the native glenoid morphology may also predispose males to shoulder instability, but not females [118].

Quantification of bone loss following trauma is a key factor in determining the optimal management of chronic anterior GHJ instability. CXR has low inter-observer reliability and lacks the accuracy required for pre-operative planning [53]. 3D CT is more accurate than 2D imaging for measuring the depth and width of HSL and remains the gold standard. Still, some studies have found no significant differences between 3D CT and 3D MRI measurements.

MRI has demonstrated moderate to good performance in glenoid measurements [117, 255], although it is less accurate than 3D CT [8, 256, 257]. Recent advancements in 3D MRI techniques have led to comparable accuracy in quantifying glenoid bone loss [112, 114] and identifying HSL [111]. Stillwater *et al*. [111] conducted a prospective study comparing 3D MRI and 3D CT in every patient enrolled, measuring glenoid height, glenoid width, humeral head height, humeral head width, HSL size, percentage of humeral head loss, size of glenoid bone loss, and percentage of glenoid bone loss. It was concluded that 3D MRI and 3D CT measurements are equivalent.

The glenoid track concept was introduced in 2007 by Yamamoto *et al*. [258] as a quantitative assessment of the degree of humeral head bony defect and glenoid surface loss. The relationship between these structural changes may serve as a predictor of engaging (off-track) or non-engaging (on-track) HSL, even after a surgical Bankart repair [259]. Di Giacomo *et al*. [260] described the on-track/off-track method using 3D CT, which was later validated for MRI, demonstrating moderate to high accuracy compared to arthroscopic engagement, with an overall accuracy of 84.2%, a sensitivity of 72.2%, and a specificity of 87.9% [110, 113, 115, 261, 262].

Recently, Yamamoto *et al*. [126] further analysed outcomes of patients with “subcritical bone loss” (between 13.5% and 20%) who underwent arthroscopic Bankart repair, and found two subgroups in the “on-track” group that showed different Western Ontario Shoulder Instability Index scores, depending on the location of the HSL. Radiologic glenoid track, however, has shown less inter-observer agreement and reliability compared to a standardized arthroscopic tracking method [121].

Additional evaluation methods have been developed, namely the Hill-Sachs interval/glenoid track ratio [113] and distance-to-dislocation [263]. The CSA, glenoid version, and AI also seem to be associated with anterior shoulder instability [116]. RC muscle imbalances may also be associated with shoulder instability, as evidenced by comparing RC muscle area in cross-sectional imaging [119, 120]. Finally, 3D MRI may also be used for glenoid bone stock evaluation in pre-surgical planning of shoulder arthroplasty [264].

### 3.2 Tendons

Tendon injury and rupture are common musculoskeletal disorders that can lead to significant functional impairment and morbidity. RC injuries are highly prevalent and represent one of the leading causes of shoulder pain and disability, accounting for more than 50% of shoulder pain cases. Women are more susceptible to RCT, RC tendinopathy, and shoulder impingement [265]. MRI has high sensitivity for detecting and characterizing small to massive RCTs, with good correlation to clinical examination and arthroscopy [76, 77]. Morphological and radiological parameters, however, show a weak correlation with shoulder function, pain levels, and clinical improvement over time [72, 89].

Identification of RCTs may be challenging, but fundamental in determining the appropriate treatment and preventing the development of RCT-associated GHJ arthropathy. RCTs disrupt the normal GHJ kinematics and promote the development of osteoarthritis. Infraspinatus tears seem to have the most significant effect on maintaining a normal position of the humeral head and preventing superior migration [266], with a recognized contribution by the teres minor and SSC tendons [267]. Partial SSC tears are underdiagnosed [268], and quantitative indicators, such as the fissure sign, thinning of the SSC tendon, and fluid collection at the coraco-glenoid arch, may help increase sensitivity and specificity [73]. Additionally, the teres minor muscle may develop compensatory hypertrophy in chronic SSC injuries, often accompanied with muscle atrophy [269].

MRI is also highly specific for diagnosing complete LHB tendon tears (99%), but partial tears and tendinosis have lower sensitivity (67.8%) and specificity (75.9%), according to a recent systematic review and meta-analysis [270].

### 3.3 Ligaments, Capsule, Cartilage, and Labrum

MRI enables a detailed qualitative assessment of the CAL and its attachments. Thickening, degeneration, and osteophyte formation within the CAL are recognized findings in shoulder impingement and RCT and have surgical implications [82, 87, 101, 106, 109, 271]. A recent study reported a relationship between decreased CAL thickness at the acromial undersurface and increasing RCT size, possibly due to repeated trauma or contact between both structures [91].

Although AC may be suspected based on MRI findings, a definitive diagnosis is made clinically. Characteristic signs include inferior glenohumeral ligament thickening and edema, coracohumeral ligament thickening, and rotator interval fat obliteration with edema [128, 272].

It is not uncommon for an orthopedic surgeon to order an MRA in cases of suspected capsulolabral lesions in young patients, or when ultrasound or conventional MRI is inconclusive in symptomatic patients who are unresponsive to conservative treatment. Different specificities and sensitivities have been documented in the literature comparing conventional MRI with MRA for RCTs and intra-articular lesions, such as Bankart lesions and superior labrum from anterior to posterior (SLAP) tear necessary to introduce the acronym [47, 125], with variable results. Nevertheless, MRA has been advocated as the most suitable technique for evaluation of shoulder instability, due to its increased sensitivity and specificity for labroligamentous lesions, as demonstrated in meta-analyses [50, 273].

The availability of high-field MRI systems and the advent of artificial intelligence (AI) techniques have significantly improved SNR and spatial resolution, thus questioning the need for MRA. Several institutions still utilize MRA in young patients where labral tears are suspected or conventional MRI is unremarkable.

MRA examination may be performed in the resting and so-called abduction external rotation (ABER) position (the palm is placed under the patient’s head at a 90º angle with the torso). The ABER position improves visualization of anterior-inferior labral detachments and posterosuperior impingement findings (posterior labral, joint capsule and RC lesions). However, it significantly increases imaging time due to patient repositioning and coil switching.

Despite ongoing debate, the ABER position has been shown to enhance sensitivity for detecting anterior-inferior capsulolabral lesions and joint capsule laxity in a functional position, which promotes multidirectional instability (MDI). However, it does not improve RCT detection sensitivity [62].

MRA findings consistent with MDI include capsular redundancy and increased joint capacity [124]. The gleno-capsular ratio can be assessed by dividing the distance between the most superior point of the glenoid and the most inferior point of the capsule by the distance between the most superior point of the glenoid and the most inferior point of the glenoid [70, 122]. A value above 1.42 is associated with MDI. Other measurements include rotator interval width and inferior labro-capsular distance.

MRA also performs well for RCT detection, showing good agreement with ultrasound [123] and higher sensitivity than MRI [125]. Nevertheless, a meta-analysis concluded that, while MRA offers slightly greater diagnostic accuracy, conventional MRI remains the preferred first-line imaging modality [55].

## 4 ADVANCED TECHNIQUES

### 4.1 Contrast Administration

Blood flow patterns around the shoulder joint are distinct in healthy patients and patients with frozen shoulder, ACor RC pathology [130, 132]. AC is categorically a clinical diagnosis, but MRI is often requested to exclude other conditions and identify suggestive direct or indirect signs.

Anecdotal reports have investigated changes in blood flow around the shoulder in patients with frozen shoulder, utilizing semiquantitative 3D dynamic-MRI evaluation [133, 134, 274]. The development of an abnormal cluster of blood flow at the rotator interval and axillary pouch (termed “burning sign”), as well as synovial enhancement, has been described [129, 130, 132]. Furthermore, evaluation of the coefficient of enhancement in these structures after shoulder manipulation under anesthesia and intra-articular corticosteroid or hyaluronate injection has shown a decrease in contrast enhancement, which supports the hypothesis that these are the main pathologic sites of frozen shoulder [134].

Sasanuma *et al*. [129] identified an association between shoulder stiffness (*i.e*., decreased ROM), increased pain, and a positive “burning sign” on the rotator interval and axillary pouch upon 3D dynamic MRI, thus describing a causative role of angiogenesis in the local development of inflammation [275].

Interestingly, a recent study compared the diagnostic performance of contrast-enhanced and non-enhanced MRI in patients with AC and found higher agreement and distinctly higher diagnostic performance in the contrast-enhanced group [19].

Notably, Lee *et al*. performed voxel-based 3D segmentation of the entire capsulo-synovium in contrast-enhanced shoulder MR images of patients with AC and identified a good inverse correlation with the severity of clinical impairments, specifically joint volume reduction and limited passive ROM [131].

An established relationship between pain and neovessel formation has led to the development of intravascular treatments. Okuno *et al*. reported the development of neovascularity and abnormal vessels around the shoulder joint in patients with chronic shoulder pain or AC, as documented on angiography, with successful pain relief following transarterial superselective embolization. The field of interventional radiology is rapidly expanding [276-278].

### 4.2 Radial Imaging

Radial sequences have proven invaluable in hip joint imaging, both in non-arthrographic and arthrographic MRI, enabling a detailed evaluation and description of labral and cartilage changes along their circumferential coverage. In the shoulder, radial sequences have been explored to provide cross-sectional slices perpendicular to the RC enthesis with the potential for increased accuracy in tendon tear detection [138] Fig. (**4A-F**).

The LHB tendon is a well-known source of shoulder pain, and the ability of MRI to detect partial long head of the biceps tendon LHB tears is average at best (sensitivity of 50% and specificity of 70%) [279]. Shibayama *et al*. compared the diagnostic accuracy of radial MRI with conventional MRI for partial tears of the LHBT and reported a higher sensitivity [21]. Similarly, Takeshima *et al*. evaluated the efficacy of biceps radial-slice MRI for diagnosing LHBT and pulley lesions, with promising results [20].

The performance of conventional MRI for RC assessment and MRA for capsulolabral and cartilage assessment is excellent; therefore, the added utility of radial sequences still needs to be demonstrated to justify broader or focused clinical application in the shoulder.

Regardless, Shibayama *et al*. [139] investigated the relationship between preoperative RCT size and postoperative RC integrity, finding that the preoperative tear area was independently correlated with the rate of retear after ARCR. Sasaki *et al*. [138] addressed RCT morphology characterization and found that radial MRI was better at describing tears with a length of ≥ 3 cm.

Honda *et al*. observed that radial evaluation of the RC was more adequate than coronal and axial oblique slices at the posterior portion (45º) [136]. Finally, Furukawa *et al*. reported the usefulness of radial images to detect SSC tears [135].

Despite the smaller size of the glenoid and its respective labrum compared to the acetabulum, Ogura *et al*. [137] validated a radial slice protocol that enabled precise assessment of injured glenoid labra and enabled more detailed assessment of the anteroinferior labrum.

### 4.3 3D Applications

Due to recent advances in MRI, it is now possible to generate 3D sequences with higher resolution and reduced imaging times, achieving detailed anatomic depiction and acceptable diagnostic accuracy. Advantages of 3D imaging over traditional 2D acquisitions include the ability to perform multiplanar reconstructions, create 3D models for surgical planning, and accurately measure bone loss/deformity without radiation exposure.

The most common applications of 3D imaging include the evaluation of glenoid bone loss and HSL in shoulder instability, glenoid characterization in arthroplasty planning [264], and less frequently, the determination of RCT shape and the identification of labral and cartilage lesions Fig. (**5A-E**).

A 3D dual echo-time T1-weighted fast low-angle shot (FLASH) sequence has been extensively studied and demonstrated similar accuracy to 3D CT [280]. VIBE sequences have also shown equivalence to 3D CT, and are more cost-effective than MRI with adjuvant CT acquisition [148]. Preoperative planning with 3D MRI does not appear to alter surgical management compared to 3D CT in patients with shoulder instability [151]. It is, in fact, a viable method for preoperative planning and graft selection in cases of glenoid bone loss [149].

Feuerriegel *et al*. generated simulated CT-like images and radiographs of the shoulder using a 3D T1-weighted echo MRI sequence in patients with shoulder pain, demonstrating perfect measurement inter- and intra-rater agreement [146]. A 3D MRI sequence resembling CT (FRACTURE) was compared to 3D CT for quantification of bone loss and morphologic measurements, with no significant differences [23]. Anterior glenoid lesion measurements are also feasible and resistant to low experience with 3D MRI reconstructions [26]. Moreover, glenoid deformity in GHJ arthritis and bone loss associated with joint instability are comparable to those observed in 3D CT [114, 147].

The cartilaginous surface of the GHJ was evaluated in cadaver specimens several decades ago. However, there is limited information regarding imaging studies, which are primarily based on 3D CT data, which only captures bony anatomy. A recent study has provided a detailed description of the distribution and thickness of GHJ cartilage using 3D MRI [141].

RCT morphology is an important imaging feature that should be reported by radiologists whenever possible, as it has significant implications for surgical planning. RCTs may have several configurations, namely crescent (length wider than width) or longitudinal (width wider than length) shapes, and different surgical approaches may be employed depending on the size, location and type of tear. Due to the curved orientation of the SSP and infraspinatus tendons, standard MRI sequences may be insufficient for the correct characterization of tear shape. 3D RC reconstructions may be achieved using post-processing software applied to coronal oblique T2-weighted sequences and manually segmented. A curved reconstruction of the RC using a specific postprocessing software accurately depicts the RCT morphology [140].

3D methods have been developed to assess the intramuscular spatial distribution of fatty infiltration in RCT [25, 142, 145] and after ARCR [24, 150]. Shoulder MRI, however, usually only captures the lateral third of the scapula and the RC muscles, limiting full muscle evaluation. Interestingly, a study published by Riem *et al*. developed a technique to extrapolate the entire 3D volume of all RC muscles using partial scans, showing a strong correlation with complete coverage [25]. Another study proposed simple mathematical equations to predict 3D RC muscle volumes based on cross-sectional areas, with low error [144].

A 3D gradient echo sequence (3D-MEDIC) has been evaluated to detect SLAP lesions and demonstrated higher sensitivity and negative predictive value than 2D FS PDWI [143]. Haider *et al*. [153] also found that 3D MRI had a stronger correlation with surgical findings compared to 2D MRI for detecting labral tears.

### 4.4 Fat Quantification

Physiological muscle fatty infiltration occurs with aging, and it also affects the RC, even in the absence of tendon injuries [157]. Importantly, after a significant RC injury, the musculotendinous unit retracts and undergoes atrophy or fatty infiltration, resulting in an irreversible loss of elasticity [281]. Fatty degeneration of RC muscles has been identified as an important predictor of surgical outcomes, and its development appears to be dependent on location, extent, and degree of musculotendinous junction retraction [282]. Goutallier developed a five-point CT-based grading scale to describe fatty infiltration of the shoulder muscles, which served as a semiquantitative method and has since been adopted for MRI [283]. The subjectivity underlying qualitative evaluation, as seen in Goutallier scoring, and its low reliability in clinical settings, have prompted the development of alternative techniques [284]. Distinguishing between intermuscular (between muscle groups) and intramuscular (within or between muscle fibers) fat is particularly important, as Goutallier scoring does not address this distinction.

The Dixon method separates water and fat signals with near-simultaneous paired in-phase and out-of-phase image acquisition. It achieves reliable fat saturation in areas subject to increased field inhomogeneities and outputs fat-only and water-only images. Dixon techniques allow voxel-wise quantification of fat content by generating fat fraction maps, in which the signal intensity of each pixel reflects the tissue’s fat percentage, and the resulting RC fat fraction has been shown to correlate with conservative treatment outcomes [27, 285], as well as postoperative failure and retear prediction [28, 29, 154, 286]. Isotropic multi-echo 3D-Dixon sequences with fat fraction maps have also been shown to quantify fatty infiltration in RC muscles accurately [158].

The iterative decomposition of water and fat with echo asymmetry and least-squares estimation (IDEAL) sequence is another water-fat imaging technique that correlates well with tendon tear severity and other clinical parameters [154, 159, 287].

These techniques demonstrate higher reliability and stronger clinical correlations than the traditional Goutallier classification [288]. Indeed, fat fraction has been demonstrated to correlate with Goutallier scores, size of RCTs, pain, symptom duration, and clinical function of the shoulder joint [287, 289], as well as failure [154] and muscle atrophy reversal [158] after RC repair. Karampinos *et al*. [156] found that RC fat-fraction measurements and muscle cross-sectional area correlate with morphological cartilage defects, cartilage T2 relaxation times, and clinical isometric strength measurements, linking fatty infiltration to functional impairment and the progression of osteoarthritis.

Addona *et al*. [161] estimated 3D SSP intramuscular fatty infiltration using a single 2D MRI slice, with a protocol that included a 6-point Dixon fat fraction map and a 6-point Dixon volumetric sequence, showing only a 1% difference between mean 2D and 3D SSP fat fractions. Similar results were described in another study comparing 3D and 2D fat-fraction of intramuscular fat in patients with medium to massive RCTs [160].

Gilbert *et al*. [155] compared the Goutallier system with quantitative MR spectroscopy (SPLASH) findings and reported only a fair correlation, noting that spectroscopic fat measurement allows precise quantification of fat content within a given ROI.

### 4.5 T2 Mapping

Detecting early osteoarthritic changes at the GHJ may influence treatment decisions for tendinopathy and tears, and help prevent progression to RC arthropathy [31]. Conventional MRI detects late-stage structural changes, such as extensive cartilage loss, osteophyte formation, subchondral sclerosis and cyst formation, but microscopic defects or early changes are inconspicuous.

T2 relaxation time of cartilage changes according to its biomatrix composition; collagen disruption and increased water content take place with cartilage degeneration. Quantitative transverse relaxation time (T2) mapping detects biochemical changes that precede macroscopic damage [165, 290, 291], and several studies have applied this technique to the knee. More recently, T2 values of GHJ cartilage have been evaluated in both asymptomatic individuals and patients with SSP lesions [31, 32], yielding notable insights into cartilage health Fig. (**6A,B**).

Biochemical composition of RC tendons has also been analyzed using T2 mapping techniques to evaluate tissue integrity, based on the same principles of tissue composition changes. Some studies have found good reliability in differentiating partial-thickness and full-thickness RCTs from normal tendons and tendinosis [164, 166].

Correlation of Goutallier staging with retear after ARCR has remained controversial, as some studies have identified it as a risk factor [167, 292, 293], while others have not observed such an association [294, 295]. In response, true quantitative evaluation methods have been developed [281, 296-298], including MR spectroscopy, the Dixon method, and CT, as previously described. The degree of muscle fatty degeneration appears to vary depending on the site of measurement in sagittal slices [163], which may partly explain the variability in results. Iijima *et al*. investigated the relationship between postoperative retear and preoperative fatty degeneration in 107 patients with massive RC tears using T2 values and reported significantly higher values in the retear group compared to the intact group [167]. Another study found smaller T2 values in patients with successful ARCR and suggested that fatty degeneration can be partially reversed [30].

### 4.6 UTE / ZTE

Conventional MRI sequences utilize echo times (TE) of several milliseconds, which are several orders of magnitude above the relaxation time (T2*) of certain tissues. The short relaxation times of tendons and their propensity to the magic angle effect due to their rich collagen content and fiber orientation have remained a challenging limitation in musculoskeletal MRI, with low sensitivity for tendinosis detection in conventional sequences [242, 299]. Cortical bone is also obscured, appearing as a signal void in conventional MRI due to its short T2 relaxation time.

Ultra-short time-to-echo (UTE) sequences are characterized by TE values less than 0.1 ms, making it possible to detect signal in structures with short T2, such as tendons and bone, before their signal decays to background levels Fig. (**7A-C**).

Investigation of UTE sequences in tendon morphology and pathology has garnered interest among research groups, and several studies have been carried out [33]. The most commonly evaluated tendons are the patellar and Achilles tendons. Regarding RC tendons, Ashir *et al*. compared RC tendons of healthy subjects with symptomatic tendinopathic patients [172]. Xie *et al*. [174] studied UTE-T2* values of tendons in patients subjected to ARCR, and found that patients with treated RCTs had inferior contralateral SSP tendon UTE-T2* values on the distal subregion compared to healthy controls. Xie *et al*. also compared T2* values between suture-bridge and single-row repair tendons and found that they were higher in the early postoperative period of repaired tendons using the suture-bridge technique, but these changes resolved over time [35].

Zhu *et al*. [169] evaluated *ex-vivo* tendon specimens and utilized the UTE magnetization transfer technique previously implemented in the Achilles tendon to minimize the magic angle effect, which was sensitive to RC tendon degeneration. Guo *et al*. specifically assessed if quantitative MRI techniques could detect abnormalities in SSP tendon samples treated with collagenase [171].

Zero echo time (ZTE) sequences, another type of short TE sequences, differ from UTE in that signal acquisition is performed almost immediately after application of the radiofrequency pulse (echo time = 8 usec) [168]. ZTE image post-processing has the ability to show contrast between soft tissue and bone comparable to that of CT, through the inverse-logarithmic rescaling of MR images [168]. Mello *et al*. compared 3D ZTE MRI with 3D CT for glenoid bone assessment and measured glenoid widths to find similar results with high intermodality and interrater agreement, with a bias of 1 mm or less for all measurements [173]. Post-processing times were also surprisingly shorter than conventional 3D gradient echo techniques (less than 1 minute versus 10-25 minutes). Notably, evaluation of Bankart lesions and other osseous pathologies of the shoulder is also feasible using TE sequences [34].

A study performed on 20 cadaveric shoulder specimens utilized 3D IR-UTE-Cones sequences and compared them with 3D and 2D CT, showing similar results in glenoid width measurement, and lower agreement compared to 2D T1-weighted imaging (WI) [170]. Yildiz *et al*. compared the CSA measurement on ZTE MRI scans with Grashey view radiographs and obtained significant differences, even with optimal positioning [175].

Finally, Puel *et al*. [300] compared the diagnostic performance of standard MR with the addition of ZTE images for the detection of RC calcific tendinopathy in 46 patients, showing some improvement in lesion detection.

### 4.7 Kinematic / Cine-MRI

Cine-MRI, also known as kinematic MRI, evaluates the dynamic features of body segments or organs and has been widely utilized to characterize cardiac function. In the musculoskeletal field, several techniques have been explored for investigational purposes, yielding interesting results.

The GHJ has the largest ROM of all the joints in the body, making it prone to instability and dislocation of traumatic or atraumatic origin. Joint stability is maintained through structural preservation of the capsulolabral complex and bone surfaces, which can be assessed with conventional MRI or MRA. However, there are dynamic stabilizers, such as the RC muscles and deltoid, which compress the humeral head against the center of the glenoid fossa [38, 301]. Assessment of dynamic stability and the effect of muscle contraction in different rotational positions contributes to further understanding of GHJ and the role of humeral translation in shoulder instability [38]. This technique provides a non-invasive evaluation of humeral translation at different velocities. In short, dynamic information can be acquired with two distinct methods: acquisition of incremental movement [302-304] or truly dynamic recording of shoulder movements [37, 162].

Hatta *et al*. performed a 3D morphometric analysis of the coracohumeral distance (CHD) in four arm positions in internal rotation (0º flexion, 45º, 90º and 90º with maximum horizontal adduction) and found that 3D-CHD was narrower in the arm position of flexion with horizontal abduction compared to 0º flexion [305].

Kenmoku *et al*. [37] compared the GHJ rotation among asymptomatic patients and patients with SAI using kinematic MRI and found that the GHJ axial rotation was restricted in patients with SAI syndrome (SIS) compared to controls, suggesting RC dysfunction and SSCP/infraspinatus imbalance.

Pierrart *et al*. [306] developed a real-time 3D MRI technique that enabled noninvasive monitoring of the kinematics of the shoulder complex during slow active arm elevation.

Tempelaere *et al*. [39] detected kinematic differences between groups of patients with various degrees of RCT and described a novel measurement, “looseness”, defined as translation of the humeral head on the glenoid during an abduction cycle (Xrange and Yrange).

Recently, faster MRI sequences have been applied, such as fast imaging employing steady-state acquisition (FIESTA) and true fast imaging with steady-state free precession (trueFISP), to analyze GHJ rotation in subacromial impingement [36] and capsulolabral structure in patients with shoulder instability [176].

The focus of dynamic studies has been on uniform motion over the last few decades; a recent study, however, examined the influence of motion velocity or acceleration on the translation of the humeral head. Matsui *et al*. [38] examined the intra-articular kinematics of humeral head position and translation during axial shoulder rotation at different rotational velocities using cine-MRI.

These techniques are currently not feasible in typical clinical settings and remain a growing field of research with interesting potential and a need for external validation. They offer unique potential for assessing shoulder kinematics, impingement, and instability under load-bearing or dynamic conditions, yet remain limited by low temporal resolution, motion artifacts, and the absence of validated acquisition standards.

### 4.8 Functional MRI

A variety of techniques have been developed to quantify muscle structure and function. MRI can evaluate muscle function through T2WI sequences, which serve as a quantitative index of activity [307-312]. Signal intensity changes due to increases in the relaxation time of tissue water can be measured to indicate exercise-induced activity of muscles. Exercise results in a slower decay of the muscle water signal, which causes an enhancement in signal intensity of the activated muscles.

Several authors have conducted studies to better understand the etiology of these changes, with explanations generally focusing on the accumulation of osmolytes in the cytoplasm during activity, which prolongs the relaxation time of muscle water. Changes in T2 signal from resting to postexercise states (T2 shift) exhibit a rapid decay, with a half-life of approximately 7 minutes, necessitating that the patient be scanned immediately following exercise. T2 levels typically remain elevated for up to 30 minutes after exercise [313].

Changes in T2 signal, however, may be limited if exercise is not performed until fatigue, as evidenced by Takeda *et al*. [314]. Tawara *et al*. [40] developed an alternative technique to detect RC muscle activity using spin-echo echo-planar imaging and showed high detectability of slight muscle activity induced by acute exercise.

Currently, research on this subject remains within the spectrum of physiological muscle activation and post-exercise evaluation. The validation of physiological changes is the basis for future research on muscle functional imbalances and/or changes in pathological conditions, guiding rehabilitation protocols or explaining treatment failure.

### 4.9 Protocol Optimization

Motion artifacts caused by patient breathing or involuntary motion are common and can affect the diagnostic value of images. In Cartesian scans, patient movement during acquisition of data in the center of k-space determines the image contrast and may result in bulky motion artifacts. Patient size also affects the quality of fat suppression and susceptibility artifacts.

Different sampling methods have been employed to minimize the effect of motion artifacts on image quality. The MultiVane (MV) technique is a motion-robust k-space data acquisition sequence based on radial k-space sampling, in which parallel data lines rotate around the center of k-space, allowing for the correction of spatial inconsistencies [179]. Manufacturers use a range of terms for sequences analogous to MultiVane, including BLADE, JET, PROPELLER, and RADAR. Kohli *et al*. compared the BLADE and rectilinear techniques using 3.0 T MRI and were able to produce images with improved quality and reduced motion degradation in the shoulder [181]. These techniques, however, may produce streak artifacts, compromise information on the four corners of the conventional square FOV and take 30 to 60% longer to acquire compared to conventional Cartesian turbo-spin echo (TSE) sequences [315-317].

Protocol optimization in MRI sequences needs to strike a delicate balance between speed, SNR, soft tissue contrast, and spatial resolution to provide excellent images with acceptable diagnostic quality. Despite the development of modern 1.5 and 3.0 T MRI scanners, slow acquisition times and consequent availability pose a challenge compared to CT and radiographs. This is particularly true in musculoskeletal imaging, which relies on TSE pulse sequences to maximize signal gain and detect fluid or edema, as well as provide excellent contrast of peri-articular soft tissues that have long T1 and short T2 values, such as ligaments, tendons, cartilage, menisci, and labra [178].

Optimization of TSE pulse sequences involves creating compact echo trains, shortening echo spacing, and ultimately resulting in faster sampling. Modern scanners are capable of reducing acquisition time by a factor of two with echo space shortening and by combining fast radiofrequency pulses, high-performance gradients, and high receiver bandwidth.

Recently, acceleration methods have been developed to achieve even faster scan times by a factor of four, such as parallel imaging, compressed sensing undersampling, and multi-slice acquisition, which have had a significant impact on musculoskeletal MRI.

Compressed SENSitivity Encoding (C-SENSE) is based on disproportionate undersampling of less contributory data in certain domains without significant loss of information and, subsequently, image quality. It is used to reconstruct full images from heavily undersampled k-space data, and its implementation has had a significant impact on the number of MRI examinations that can be performed in routine clinical practice [177].

Both SENSE and C-SENSE, as well as motion-correction techniques, may be combined to reduce scan times and increase spatial image resolution. A recent study evaluated the feasibility of combining C-SENSE and MV, which was found to be feasible in the shoulder joint, producing reliable C-SENSE MV sequences that have been implemented in daily practice [179].

Parallel imaging (PI) is a highly effective acceleration technique introduced to shorten scan times, and it is perhaps the most widely used and easiest to apply. There are several PI techniques available from different vendors. Acceleration in parallel imaging (PI) is achieved by sampling, for instance, every second Cartesian k-space line, which reduces scan time by half but results in a lower signal-to-noise ratio (SNR). Acceleration above fourfold in 2D or 3D pulse sequences is generally considered inappropriate due to a significant impact on SNR. In fact, PI acceleration can result in SNR reduction of up to 50-58% [318]. The CAIPIRINHA (Controlled Aliasing in Parallel Imaging Results in Higher Acceleration) [319] technique has been developed to address these limitations; this technique applies unique phase cycling patterns for the individual bands in a multiband excitation, resulting in less aliasing energy and improved multislice image quality. CAIPIRINHA-accelerated MRI allows 6-minute shoulder acquisitions with acceptable diagnostic performance compared to the standard 10-minute 2D TSE MRI [180, 182].

These acceleration methods, however, have limitations, such as low SNR and artifacts, that are being addressed with the help of AI. Current deep-learning algorithms using convolutional neural networks have demonstrated that faster imaging does not necessarily come at the cost of SNR reduction, and may actually provide images with higher diagnostic accuracy for certain shoulder conditions [218], while halving acquisition times.

### 4.10 AI

The integration of machine learning (ML) and AI in MRI systems is poised to transform the way radiologists work in the near future. Technologies are being developed to assist radiologists in identifying abnormalities, quantifying large amounts of data and information provided by MR images, and enhancing diagnostic performance overall. In musculoskeletal imaging, AI applications have been shown to excel in certain areas, such as segmentation, classification, lesion detection, and non-interpretive tasks.

#### 4.10.1 Protocol Optimization

**Fig. (8):**
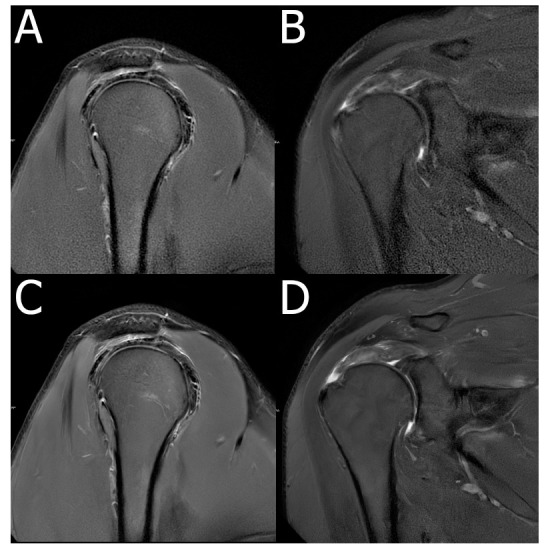
MRI with and without the Deep Resolve algorithm. Sagittal (**A**) and coronal (**B**) PROPELLER accelerated FS PDWI images. The addition of Deep Resolve algorithms (**C**, **D**) increases SNR, reduces motion artifacts, and yields overall better image quality.

Lengthy musculoskeletal MRIs with 30–45-minute protocols directly impact image quality due to increased motion and imaging artifacts, resulting in longer waiting times and leading to patient dissatisfaction and discomfort. Recent developments in Deep Learning (DL) models have introduced new reconstruction techniques for MRI acquisition that challenge the traditional trade-off between image quality and acquisition time. DL utilizes retrospectively undersampled data as similar as possible to fully sampled reference images from the same dataset [320]. Image reconstruction algorithms require substantially fewer sampled raw data to produce acceptable diagnostic MR images (Fig. **8A-D**). Indeed, DL algorithms have already been studied in shoulder MRI and were shown to reduce scan time and improve image quality in standard protocols [218, 220].

Even at optimal conditions, the shoulder region is prone to movement artifacts that may compromise image quality. Kaniewska *et al*. were able to improve image quality and reduce scan time utilizing accelerated PROPELLER sequences with DL post-processing [211]; similar results were achieved by Hahn *et al*. [216]. Koch *et al*. and Feuerriegel *et al*. developed a DL reconstruction method with denoising for shoulder MRI that showed improved image quality compared to conventional techniques [210, 215]. Numerous other studies have reported consistent improvement in SNR and contrast-to-noise ratio (CNR) with DL compared to conventional acquisition [210, 218, 219, 221, 321, 322].

Four-fold accelerated MRI of the knee has been found to be diagnostically interchangeable compared to standard twofold PI-accelerated acquisitions [323, 324]; in a similar fashion, four to eightfold accelerated TSE sequences of peripheral joints and lumbar spine have been performed with time savings of up to 75% [189, 213, 220, 325]. In the shoulder, DL has been applied to C-SENSE to further reduce scan time in 2D and 3D MRI [212, 214, 217].

In the shoulder, DL-based image reconstruction makes three and fourfold PI acceleration clinically feasible. Herrmann *et al*. compared the image quality and diagnostic performance of a fast 5-minute 2D TSE imaging with DL-reconstruction of the shoulder at 1.5 and 3.0 T. They demonstrated adequate feasibility with more than 50% reduced time and enhanced image quality [220].

In summary, DL acceleration has become an increasingly standard processing feature for TSE sequences, particularly in musculoskeletal imaging. Despite its widespread acceptance in clinical practice, research on its diagnostic performance is lacking.

#### 4.10.2 Segmentation

Segmentation is the process of classification of an image’s region of interest. Manual segmentation of bone or soft tissue structures is time-consuming and subject to inter-reader variability. Numerous studies have investigated the capabilities of AI algorithms to perform segmentation tasks with considerable success [326]. Such methodologies also pave the way for automatic analysis of anatomical structures for clinical purposes, and prefer quantitative imaging over qualitative analysis.

RC muscle segmentation remains a challenge due to the proximity and low-tissue contrast of adjacent muscles. Nonetheless, DL algorithms have been successfully developed to accurately detect RC muscles and perform automatic segmentation [185, 186, 189-191, 194]. Besides RC muscle 3D segmentation, muscle fatty infiltration was also quantified with good performance [25].

Automatic bone segmentation and 3D bone models have also been developed with DL algorithms, showing reliable and accurate results [184, 187, 188, 192, 193]. In a study by Wang *et al*., bone shape models derived from DL MRI reconstruction have been shown to be comparable to CT models [187].

#### 4.10.3 Diagnosis and Clinical Findings

Advanced segmentation techniques have been developed to identify pathological findings in shoulder MRI. Lee *et al*. proposed a DL model for RC tendon segmentation that successfully visualized RCTs through 3D reconstructed images and provided information about tear shape and size [190]. Several studies have demonstrated remarkable performance in diagnosing RCTs [189, 195, 197, 199, 200, 203-205, 207, 213, 327], with excellent diagnostic accuracy that may approach that of subspecialty-trained musculoskeletal radiologists [206, 208]. Zhang *et al*. [209] also developed an algorithm that can accurately predict RCT recurrence after ARCR before surgery.

Kim *et al*. [222] used a convolutional neural network (CNN) on shoulder MRI scans to diagnose RCTs, applied a weighted linear combination layer to handle sequential information, and weighted cross-entropy loss to address class imbalance, achieving a diagnostic accuracy of 87% and M-AUC score of 97%. CNNs have been successfully developed for the automatic measurement of the occupational ratio of the SSP muscle in the SSP fossa [196, 198] and for the assessment of SP muscle fatty infiltration [201], with high accuracy. Ni *et al*. developed a DL model to detect SLAP lesions in shoulder MRA, with results comparable to those of senior radiologists [202].

## 5 UNMET NEEDS IN SHOULDER PAIN IMAGING

Table **3** summarizes key research domains, the potential role of advanced MRI techniques, and the major validation challenges that currently limit clinical adoption. While significant advancements are being made to better understand shoulder pain, technical advances in MRI remain limited by the absence of reliable, quantitative, and reproducible biomarkers. There is currently limited standardization of advanced MRI sequences across vendors and institutions, and a lack of consensus on quantitative thresholds for tissue integrity. The validation of emerging MRI methods should follow a structured pathway, from technical feasibility to biological correlation and clinical integration.

Early studies must establish sequence reliability and repeatability through phantom studies, volunteer studies (using healthy subjects), and vendor comparison studies, with the aim of optimizing the acquisition protocol and validating technical parameters. Quantitative validation also relies on intra- and inter-scanner repeatability studies, cross-vendor calibration, and observer-blinded reproducibility studies.

Translational studies should confirm the biological relevance of MRI parameters through animal models of tendon degeneration, cadaveric studies, and/or surgical correlation studies. Clinical validation requires blinded diagnostic and prognostic studies, followed by multicenter standardization to harmonize acquisition parameters and analysis methods. This will establish standard operating procedures and consensus standards that enable integration in higher-level clinical trials as primary or secondary endpoints. Ultimately, widespread clinical adoption will depend not only on diagnostic validity but also on cost-effectiveness, seamless workflow integration, and the demonstration of improved clinical outcomes.

## CONCLUSION

MRI has revolutionized musculoskeletal evaluation by providing high-resolution images of osseous and soft tissue structures. Technological advancements have significantly improved MRI quality and increased its availability since the first scan was performed in 1977, positioning it at the forefront of medical practice. Musculoskeletal imaging, in particular, has seen exponential growth in demand over the last decade, attesting to the success of many proposed applications studied and validated by scientists and physicians.

Recent developments in biochemical composition analysis of tissue, kinematic evaluation, faster imaging techniques, improved image quality, and integration with AI will enable more accurate diagnoses and enhance patient treatment planning and outcomes in the near future, paving the way for precision medicine in musculoskeletal conditions.

However, many of these emerging techniques still lack large-scale validation and standardized protocols. Continued fundamental and technical research is essential to confirm their clinical utility, ensure reproducibility, and facilitate their safe and effective integration into routine musculoskeletal imaging practice.

## Figures and Tables

**Fig. (1):**
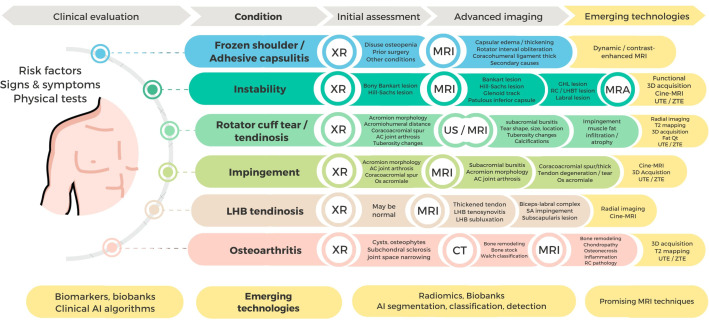
Clinical and imaging assessment of main shoulder pain conditions.

**Fig. (3):**
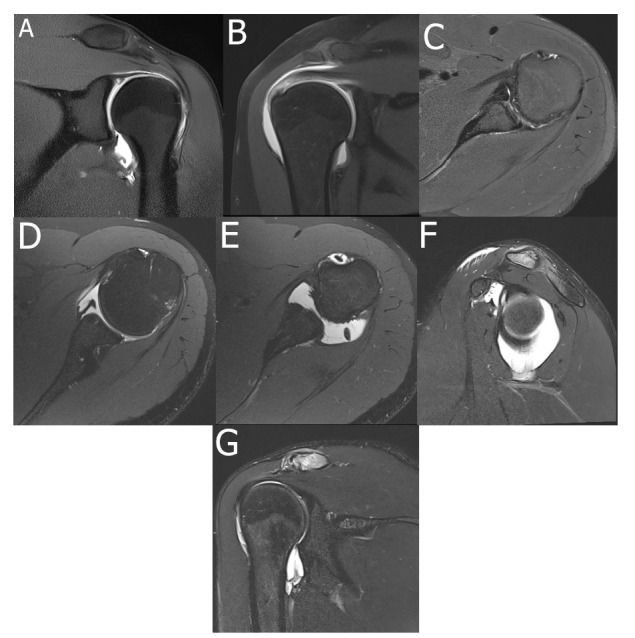
Magnetic resonance arthrography of the shoulder. **(A)** An MRA was performed on a 20-year-old male patient with shoulder instability and suspected capsular or labral structural damage after an inconclusive conventional shoulder MRI. Intra-articular gadolinium injection documents a deep sublabral fissure at the superior segment, compatible with a SLAP lesion on sagittal FS T2WI sequence. (**B**) Another patient who underwent MRA had a diagnosis of partial-thickness RC tear. However, after intra-articular gadolinium injection, there was filling of the subacromial-subdeltoid bursa, which was compatible with a full-thickness tear, as evidenced by coronal FS T1-weighted imaging (T1WI). (**C)** Conventional shoulder MRI in a 32-year-old patient with shoulder instability shows superimposing structures at the antero-inferior segment of the glenoid rim. Axial FS T2WI images reveal a bony Bankart with significant glenoid rim loss (**D**) and cortical stripping at the anterior-inferior segment (Perthes lesion) and a loose body (**E**) at the posterior recess. **(A)** FS T2WI sagittal (**F**) and coronal (**G**) MRA images of a 47-year-old patient with recurrent shoulder dislocation identify a capacious joint capsule with increased glenocapsular ratio and posterior laxity.

**Fig. (4):**
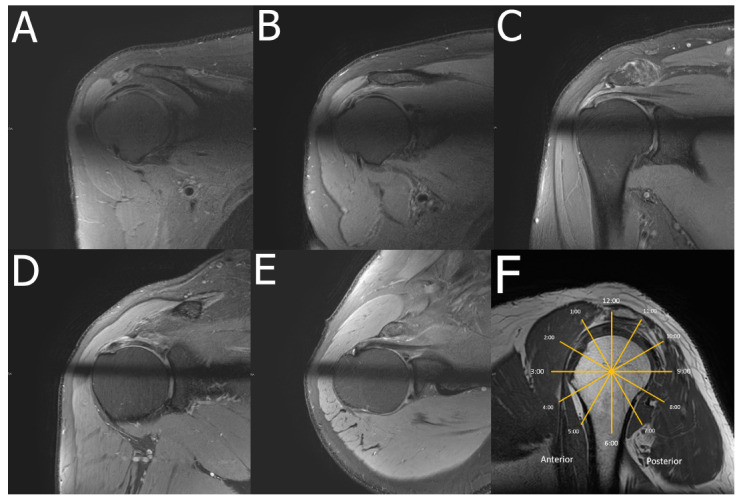
Radial imaging of the shoulder. A 68-year-old patient presented with chronic shoulder pain. Radial images show SSP tendinopathy and subacromial-subdeltoid bursitis. FS PDWI radial images are shown at 10 (**A**), 11 (**B**), 12 (**C**), 1 (**D**) and 3 (**E**) o’clock. Sagittal FS PDWI image for reference (**F**).

**Fig. (5):**
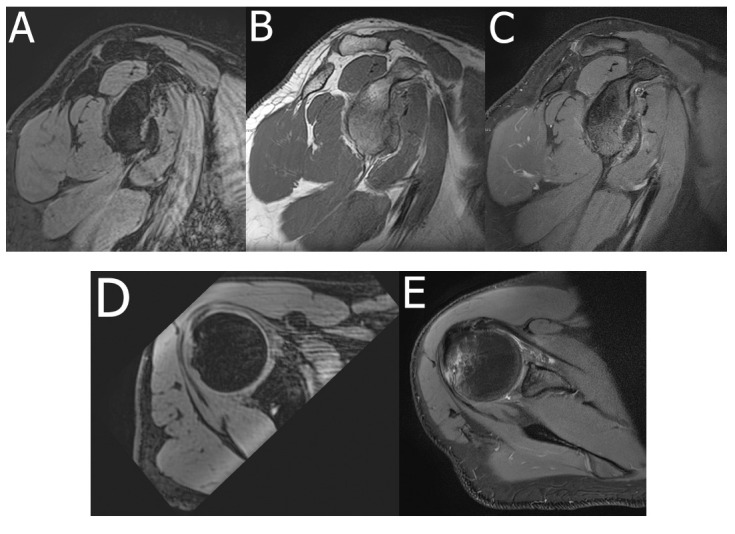
3D MRI for Glenoid track and Hill-Sachs lesion assessment. A 24-year-old patient presented with chronic recurrent shoulder dislocation. Sagittal 3D reconstruction increased spatial resolution for glenoid track assessment (**A**) compared to conventional 2D T1WI (**B**) or FS PDWI (**C**). Hill-Sachs lesion is also better depicted on 3D axial reconstruction (**D**) compared to FS PDWI (**E**).

**Fig. (6):**
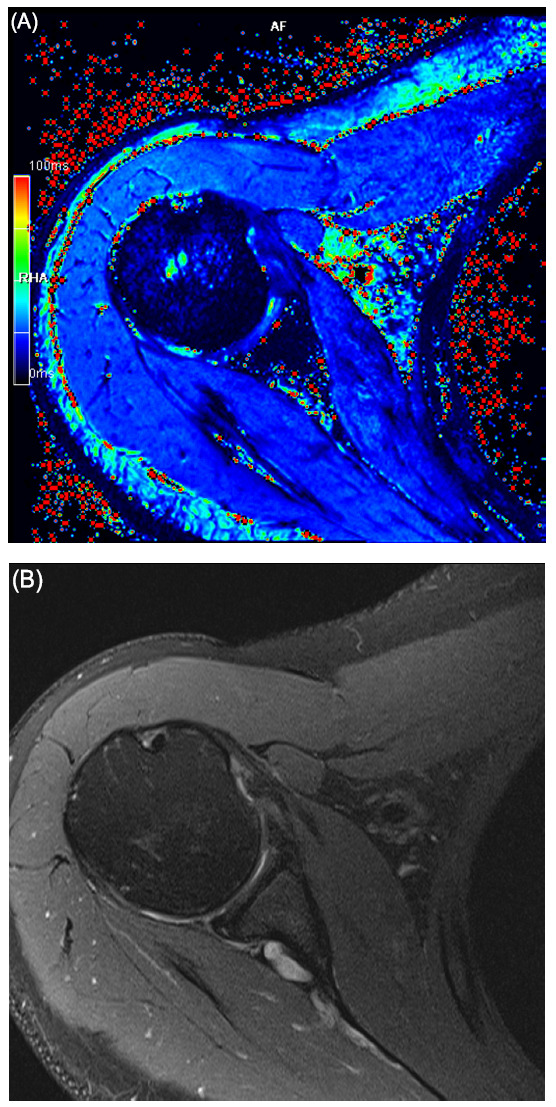
Cartilage mapping (T2*) of the shoulder. Images from the reference subject. (**A**) Axial T2 map image with color-scale and (**B**) corresponding PD FS axial sequence.

**Fig. (7):**
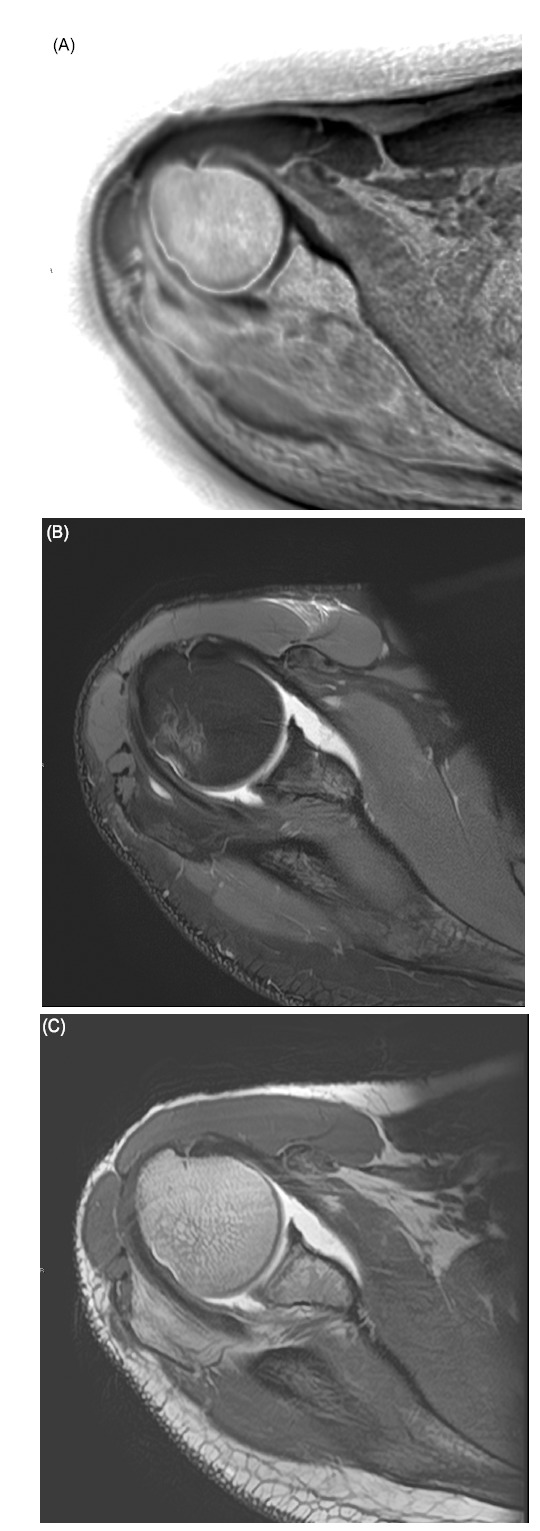
UTE MRI technique. A 22-year-old patient presented with a recent history of shoulder dislocation. The UTE image (**A**) delineates a small bone deformity at the posterior humeral head, compatible with a Hill-Sachs lesion. Axial FS PDWI (**B**) and T1WI (**C**) images are included for comparison.

**Table 1:** Demographics, pathophysiology, and clinical and relevant imaging findings for specific painful shoulder conditions.

**Condition**	**Age**	**Sex**	**Symptoms**	**Tests**	**Mechanism**	**Findings/Signs**
Shoulder impingement (subacromial) [4-7]	25-40	M>F	Intermittent mild pain with overhead activities over the lateral acromion (irradiation to the lateral arm) Pain lying on the affected side Night pain Loss of ROM	Hawkins-Kennedy test Neer’s test Jobe test Painful arc sign ER weakness	Dynamic disturbance during abduction and external rotation	Acromion configuration Coracoacromial ligament Acromioclavicular joint Critical shoulder angle Acromiohumeral distance Lateral edge of the acromion
Shoulder instability [8, 9]	10-35	M>F	Pain and instability with activity Slipping, popping, sliding Apprehension with horizontal abduction and lateral rotation Anterior or posterior pain	Load and shift test Apprehension test Relocation test Augmentation tests	Atraumatic (multidirectional instability), or previous shoulder dislocation Repetitive microtrauma Overuse/misuse	Glenoid bone loss Hill-Sachs lesion Labral injury Capsuloligamentous injury/predisposition Gleno-capsular ratio
Adhesive capsulitis / Frozen shoulder [10]	>45	F > M	Functional restriction of lateral rotation, abduction, and medial rotation	None	Primary - idiopathic Secondary - Insidious onset after trauma or surgery	Rotator cuff interval edema Axillary pouch edema/thickening Burning sign
Rotator cuff tendinosis/tear [11, 12]	30-50	F>M (tendinosis)	Weakness of abduction or rotation Tenderness over the rotator cuff Pain at the greater tuberosity Night pain	Drop-arm test Empty can test Jobe test Hawkins test WE weakness, WE lag sign IR weakness, IR lag sign Lift off sign	Pain and weakness after eccentric load Overuse/misuse	Tear type, size, shape, and location Fissure sign Coraco-glenoid arch fluid Tuberosity changes Calcifications
Long head of the biceps tendinosis/tear [13, 14]	50-60	-	Anterior shoulder pain over bicipital groove Easily fatigued shoulder	Speed test Uppercut test Yergason test	Overhead/throwing motions GHJ rotation LHBT instability Overuse/misuse	RC tendon injury Biceps-labrum complex injury
GHJ osteoarthritis [15-17]	>45 (M) >55 (F)	F > M*	Decreased ROM (ER, EA) Shoulder joint line tenderness Stiffness worsens with activity / improves with rest	Exclude other conditions	Primary (unknown) Secondary (post-inflammatory, postsurgical, posttraumatic, other)	Humeral cartilage thickness Glenoid cartilage thickness Bony changes

**Abbreviations:** * Not statistically significant. ER – external rotation. GHJ – glenohumeral joint. LHBT – long head of the biceps tendon. IR – internal rotation. ROM – range of motion.

**Table 2:** Performance, advantages, disadvantages, implementation tips, and key studies of advanced MRI techniques.

**Technique**	**Performance**	**Advantages**	**Disadvantages**	**Integration**	**Main Studies\Refs.**
MRA	Gold standard for evaluation of labral and capsuloligamentous structures	Excellent depiction of labral and capsular tears Well-established diagnostic accuracy	Minimally invasive May employ radiation	Complex instability and post-operative cases Use selectively as complementary to 3D MRI	Chang *et al*. [18]
Contrast administration	Enhances vascularized, inflamed or post-operative tissues	Improved detection of synovitis, adhesive capsulitis, and post-operative changes	Increased time, cost and patient discomfort Contraindications to intravenous contrast injection	Use for inflammatory or postoperative evaluation Indirect MRA when arthrography is not available	Erber *et al*. [19]
Radial imaging	Multiplanar imaging centered on the glenoid or humeral head	True anatomic visualization of structures in oblique orientation	Longer scan time Lacks standardization	Use to increase accuracy in LHB and conjoined tendon assessment	Takeshima *et al*. [20] Shibayama *et al*. [21]
3D applications	High-resolution isotropic acquisitions (*e.g*., 3D GRE, SPACE)	Allows multiplanar reformatting and quantitative analysis; Robust for anatomy depiction	Longer scan time Lower SNR Motion sensitivity Requires protocol optimization	Core sequences in modern shoulder MRI protocols Instability routine protocols Allows reformatting and AI-assisted post-processing	Daniels *et al*. [22] Cui *et al*. [23] Xu *et al*. [24] Riem *et al*. [25] Nizinski *et al*. [26]
Fat quantification	Quantifies intramuscular fat fraction and marrow composition	An objective, reproducible biomarker for muscle degeneration and surgical prognosis	Sensitive to inhomogeneity Variable cutoffs across systems Requires protocol optimization	Use as part of preoperative and postoperative evaluation (RC integrity, muscle quality)	Hochreiter *et al*. [27] Davis *et al*. [28] Monroe *et al*. [29]
T2 mapping	Quantitative evaluation of collagen integrity and water content	Detects early matrix changes and tendon healing potential	Time-consuming Variable reproducibility Requires protocol optimization Magic-angle sensitivity	Use in research or specialized centers for longitudinal cartilage and tendon assessment	Matsuki *et al*. [30] Lockard *et al*. [31] Lee *et al*. [32]
UTE / ZTE	Captures signal from short-T2 tissues (*e.g*., tendon, cartilage, calcification, bone)	Visualizes enthesis, fibrocartilage, and cortical bone not seen on conventional MRI	Limited availability Low SNR Requires protocol optimization	Emerging research tool Can complement standard MRI for bone interface visualization with CT-like images	Malhi *et al*. [33] Feuerriegel *et al*. [34] Xie *et al*. [35]
Kinematic / cine-MRI	Real-time or dynamic imaging of joint motion and instability	Direct visualization of kinematics and impingement under physiological movement	Low temporal resolution Motion artifacts No standardized protocol Requires hardware and protocol optimization	Potential adjunct for instability and impingement, not ready for routine clinical use	Ishii *et al*. [36] Kenmoku *et al*. [37] Matsui *et al*. [38] Tempelaere *et al*. [39]
Functional MRI	Evaluates the metabolic and biochemical composition of tissues	Provides insights into tissue energetics, inflammation, and muscle metabolism Potential biomarker for degeneration and response to therapy	Technically demanding Low spatial resolution Longer scan and post-processing time Limited reproducibility and validation	Research-only techniques with potential future role in assessing muscle metabolism, tendon degeneration, and early biochemical changes	Tawara *et al*. [40]
Protocol optimization	Integration of compressed sensing, deep-learning acceleration and adaptive protocols	Shorter scan times Better resolution Improved workflow efficiency	Vendor dependency Limited transparency and external validation	High potential for near-term adoption Implement scanner software updates as allowed Validate locally for diagnostic consistency	Chen *et al*. [41] Garcia *et al*. [42] Vosshenrich *et al*. [43] Rodriguez *et al*. [44]

**Abbreviations:** AI – artificial intelligence. CT – computed tomography. GRE - gradient recalled echo. LHBT – long head of the biceps tendon. MRI – magnetic resonance imaging. MRA – magnetic resonance arthrography. SNR – signal-to-noise ratio. SPACE - sampling perfection with application-optimized contrast using different flip-angle evolution. UTE – ultra-short time to echo. ZTE – zero-echo time.

**Table 3:** Unanswered research questions in shoulder pain conditions and current challenges for advanced MRI techniques.

	**Research Questions**	**Role of Advanced MRI Techniques**	**Current Challenges**
Etiology and susceptibility	• Which activities or morphologic features predispose to shoulder pain and structural damage? • What are the main sources of pain and functional disability? • Can imaging detect early structural or biomechanical vulnerability? • Can imaging play a role in periscapular dynamics and pathology?	• T2 mapping, UTE/ZTE and 3D isotropic imaging allow early detection of matrix and morphologic changes. • A combination of static MRI (morphology) with motion capture or cine-MRI to link mechanics to structure. • AI to mine large datasets for susceptibility patterns.	• Implementation remains largely research-based, with growing prototype vendor sequence development. • Most techniques remain in early feasibility stages; few have histopathologic correlation or test-retest reproducibility. • Lack of standardized protocols and multicenter comparability.
Diagnosis	• What morphological parameters best detect early structural damage or instability? • Can ultra-fast (3D) MRI achieve reliable diagnostic accuracy? • How can partial LHBT tears or tendinosis be better visualized? • Can we detect early structural damage at the GHJ?	• Radial imaging, 3D GRE, improves visualization of labral and bursal structures. • MRA remains the reference for capsulolabral evaluation. • UTE/ZTE imaging can capture short-T2 tissues (tendons, cartilage, and calcification). • AI-accelerated reconstructions enable higher-resolution or motion-robust imaging with shorter acquisition times.	• Some vendors provide clinically approved deep-learning reconstruction modules. • Diagnostic performance of techniques is largely based on small, single-center cohorts. No unified acquisition standards or large-scale reader reliability studies are conducted. • Limited cross-vendor calibration and reference phantoms.
Prognosis and natural history	• Can quantitative MRI biomarkers predict progression or surgical outcomes? • How do instability and impingement contribute to chronic progression in each patient? • How does muscle quality or cartilage status influence prognosis?	• Quantitative mapping and fat fraction analysis offer an objective assessment of tissue quality and degeneration over time. • AI-based radiomics may integrate multiple parameters to predict recovery or progression.	• Most studies are cross-sectional and lack longitudinal validation. • Quantitative thresholds for clinical decision-making remain undefined • AI models rarely undergo external validation or bias assessment.
Therapeutic intervention	• Which patients benefit from conservative versus surgical management? • How can imaging monitor tissue healing or treatment response?	• Quantitative MRI biomarkers can stratify patients for biologic or surgical interventions. • Cine-MRI can track mechanical outcomes after repair/rehabilitation. • MRA can assess capsular and labral integrity. • AI segmentation may facilitate automated follow-up/longitudinal comparisons.	• Most techniques remain in early feasibility stages, far from clinical implementation. • Limited data linking MRI metrics to clinical outcomes, with anecdotal trials, including imaging biomarkers. • Post-operative imaging protocols are not standardized.
Future directions	• Can we combine imaging modalities and AI to improve diagnostic performance and predict outcomes? • Can AI shorten scans or automatically analyze images? • Can we perform virtual functional assessments of shoulder ROM and impingement?	• Multimodal MRI-US-CT integration can merge structural and dynamic data and provide superior hybrid approaches. • Virtual cinematic rendering, real-time functional MRI, load-bearing imaging and AI-based automation (segmentation, lesion detection, acceleration) show high research potential.	• Most studies are preclinical or prototype-level. • No standardized datasets, interoperability solutions, or regulatory pathways. • Validation frameworks with representative datasets, regulatory approval, and reproducibility metrics through transparent research/development are urgently needed before clinical translation.

**Abbreviations:** AI – artificial intelligence. CT – computed tomography. GHJ – glenohumeral joint. LHBT – long head of the biceps tendon. MRI magnetic resonance imaging. MRA – magnetic resonance arthrography. US – ultrasound. UTE – ultra-short time to echo. ZTE – zero-echo time.

## LIST OF ABBREVIATIONS

2D: 2 dimensional
3D: 3 dimensional
3D-MEDIC: 3D multiple echo data image combination
ABER: Abduction and external rotation
AC: Adhesive capsulitis
AHD: Acromio-humeral distance/interval
AI: Artificial intelligence
AIn: Acromial index
AP: Antero-posterior
ARCR: Arthroscopic rotator cuff repair
BLADE: Balanced steady state free precession line acquisition with undersampling
CAIPIRINHA: Controlled aliasing in parallel imaging results in higher acceleration
CAL: Coraco-acromial ligament
CE: Contrast-enhanced
CNN: Convolutional neural network
CNR: Contrast-to-noise ratio
CSA: Critical shoulder angle
C-SENSE: Compressed sensing
CT: Computed tomography
CXR: Conventional x-ray
DL: Deep Learning
DTI: Diffusion tensor imaging
EPI: Echo-planar imaging
ER: External rotation
FI: Fatty infiltration
FLASH: Fast low-angle shot
FRACTURE: Fast-field-echo resembling a CT using restricted echo-spacing
FS: Fat-saturated
GCN: Graph convolutional network
GRE: Gradient recalled echo
HSL: Hill-Sachs lesion
IDEAL: Iterative decomposition of water and fat with echo asymmetry and least-squared estimation
LHB: Long head of the biceps
FSh: Frozen shoulder
GHJ: Glenohumeral joint
IR: Internal rotation
ISP: Infraspinatus
MDI: Multidirectional instability
MEDIC: Multiple echo data image combination
ML: Machine Learning
MMF: Macromolecular fraction
MPR: Multiplanar reconstruction
MRA: Magnetic resonance arthrography
MRI: Magnetic resonance imaging
MSSE: Multisection excitation by simultaneous spin-echo interleaving
NPV: Negative predictive value
OA: Osteoarthritis
PD: Proton density
PI: Parallel imaging
PROPELLER: Periodically rotated overlapping parallel lines with enhanced reconstruction
RC: Rotator cuff
RCT: Rotator cuff tear
ROM: Range of motion
SAI: Subacromial impingement
SIS: Subacromial impingement syndrome
SLAP: Superior labral anterior to posterior
SMS: Simultaneous multislice imaging
SNR: Signal-to-noise ratio
SPACE: Sampling perfection with application-optimized contrast using different flip angle evolution
SSP: Supraspinatus
T: Tesla
TE: Time-to-echo
TSE: Turbo spin echo
US: Ultrasound
UTE: Ultra-short time to echo
VIBE: Volumetric interpolated breath-hold examination
VRN: Value refinement network
WI: Weighted imaging
ZTE: Zero-echo time

## References

[1] Lucas J., van Doorn P., Hegedus E., Lewis J., van der Windt D. (2022). A systematic review of the global prevalence and incidence of shoulder pain.. BMC Musculoskelet. Disord..

[2] Tempelhof S., Rupp S., Seil R. (1999). Age-related prevalence of rotator cuff tears in asymptomatic shoulders.. J. Shoulder Elbow Surg..

[3] Shanahan E.M., Sladek R. (2011). Shoulder pain at the workplace.. Best Pract. Res. Clin. Rheumatol..

[4] Garving C., Jakob S., Bauer I., Nadjar R., Brunner U.H. (2017). Impingement syndrome of the shoulder.. Dtsch. Arztebl. Int..

[5] Hsiao M.S., Cameron K.L., Tucker C.J., Benigni M., Blaine T.A., Owens B.D. (2015). Shoulder impingement in the United States military.. J. Shoulder Elbow Surg..

[6] Singh B., Bakti N., Gulihar A. (2017). Current concepts in the diagnosis and treatment of shoulder impingement.. Indian J. Orthop..

[7] Ks D. (2019). Subacromial impingement syndrome of the shoulder: A musculoskeletal disorder or a medical myth?. Malays. Orthop. J..

[8] Rerko M.A., Pan X., Donaldson C., Jones G.L., Bishop J.Y. (2013). Comparison of various imaging techniques to quantify glenoid bone loss in shoulder instability.. J. Shoulder Elbow Surg..

[9] Bishop M.E., Patel H., Erickson B.J., Dodson C.C. (2022). Multidirectional instability in female athletes.. Ann. Joint.

[10] Barth K.A., Eliasberg C.D., Sutton K.M. (2022). Sex-specific considerations for shoulder instability and adhesive capsulitis in females.. J. Orthop. Orthop. Surg..

[11] Leong H., Fu S., He X., Oh J., Yamamoto N., Yung S. (2019). Risk factors for rotator cuff tendinopathy: A systematic review and meta-analysis.. J. Rehabil. Med..

[12] Saccomanno M.F., Lisai A., Romano A.M., Vitullo A., Pannone A., Spoliti M., Di Giunta A.C.C., Castricini R., Giordano M.C. (2023). High degree of consensus on diagnosis and management of rotator cuff tears: A Delphi approach.. Knee Surg. Sports Traumatol. Arthrosc..

[13] Krupp R.J., Kevern M.A., Gaines M.D., Kotara S., Singleton S.B. (2009). Long head of the biceps tendon pain: Differential diagnosis and treatment.. J. Orthop. Sports Phys. Ther..

[14] Raney E.B., Thankam F.G., Dilisio M.F., Agrawal D.K. (2017). Pain and the pathogenesis of biceps tendinopathy.. Am. J. Transl. Res..

[15] Prakash R., Gardner J.E., Petric U.B., Pathak R., Atem F., Jain N.B. (2024). Association of age and sex at onset with glenohumeral osteoarthritis.. Am. J. Phys. Med. Rehabil..

[16] Ansok C.B., Muh S.J. (2018). Optimal management of glenohumeral osteoarthritis.. Orthop. Res. Rev..

[17] Millett P.J., Gobezie R., Boykin R.E. (2008). Shoulder osteoarthritis: Diagnosis and management.. Am. Fam. Physician.

[18] Chang E.Y., Bencardino J.T., French C.N., Fritz J., Hanrahan C.J., Jibri Z., Kassarjian A., Motamedi K., Ringler M.D., Strickland C.D., Tiegs-Heiden C.A., Walker R.E.A. (2024). SSR white paper: Guidelines for utilization and performance of direct MR arthrography.. Skeletal Radiol..

[19] Erber B., Hesse N., Goller S., Gilbert F., Ricke J., Glaser C., Heuck A. (2024). Diagnostic performance and interreader agreement of individual and combined non-enhanced and contrast-enhanced MR imaging parameters in adhesive capsulitis of the shoulder.. Skeletal Radiol..

[20] Takeshima M., Morihara T., Furukawa R., Ito H., Kida Y., Sukenari T., Takahashi K. (2023). Efficacy of biceps-radial-slice magnetic resonance images for the diagnosis of biceps and pulley lesions.. J. Shoulder Elbow Surg..

[21] Shibayama Y., Hirose T., Sugi A., Mizushima E., Watanabe Y., Tomii R., Iba K., Yamashita T. (2022). Diagnostic accuracy of magnetic resonance imaging for partial tears of the long head of the biceps tendon in patients with rotator cuff tears.. JSES Int..

[22] Daniels S.P., Gyftopoulos S. (2021). 3D MRI of the shoulder.. Semin. Musculoskelet. Radiol..

[23] Cui D.D., Long Y., Yan Y., Li C., Yang Y.T., Zhong J.L., Yang R. (2024). Three-dimensional magnetic resonance imaging fast field echo resembling a computed tomography using restricted echo-spacing sequence is equivalent to 3-dimensional computed tomography in quantifying bone loss and measuring shoulder morphology in patients with shoulder dislocation.. Arthroscopy.

[24] Xu J., Liu B., Qiao Y., Ye Z., Su W., Zhao J. (2024). Longitudinal changes in overall 3D supraspinatus muscle volume and intramuscular fatty infiltration after arthroscopic rotator cuff repair.. J. Bone Joint Surg. Am..

[25] Riem L., Feng X., Cousins M., DuCharme O., Leitch E.B., Werner B.C., Sheean A.J., Hart J., Antosh I.J., Blemker S.S. (2023). A deep learning algorithm for automatic 3D segmentation of rotator cuff muscle and fat from clinical MRI scans.. Radiol. Artif. Intell..

[26] Nizinski J., Kaczmarek A., Antonik B., Rauhut S., Tuczynski P., Jakubowski F., Slawski J., Stefaniak J., Lubiatowski P. (2024). Reliability of glenoid measurements performed using Multiplanar Reconstruction (MPR) of Magnetic Resonance (MRI) in patients with shoulder instability.. Int. Orthop..

[27] Hochreiter B., Germann C., Feuerriegel G.C., Sutter R., Selman F., Gressl M., Ek E.T., Wieser K. (2024). Natural history of quantitative fatty infiltration and 3d muscle volume after nonoperative treatment of symptomatic rotator cuff tears.. J. Bone Joint Surg. Am..

[28] Davis D.L., Almardawi R., Henn R.F., Zhuo J., Mulligan M.E., Resnik C.S., Abdullah S.B., Al Khalifah H., Gilotra M.N., Hasan S.A., Gullapalli R.P. (2021). Correlation of quantitative versus semiquantitative measures of supraspinatus intramuscular fatty infiltration to shoulder range of motion and strength: A pilot study.. Curr. Probl. Diagn. Radiol..

[29] Monroe E.J., Flores S.E., Zhang A.L., Feeley B.T., Lansdown D.A., Ma C.B. (2020). Do Outcomes of arthroscopic subscapularis tendon repairs depend on rotator cuff fatty infiltration?. Orthop. J. Sports Med..

[30] Matsuki K, Sugaya H, Takahashi N, Tokai M, Hoshika S, Ueda Y. (2024). Fatty degeneration of the rotator cuff muscles improves in shoulders with successful arthroscopic rotator cuff repair: A prospective study using quantitative T2 mapping techniques, with 2-year follow-up.. JB JS Open Access.

[31] Lockard C.A., Nolte P.C., Gawronski K.M.B., Elrick B.P., Goldenberg B.T., Horan M.P., Dornan G.J., Ho C.P., Millett P.J. (2021). Quantitative T2 mapping of the glenohumeral joint cartilage in asymptomatic shoulders and shoulders with increasing severity of rotator cuff pathology.. Eur. J. Radiol. Open.

[32] Lee K.R., Ko S.Y., Choi G.M. (2019). Quantitative T2 mapping of articular cartilage of the glenohumeral joint at 3.0t in rotator cuff disease patients: The evaluation of degenerative change of cartilage.. Investig. Magn. Reson. Imaging.

[33] Malhi B.S., Jang H., Malhi M.S., Berry D.B., Jerban S. (2024). Tendon evaluation with ultrashort echo time (UTE) MRI: A systematic review.. Front. Musculoskelet. Disord..

[34] Feuerriegel G.C., Kronthaler S., Weiss K., Haller B., Leonhardt Y., Neumann J., Pfeiffer D., Hesse N., Erber B., Schwaiger B.J., Makowski M.R., Woertler K., Karampinos D.C., Wurm M., Gersing A.S. (2023). Assessment of glenoid bone loss and other osseous shoulder pathologies comparing MR-based CT-like images with conventional CT.. Eur. Radiol..

[35] Xie Y., Li X., Liu S., Hu Y., Chen Y., Liu S., Wu P., Tao H., Chen S. (2023). Quantitative magnetic resonance imaging–based tendon healing of different regions of the shoulder: Comparison between the suture-bridge and single-row techniques.. Orthop. J. Sports Med..

[36] Ishii D., Kenmoku T., Tazawa R., Nakawaki M., Nagura N., Muneshige K., Saito K., Takaso M. (2021). Limitation of the external glenohumeral joint rotation is associated with subacromial impingement syndrome, especially pain.. JSES Int..

[37] Kenmoku T., Matsuki K., Ochiai N., Sonoda M., Ishida T., Sasaki S., Tanaka Y., Nakawaki M., Nagura N., Tazawa R., Sasaki Y., Banks S.A., Takaso M. (2019). Comparison of glenohumeral joint rotation between asymptomatic subjects and patients with subacromial impingement syndrome using cine-magnetic resonance imaging: A cross-sectional study.. BMC Musculoskelet. Disord..

[38] Matsui K., Tachibana T., Nobuhara K., Uchiyama Y. (2018). Translational movement within the glenohumeral joint at different rotation velocities as seen by cine MRI.. J. Exp. Orthop..

[39] Tempelaere C., Pierrart J., Lefèvre-Colau M.M., Vuillemin V., Cuénod C.A., Hansen U., Mir O., Skalli W., Gregory T. (2016). Dynamic three-dimensional shoulder Mri during active motion for investigation of rotator cuff diseases.. PLoS One.

[40] Tawara N., Nishiyama A. (2021). Muscle functional MRI of exercise-induced rotator cuff muscles.. Investig. Magn. Reson. Imaging.

[41] Chen W., Lim L.J.R., Lim R.Q.R., Yi Z., Huang J., He J., Yang G., Liu B. (2024). Artificial intelligence powered advancements in upper extremity joint MRI: A review.. Heliyon.

[42] Velasquez Garcia A., Hsu K.L., Marinakis K. (2024). Advancements in the diagnosis and management of rotator cuff tears. The role of artificial intelligence.. J. Orthop..

[43] Vosshenrich J., Koerzdoerfer G., Fritz J. (2024). Modern acceleration in musculoskeletal MRI: Applications, implications, and challenges.. Skeletal Radiol..

[44] Rodriguez H.C., Rust B., Hansen P.Y., Maffulli N., Gupta M., Potty A.G., Gupta A. (2023). Artificial intelligence and machine learning in rotator cuff tears.. Sports Med. Arthrosc. Rev..

[45] Cagnie B., Elliott J., O’Leary S., D’Hooge R., Dickx N., Danneels L. (2011). Muscle functional MRI as an imaging tool to evaluate muscle activity.. J. Orthop. Sports Phys. Ther..

[46] Saliken D.J., Bornes T.D., Bouliane M.J., Sheps D.M., Beaupre L.A. (2015). Imaging methods for quantifying glenoid and Hill-Sachs bone loss in traumatic instability of the shoulder: A scoping review.. BMC Musculoskelet. Disord..

[47] McGarvey C., Harb Z., Smith C., Houghton R., Corbett S., Ajuied A. (2016). Diagnosis of rotator cuff tears using 3-Tesla MRI versus 3-Tesla MRA: A systematic review and meta-analysis.. Skeletal Radiol..

[48] Gold J.E., Hallman D.M., Hellström F., Björklund M., Crenshaw A.G., Mathiassen S.E., Barbe M.F., Ali S. (2017). Systematic review of quantitative imaging biomarkers for neck and shoulder musculoskeletal disorders.. BMC Musculoskelet. Disord..

[49] Gottsegen C.J., Merkle A.N., Bencardino J.T., Gyftopoulos S. (2017). Advanced MRI techniques of the shoulder joint: Current applications in clinical practice.. AJR Am. J. Roentgenol..

[50] Ajuied A., McGarvey C.P., Harb Z., Smith C.C., Houghton R.P., Corbett S.A. (2018). Diagnosis of glenoid labral tears using 3-tesla MRI *vs*. 3-tesla MRA: A systematic review and meta-analysis.. Arch. Orthop. Trauma Surg..

[51] Pesquer L., Borghol S., Meyer P., Ropars M., Dallaudière B., Abadie P. (2018). Multimodality imaging of subacromial impingement syndrome.. Skeletal Radiol..

[52] Alaia E.F., Subhas N., Shoulder M.R. (2020). Shoulder MR imaging and MR arthrography techniques.. Magn. Reson. Imaging Clin. N. Am..

[53] Maio M., Sarmento M., Moura N., Cartucho A. (2019). How to measure a Hill–Sachs lesion: A systematic review.. EFORT Open Rev..

[54] Loriaud A., Bise S., Meyer P., Billaud A., Dallaudiere B., Silvestre A., Pesquer L. (2020). Critical shoulder angle: What do radiologists need to know?. Skeletal Radiol..

[55] Liu F., Cheng X., Dong J., Zhou D., Han S., Yang Y. (2020). Comparison of MRI and MRA for the diagnosis of rotator cuff tears.. Medicine.

[56] Jerban S., Chang D.G., Ma Y., Jang H., Chang E.Y., Du J. (2020). An update in qualitative imaging of bone using ultrashort echo time magnetic resonance.. Front. Endocrinol..

[57] Vopat M.L., Hermanns C.A., Midtgaard K.S., Baker J., Coda R.G., Cheema S.G., Tarakemeh A., Peebles L., Vopat B.G., Provencher M.T. (2021). Imaging modalities for the glenoid track in recurrent shoulder instability: A systematic review.. Orthop. J. Sports Med..

[58] Familiari F., Galasso O., Massazza F., Mercurio M., Fox H., Srikumaran U., Gasparini G. (2022). Artificial intelligence in the management of rotator cuff tears.. Int. J. Environ. Res. Public Health.

[59] Thacher R.R., Retzky J.S., Dekhne M.S., Oquendo Y.A., Greditzer H.G. (2023). Current concepts in the measurement of glenohumeral bone loss.. Curr. Rev. Musculoskelet. Med..

[60] Oladimeji A.E., Amoo-Achampong K., Ode G.E. (2024). Impact of critical shoulder angle in shoulder pathology: A current concepts review.. JSES Int..

[61] Gupta P., Haeberle H.S., Zimmer Z.R., Levine W.N., Williams R.J., Ramkumar P.N. (2023). Artificial intelligence-based applications in shoulder surgery leaves much to be desired: A systematic review.. JSES Rev. Rep. Tech..

[62] Altmann S., Jungmann F., Emrich T., Jezycki T., Kreitner K.F. (2023). ABER position in direct MR arthrography of the shoulder: Useful adjunct or waste of imaging time?. Röfo Fortschr. Geb. Röntgenstr. Neuen Bildgeb. Verfahr..

[63] Keeling L.E., Wagala N., Ryan P.M., Gilbert R., Hughes J.D. (2023). Bone loss in shoulder instability: Putting it all together.. Ann. Joint.

[64] Eckers F., Loske S., Ek E.T., Müller A.M. (2023). Current understanding and new advances in the surgical management of reparable rotator cuff tears: A scoping review.. J. Clin. Med..

[65] Horowitz E.H., Aibinder W.R. (2023). Shoulder Impingement Syndrome.. Phys. Med. Rehabil. Clin. N. Am..

[66] Feuerriegel G.C., Sutter R. (2024). Managing hardware-related metal artifacts in MRI: Current and evolving techniques.. Skeletal Radiol..

[67] Tisherman R.T., Bulleit C., Champagne A.A., Fatora G.C., Lau B.C. (2024). There is high variability in quantitative measurement techniques in glenohumeral capsular measurements for shoulder instability: A systematic review.. Knee Surg. Sports Traumatol. Arthrosc..

[68] Ibounig T., Sanders S., Haas R., Jones M., Järvinen T.L.N., Taimela S., Docking S., Rämö L., Buchbinder R. (2024). Systematic review of shoulder imaging abnormalities in asymptomatic adult shoulders (SCRUTINY): Abnormalities of the glenohumeral joint.. Osteoarthr. Cartil..

[69] Housset V., Ho S.W.L., Lädermann A., Phua S.K.A., Hui S.J., Nourissat G. (2024). Multidirectional instability of the shoulder: A systematic review with a novel classification.. EFORT Open Rev..

[70] Rawal A., Eckers F., Lee O.S.H., Hochreiter B., Wang K.K., Ek E.T. (2024). Current evidence regarding shoulder instability in the paediatric and adolescent population.. J. Clin. Med..

[71] Darbandi A., Credille K., Darbandi A., Hevesi M., Dandu N., Bodendorfer B.M. (2024). Fatty infiltration, tear size, and retraction size are significant risk factors for retear following arthroscopic rotator cuff repair: A systematic review.. Arthroscopy.

[72] Cauchon A.M., Tétreault P., Bascans C., Skalli W., Hagemeister N. (2020). Morphologic and radiologic parameters correlating to shoulder function at diagnosis for patients with rotator cuff tear.. J. Shoulder Elbow Surg..

[73] Wang Q., Zhao J., Zhou S., Lv Y., Liu X., Yang H. (2022). Quantitative MRI indicators and features for partial subscapularis tendon tears on conventional shoulder MRI.. Insights Imaging.

[74] Ma Q., Sun C., Gao H., Cai X. (2022). The combined utilization of predictors seems more suitable to diagnose and predict rotator cuff tears.. BMC Musculoskelet. Disord..

[75] Minici R., Mercurio M., Iannò B., Galasso O., Gasparini G., Laganà D. (2023). Advantages of the use of axial traction magnetic resonance imaging (MRI) of the shoulder in patients with suspected rota-tor cuff tears: An exploratory pilot study.. Healthcare.

[76] Fazal Gafoor H., Jose G.A., Mampalli Narayanan B. (2023). Role of magnetic resonance imaging (MRI) in the diagnosis of rotator cuff injuries and correlation with arthroscopy findings.. Cureus.

[77] Gowda C.S., Mirza K., Galagali D.A. (2024). Rotator cuff tears: Correlation between clinical examination, magnetic resonance imaging and arthroscopy.. Cureus.

[78] Ben H., Kholinne E., Guo J., Park J.Y., Ryu S.M., Koh K.H., Jeon I.H. (2024). Preoperative magnetic resonance imaging rotator cuff tendon stump classification correlates with the surgical outcomes following superior capsular reconstruction.. J. Shoulder Elbow Surg..

[79] Zhang H., Zhang Q., Li Z.L. (2019). Coracohumeral index and coracoglenoid inclination as predictors for different types of degenerative subscapularis tendon tears.. Int. Orthop..

[80] Watson A.C., Jamieson R.P., Mattin A.C., Page R.S. (2019). Magnetic resonance imaging based coracoid morphology and its associations with subscapularis tears: A new index.. Shoulder Elbow.

[81] Zaid M.B., Young N.M., Pedoia V., Feeley B.T., Ma C.B., Lansdown D.A. (2019). Anatomic shoulder parameters and their relationship to the presence of degenerative rotator cuff tears and glenohumeral osteoarthritis: A systematic review and meta-analysis.. J. Shoulder Elbow Surg..

[82] Kim J.H., Min Y.K., Gwak H.C., Kim C.W., Lee C.R., Lee S.J. (2019). Rotator cuff tear incidence association with critical shoulder angle and subacromial osteophytes.. J. Shoulder Elbow Surg..

[83] Liu H.X., Xu X.X., Xu D.L., Hu Y.Z., Pan X.Y., Yu Z., Xu Y.J. (2020). The acromion–greater tuberosity impingement index: A new radiographic measurement and its association with rotator cuff pathology.. J. Orthop. Surg..

[84] Seo J.B., Kim S.J., Ham H.J., Kwak K.Y., Yoo J. (2020). New predictors for subscapularis tear: Coraco-lesser tuberosity angle, lesser tuberosity angle, and lesser tuberosity height.. Orthop. Traumatol. Surg. Res..

[85] Vaz A., Reifegerste C.P., Trippia C.R., Linhares L.S., Trindade F.B., Thomaz J.E. (2020). Effect of the acromial inferolateral tilt on subacromial impingement syndrome: A retrospective magnetic resonance imaging assessment.. Radiol. Bras..

[86] Joo Y., Cho H.R., Kim Y.U. (2020). Evaluation of the cross-sectional area of acromion process for shoulder impingement syndrome.. Korean J. Pain.

[87] İncesoy M.A., Yıldız K.İ., Türk Ö.İ., Akıncı Ş., Turgut E., Aycan O.E., Bayhan I.A. (2021). The critical shoulder angle, the acromial index, the glenoid version angle and the acromial angulation are associated with rotator cuff tears.. Knee Surg. Sports Traumatol. Arthrosc..

[88] Hufeland M., Brusis C., Kubo H., Grassmann J., Latz D., Patzer T. (2021). The acromiohumeral distance in the MRI should not be used as a decision criterion to assess subacromial space width in shoulders with an intact rotator cuff.. Knee Surg. Sports Traumatol. Arthrosc..

[89] Ferenczi A., Petrover D., Nectoux R., Orcel P., Laredo J.D., Beaudreuil J. (2022). Clinical and MRI outcomes of subacromial impingement syndrome with conservative treatment: A 21-month prospective study.. Acta Orthop. Belg..

[90] Obaid H., Mondal P., Sims L., Shepel M., Vassos N. (2022). Coracoclavicular bursal changes on MRI: A diagnostic consideration in patients with shoulder pain and reduced coracoclavicular distance.. Skeletal Radiol..

[91] Miyake S., Tamai M., Takeuchi Y., Izaki T., Arashiro Y., Shibata Y., Shibata T., Yamamoto T. (2022). Alteration of coracoacromial ligament thickness at the acromial undersurface in patients with rotator cuff tears.. JSES Int..

[92] El-Amin S.F., Maffulli N., Mai M.C., Rodriguez H.C., Jaso V., Cannon D., Gupta A. (2022). Coracoid impingement and morphology is associated with fatty infiltration and rotator cuff tears.. J. Clin. Med..

[93] Yu J., Xie P., Liu K., Sun Y., Zhang J., Zhu H., Chen Y. (2022). Identification of diagnostic magnetic resonance imaging findings in 47 shoulders with subcoracoid impingement syndrome by comparison with 100 normal shoulders.. Med. Sci. Monit..

[94] Xie L., Xu X., Ma B., Liu H. (2022). A high acromion-greater tuberosity impingement index increases the risk of retear after arthroscopic rotator cuff repair.. J. Orthop. Surg..

[95] Smith G.C.S., Liu V. (2022). High critical shoulder angle values are associated with full-thickness posterosuperior cuff tears and low values with primary glenohumeral osteoarthritis.. Arthroscopy.

[96] Kocadal O., Tasdelen N., Yuksel K., Ozler T. (2022). Volumetric evaluation of the subacromial space in shoulder impingement syndrome.. Orthop. Traumatol. Surg. Res..

[97] Kucukciloglu Y., Aydın D. (2022). Relationship between radiological measurement of subcoracoid impingement and subscapularis tendon lesions.. Clin. Orthop. Surg..

[98] Caffard T., Kralewski D., Ludwig M., Dornacher D., Fuchs M., Kappe T., Reichel H., Sgroi M. (2023). High acromial slope and low acromiohumeral distance increase the risk of retear of the supraspinatus tendon after repair.. Clin. Orthop. Relat. Res..

[99] Jäschke M., Köhler H.C., Weber M.A., Tischer T., Hacke C., Schulze C. (2021). Subacromial impingement syndrome: Association of multiple magnetic resonance imaging parameters with shoulder function and pain.. Arch. Orthop. Trauma Surg..

[100] Ting D.S., Yang J., Lin K.H., Wang T.G., Lin J.J. (2023). Alteration in coracohumeral ligament and distance in people with symptoms of subcoracoid impingement.. BMC Musculoskelet. Disord..

[101] Chuang H.C., Hong C.K., Hsu K.L., Kuan F.C., Chen Y., Yen J.Z., Chiang C.H., Chang H.M., Su W.R. (2023). Association of coracoacromial ligament degeneration with rotator cuff tear patterns and retear rate.. Orthop. J. Sports Med..

[102] Fallahpour N., Jamalipour Soufi G., Jamalipour Soufi K., Hekmatnia A. (2023). Evaluation of the acromion variants in MRI and their association with rotator cuff injuries in non-traumatic patients.. J. Orthop..

[103] Çeti̇nkaya M., Kaptan A.Y., Ulucaköy C., Orhan Ö., Topal M., Ayanoğlu T., Kanatli U. (2023). Is it the subcoracoid impingement or the subacromial impingement that tears the subscapularis tendon? A comparison of the MRI findings of the operated and healthy shoulders of the patients.. Turk. J. Med. Sci..

[104] Çağlar C., Akçaalan S., Akkaya M., Doğan M. (2023). Does morphology of the shoulder joint play a role in the etiology of rotator cuff tear?. Curr. Med. Imaging.

[105] Schiefer M., Naliato E., Oliveira R., Carmo L.T.D., Fontenelle C.R.D.C., Motta Filho G.D.R. (2023). MRI is a reliable method for measurement of critical shoulder angle and acromial index.. Rev. Bras. Ortop..

[106] Lopez R., Singh J., Ghoraishian M., Nicholson T., Gates S., Namdari S. (2024). Anatomic factors associated with degeneration and fraying of the coracoacromial ligament.. Clin. Shoulder Elbow.

[107] Mi Y., Lin Y., Cheng B. (2024). Magnetic resonance imaging based coracoid process morphology and its associations with isolated subscapularis tendon tears in Chinese patients.. Jt. Dis. Relat. Surg..

[108] Sakdapanichkul C., Chantarapitak N., Kasemwong N., Suwanalai J., Wimolsate T., Jirawasinroj T., Sakolsujin T., Kongmalai P. (2024). Transcending patient morphometry: Acromiohumeral interval to glenoid ratio as a universal diagnostic tool for massive rotator cuff tears.. Clin. Orthop. Surg..

[109] Özdemir F., Ayanoğlu T., Dağıstan E., Kalaycıoğlu O., Çelik İ., Kalfaoğlu M.E., Kanatlı U. (2024). Can coracoacromial ligament degeneration be evaluated with preoperative MRI?. Acta Radiol..

[110] Locher J., Wilken F., Beitzel K., Buchmann S., Longo U.G., Denaro V., Imhoff A.B. (2016). Hill-sachs off-track lesions as risk factor for recurrence of instability after arthroscopic bankart repair.. Arthroscopy.

[111] Stillwater L., Koenig J., Maycher B., Davidson M. (2017). 3D-MR *vs*. 3D-CT of the shoulder in patients with glenohumeral instability.. Skeletal Radiol..

[112] Vopat B.G., Cai W., Torriani M., Vopat M.L., Hemma M., Harris G.J. (2018). Measurement of glenoid bone loss with 3-D MRI: A matched computed tomography analysis.. Arthroscopy.

[113] Yang T.C., Chen K.H., Chiang E.R., Chang M.C., Ma H.L. (2018). Using the “Hill–Sachs interval to glenoid track width ratio” for prediction of recurrent instability after arthroscopic Bankart repair.. Orthop. Traumatol. Surg. Res..

[114] Lansdown D.A., Cvetanovich G.L., Verma N.N., Cole B.J., Bach B.R., Nicholson G. (2019). Automated 3-D MRI allows for accurate evaluation of glenoid bone loss compared with 3-D CT.. Arthroscopy.

[115] Dyrna F.G.E., Ludwig M., Imhoff A.B., Martetschläger F. (2021). Off-track Hill–Sachs lesions predispose to recurrence after nonoperative management of first-time anterior shoulder dislocations.. Knee Surg. Sports Traumatol. Arthrosc..

[116] Karahan N., Yilmaz B., Öztermeli A., Kaya M., Duman S., Derin Cicek E.E. (2021). Evaluation of critical shoulder angle and acromion index in patients with anterior shoulder instability and rotator cuff tear.. Acta Orthop. Traumatol. Turc..

[117] Sgroi M., Huzurudin H., Ludwig M., Zippelius T., Reichel H., Kappe T. (2022). MRI allows accurate measurement of glenoid bone loss.. Clin. Orthop. Relat. Res..

[118] Cohn M.R., DeFroda S.F., Huddleston H.P., Williams B.T., Singh H., Vadhera A., Garrigues G.E., Nicholson G.P., Yanke A.B., Verma N.N. (2022). Does native glenoid anatomy predispose to shoulder instability? An MRI analysis.. J. Shoulder Elbow Surg..

[119] Ishikawa H., Smith K.M., Wheelwright J.C., Christensen G.V., Henninger H.B., Tashjian R.Z., Chalmers P.N. (2023). Rotator cuff muscle imbalance associates with shoulder instability direction.. J. Shoulder Elbow Surg..

[120] Kim D.H., Kim J.H., Cho C.H. (2024). Rotator cuff muscle imbalance in patients with chronic anterior shoulder instability.. Diagnostics.

[121] Bhatia D.N., Malviya P. (2024). How does dynamic arthroscopic tracking compare with radiologic glenoid track for identification of on- and off-track lesions in anterior shoulder instability?. J. Shoulder Elbow Surg..

[122] Park K.J., Jeong H.S., Park J.K., Cha J.K., Kang S.W. (2019). Evaluation of inferior capsular laxity in patients with atraumatic multidirectional shoulder instability with magnetic resonance arthrography.. Korean J. Radiol..

[123] Wengert G.J., Schmutzer M., Bickel H., Sora M.C., Polanec S.H., Weber M., Schueller-Weidekamm C. (2019). Reliability of high-resolution ultrasound and magnetic resonance arthrography of the shoulder in patients with sports-related shoulder injuries.. PLoS One.

[124] Celentano A., Porta M., Calvi M., Basile G., Aliprandi A., Genovese E.A. (2022). Magnetic resonance arthrography in patients with multidirectional instability: Could inferior capsulsar width be considered the cornerstone in the diagnosis of non-traumatic shoulder instability?. Skeletal Radiol..

[125] Dwivedi A., Sharma R., Sharma A. (2024). Evaluation of shoulder injuries: A comparative study of imaging by Magnetic Resonance Imaging (MRI) and Magnetic Resonance Arthrography (MRA).. J. Pharm. Bioallied Sci..

[126] Yamamoto N., Aizawa T., Itoi E. (2024). Glenoid track and subcritical Hill-Sachs lesion.. JSES Int..

[127] Ozel O., Hudek R., Abdrabou M.S., Werner B.S., Gohlke F. (2020). The implications of the glenoid angles and rotator cuff status in patients with osteoarthritis undergoing shoulder arthroplasty.. BMC Musculoskelet. Disord..

[128] Kapoor R., Husseini J.S., Staffa S.J., Palmer W.E., Torriani M., Chang C.Y., Joseph Simeone F. (2023). Posterior capsule edema in adhesive capsulitis: Comparison with established non-contrast MRI findings and multivariable analysis.. Eur. Radiol..

[129] Sasanuma H., Sugimoto H., Fujita A., Kanaya Y., Iijima Y., Saito T., Takeshita K. (2017). Characteristics of dynamic magnetic resonance imaging of idiopathic severe frozen shoulder.. J. Shoulder Elbow Surg..

[130] Sasanuma H., Sugimoto H., Iijima Y., Kanaya Y., Saito T., Takeshita K. (2018). Blood flow evaluation by dynamic magnetic resonance imaging of symptomatic rotator cuff tears and frozen shoulders.. J. Shoulder Elbow Surg..

[131] Lee J.S., Do J.G., Yoon K.J., Chae S.W., Park H.J., Park C.H., Lee Y.T. (2020). Voxel-based three-dimensional segmentation of the capsulo-synovium from contrast-enhanced MRI can represent clinical impairments in adhesive capsulitis.. Sci. Rep..

[132] Saito T., Sugimoto H., Sasanuma H., Iijima Y., Takeshita K. (2020). Characteristics of dynamic magnetic resonance imaging of symptomatic chronic calcifying tendinitis: Preliminary case reports.. JSES Int..

[133] Saito T., Sasanuma H., Iijima Y., Matsumura T., Takeshita K. (2021). Characteristics of post-traumatic shoulder stiffness on dynamic magnetic resonance imaging: Preliminary case reports.. JSES Rev. Rep. Tech..

[134] Iijima Y., Sugimoto H., Sasanuma H., Saito T., Kurashina W., Kanaya Y., Takeshita K. (2022). Reduction of abnormal blood flow in frozen shoulder after shoulder manipulation under ultrasound-guided cervical nerve root block: Semiquantitative analysis using dynamic magnetic resonance imaging.. JSES Int..

[135] Furukawa R., Morihara T., Arai Y., Ito H., Kida Y., Sukenari T., Horii M., Ikoma K., Fujiwara H., Kubo T. (2014). Diagnostic accuracy of magnetic resonance imaging for subscapularis tendon tears using radial-slice magnetic resonance images.. J. Shoulder Elbow Surg..

[136] Honda H., Morihara T., Arai Y., Horii M., Ito H., Furukawa R., Kida Y., Sukenari T., Ikoma K., Oda R., Yamada Y., Fujiwara H., Kubo T. (2015). Clinical application of radial magnetic resonance imaging for evaluation of rotator cuff tear.. Orthop. Traumatol. Surg. Res..

[137] Ogura A., Morihara T., Fujiwara H., Arai Y., Kida Y., Ito H., Furukawa R., Kabuto Y., Sukenari T., Kubo T. (2018). Validity of radial magnetic resonance imaging to determine the extent of Bankart lesions.. Clin. Imaging.

[138] Sasaki K., Morihara T., Kida Y., Furukawa R., Arai Y., Fujiwara H., Kubo T. (2018). Visualization of rotator cuff tear morphology by radial magnetic resonance imaging.. Clin. Imaging.

[139] Shibayama Y., Hirose T., Sugi A., Mizushima E., Watanabe Y., Tomii R., Iba K., Yamashita T. (2022). Relationship between preoperative size of rotator cuff tears measured using radial-slice magnetic resonance images and postoperative rotator cuff integrity: A prospective case-control study.. JSES Int..

[140] Gyftopoulos S., Beltran L.S., Gibbs K., Jazrawi L., Berman P., Babb J., Meislin R. (2016). Rotator cuff tear shape characterization: A comparison of two-dimensional imaging and three-dimensional magnetic resonance reconstructions.. J. Shoulder Elbow Surg..

[141] Schleich C., Bittersohl B., Antoch G., Krauspe R., Zilkens C., Kircher J. (2017). Thickness distribution of glenohumeral joint cartilage.. Cartilage.

[142] Khanna R., Saltzman M.D., Elliott J.M., Hoggarth M.A., Marra G.M., Omar I., Parrish T., Seitz A.L. (2019). Development of 3D method to assess intramuscular spatial distribution of fat infiltration in patients with rotator cuff tear: Reliability and concurrent validity.. BMC Musculoskelet. Disord..

[143] Wuennemann F., Kintzelé L., Zeifang F., Maier M.W., Burkholder I., Weber M.A., Kauczor H.U., Rehnitz C. (2019). Diagnostic performance of 3D-multi-Echo-data-image-combination (MEDIC) for evaluating SLAP lesions of the shoulder.. BMC Musculoskelet. Disord..

[144] Henninger H.B., Christensen G.V., Taylor C.E., Kawakami J., Hillyard B.S., Tashjian R.Z., Chalmers P.N. (2020). The muscle cross-sectional area on MRI of the shoulder can predict muscle volume: An MRI study in cadavers.. Clin. Orthop. Relat. Res..

[145] Wallenberg R.B., Belzer M.L., Ramsey D.C., Opel D.M., Berkson M.D., Gundle K.R., Nagy M.L., Boucher R.J., McCarron J.A. (2022). MRI-based 3-dimensional volumetric assessment of fatty infiltration and muscle atrophy in rotator cuff tears.. J. Shoulder Elbow Surg..

[146] Feuerriegel G.C., Kopp F.K., Pfeiffer D., Pogorzelski J., Wurm M., Leonhardt Y., Boehm C., Kronthaler S., Karampinos D.C., Neumann J., Schwaiger B.J., Makowski M.R., Woertler K., Gersing A.S. (2022). Evaluation of MR-derived simulated CT-like images and simulated radiographs compared to conventional radiography in patients with shoulder pain: A proof-of-concept study.. BMC Musculoskelet. Disord..

[147] Rosenthal Y., Samim M., Gyftopoulos S., Kolade O.O., Kwon Y.W., Zuckerman J.D., Virk M.S. (2022). 3D-MRI versus 3D-CT in the evaluation of glenoid deformity in glenohumeral arthritis using Dixon 3D FLASH sequence.. Skeletal Radiol..

[148] Lander S.T., Liles J.L., Kim B.I., Taylor D.C., Lau B.C. (2022). Comparison of computed tomography and 3D magnetic resonance imaging in evaluating glenohumeral instability bone loss.. J. Shoulder Elbow Surg..

[149] Kim B.I., Hudson C.P., Taylor D.C., Anakwenze O., Dickens J.F., Lau B.C. (2023). Distal clavicle autograft versus traditional and congruent arc latarjet procedures: A comparison of surface area and glenoid apposition with 3-dimensional computed tomography and 3-dimensional magnetic resonance imaging.. Am. J. Sports Med..

[150] Nagawa K., Hara Y., Shimizu H., Matsuura K., Inoue K., Kozawa E. (2024). 3-D sectional measurement approach for serial volume changes in shoulder muscles after arthroscopic rotator cuff repair.. Eur. J. Radiol. Open.

[151] Paul A.V., Udoh I., Bharadwaj A., Bokshan S., Owens B.D., Levine W.N., Garrigues G.E., Abrams J.S., McMahon P.J., Miniaci A., Nagda S., Braman J.P., MacDonald P., Riboh J.C., Kaar S., Lau B. (2024). Preoperative planning with three-dimensional CT *vs*. three-dimensional magnetic resonance imaging does not change surgical management for shoulder instability.. JSES Int..

[152] Khandare S., Jalics A., Lawrence R.L., Zauel R., Klochko C., Bey M.J. (2024). A novel 3D MRI-based approach for assessing supraspinatus muscle length.. J. Biomech..

[153] Haider S., Cabrera A., Thakur U., Xi Y., Chhabra A. (2024). Single-plane 3-dimensional isotropic spin-echo magnetic resonance imaging reconstructions of shoulder exhibit superior correlation to surgical findings than 2-dimensional dixon multiplanar magnetic resonance imaging.. J. Comput. Assist. Tomogr..

[154] Nozaki T., Tasaki A., Horiuchi S., Ochi J., Starkey J., Hara T., Saida Y., Yoshioka H. (2016). Predicting retear after repair of full-thickness rotator cuff tear: Two-point dixon MR imaging quantification of fatty muscle degeneration-initial experience with 1-year follow-up.. Radiology.

[155] Gilbert F., Böhm D., Eden L., Schmalzl J., Meffert R.H., Köstler H., Weng A.M., Ziegler D. (2016). Comparing the MRI-based Goutallier Classification to an experimental quantitative MR spectroscopic fat measurement of the supraspinatus muscle.. BMC Musculoskelet. Disord..

[156] Karampinos D.C., Holwein C., Buchmann S., Baum T., Ruschke S., Gersing A.S., Sutter R., Imhoff A.B., Rummeny E.J., Jungmann P.M. (2017). Proton density fat-fraction of rotator cuff muscles is associated with isometric strength 10 years after rotator cuff repair: A quantitative magnetic resonance imaging study of the shoulder.. Am. J. Sports Med..

[157] Kälin P.S., Huber F.A., Hamie Q.M., Issler L.S., Farshad-Amacker N.A., Ulbrich E.J., Guggenberger R. (2019). Quantitative MRI of visually intact rotator cuff muscles by multiecho Dixon-based fat quantification and diffusion tensor imaging.. J. Magn. Reson. Imaging.

[158] Wieser K., Joshy J., Filli L., Kriechling P., Sutter R., Fürnstahl P., Valdivieso P., Wyss S., Meyer D.C., Flück M., Gerber C. (2019). Changes of supraspinatus muscle volume and fat fraction after successful or failed arthroscopic rotator cuff repair.. Am. J. Sports Med..

[159] Lansdown D.A., Morrison C., Zaid M.B., Patel R., Zhang A.L., Allen C.R., Feeley B.T., Ma C.B. (2019). Preoperative IDEAL (Iterative Decomposition of Echoes of Asymmetrical Length) magnetic resonance imaging rotator cuff muscle fat fractions are associated with rotator cuff repair outcomes.. J. Shoulder Elbow Surg..

[160] Liu B., Xu J., Jin Y., Su W., Zhang X., Qiao Y., Yu W., Cheng L., Zhao J., Li Y. (2022). Advantages of 3–dimensional measurements for supraspinatus intramuscular fatty evaluation in patients with medium to massive rotator cuff tears: Comparison with a single sagittal slice.. Am. J. Sports Med..

[161] Addona J., Ahmed S.R., Almardawi R., Garcia Zapata L., Awan O.A., Davis D.L. (2023). Estimating 3D supraspinatus intramuscular fatty infiltration in older adults: A pilot study.. Acta Radiol..

[162] Matsuki K., Watanabe A., Ochiai S., Kenmoku T., Ochiai N., Obata T., Toyone T., Wada Y., Okubo T. (2014). Quantitative evaluation of fatty degeneration of the supraspinatus and infraspinatus muscles using T2 mapping.. J. Shoulder Elbow Surg..

[163] Iijima Y., Matsuki K., Hoshika S., Ueda Y., Onishi K., Tokai M., Takahashi N., Sugaya H., Dougo K., Watanabe A. (2017). Differences in fatty degeneration of rotator cuff muscles at different sites, as quantified by T2 mapping.. J. Orthop. Sci..

[164] Ganal E., Ho C.P., Wilson K.J., Surowiec R.K., Smith W.S., Dornan G.J., Millett P.J. (2016). Quantitative MRI characterization of arthroscopically verified supraspinatus pathology: Comparison of tendon tears, tendinosis and asymptomatic supraspinatus tendons with T2 mapping.. Knee Surg. Sports Traumatol. Arthrosc..

[165] Kang Y., Choi J.A. (2016). T2 mapping of articular cartilage of the glenohumeral joint at 3.0 T in healthy volunteers: A feasibility study.. Skeletal Radiol..

[166] Krepkin K., Bruno M., Raya J.G., Adler R.S., Gyftopoulos S. (2017). Quantitative assessment of the supraspinatus tendon on MRI using T2/T2* mapping and shear-wave ultrasound elastography: A pilot study.. Skeletal Radiol..

[167] Iijima Y., Matsuki K., Hoshika S., Ueda Y., Hamada H., Tokai M., Takahashi N., Sugaya H., Watanabe A. (2019). Relationship between postoperative retear and preoperative fatty degeneration in large and massive rotator cuff tears: Quantitative analysis using T2 mapping.. J. Shoulder Elbow Surg..

[168] Breighner R.E., Endo Y., Konin G.P., Gulotta L.V., Koff M.F., Potter H.G. (2018). Zero echo time imaging of the shoulder: Enhanced osseous detail by using MR imaging.. Radiology.

[169] Zhu Y., Cheng X., Ma Y., Wong J.H., Xie Y., Du J., Chang E.Y. (2018). Rotator cuff tendon assessment using magic-angle insensitive 3D ultrashort echo time cones magnetization transfer (UTE-Cones-MT) imaging and modeling with histological correlation.. J. Magn. Reson. Imaging.

[170] Ma Y.J., West J., Nazaran A., Cheng X., Hoenecke H., Du J., Chang E.Y. (2018). Feasibility of using an inversion-recovery ultrashort echo time (UTE) sequence for quantification of glenoid bone loss.. Skeletal Radiol.

[171] Guo T., Ma Y.J., High R.A., Tang Q., Wong J.H., Byra M., Searleman A.C., To S.C., Wan L., Le N., Du J., Chang E.Y. (2019). Assessment of an *in vitro* model of rotator cuff degeneration using quantitative magnetic resonance and ultrasound imaging with biochemical and histological correlation.. Eur. J. Radiol..

[172] Ashir A., Ma Y., Jerban S., Jang H., Wei Z., Le N., Du J., Chang E.Y. (2020). Rotator cuff tendon assessment in symptomatic and control groups using quantitative MRI.. J. Magn. Reson. Imaging.

[173] de Mello RAF (2020). Three-D zero echo time MRI versus 3-D CT for glenoid bone assessment.. Arthroscopy.

[174] Xie Y., Liu S., Qu J., Wu P., Tao H., Chen S. (2020). Quantitative magnetic resonance imaging UTE-T2* mapping of tendon healing after arthroscopic rotator cuff repair: A longitudinal study.. Am. J. Sports Med..

[175] Yıldız A.E., Yaraşır Y., Huri G., Aydıngöz Ü. (2022). Optimization of the grashey view radiograph for critical shoulder angle measurement: A reliability assessment with zero echo time MRI.. Orthop. J. Sports Med..

[176] Kenmoku T., Miyajima G., Tazawa R., Ishii D., Inoue K., Matsumoto M., Takaso M. (2023). Kinematic analysis of damaged capsulolabral structure in patients with anterior shoulder instability using cine-magnetic resonance imaging.. JSES Int..

[177] Sartoretti E., Sartoretti T., Binkert C., Najafi A., Schwenk Á., Hinnen M., van Smoorenburg L., Eichenberger B., Sartoretti-Schefer S. (2019). Reduction of procedure times in routine clinical practice with Compressed SENSE magnetic resonance imaging technique.. PLoS One.

[178] Gao F., Wen Z., Dou S., Kan X., Wei S., Ge Y. (2021). High-resolution simultaneous multi-slice accelerated turbo spin-echo musculoskeletal imaging: A head-to-head comparison with routine turbo spin-echo imaging.. Front. Physiol..

[179] Niitsu M., Saruya S., Sakaguchi K., Watarai K., Yoneyama M., Katsumata Y., Inoue K., Kozawa E. (2022). Motion-robust MR imaging of the shoulder using compressed SENSE MultiVane.. Eur. J. Radiol. Open.

[180] Hou B., Li Y., Xiong Y., Morelli J.N., Wang J., Liu C., Wu G., Li X. (2022). Comparison of CAIPIRINHA-accelerated 3D fat-saturated-SPACE MRI with 2D MRI sequences for the assessment of shoulder pathology.. Eur. Radiol..

[181] Kohli A., Pilkinton D.T., Xi Y., Cho G., Moore D., Mohammadi D., Chhabra A. (2022). Image quality improvement and motion degradation reduction in shoulder MR imaging: Comparison of BLADE and rectilinear techniques at 3-Tesla scanning.. Skeletal Radiol..

[182] Liu L., Wu G. (2023). Three-dimensional SPACE MR with CAIPIRINHA fourfold acceleration for assessing long head of biceps tendon.. Acta Radiol..

[183] Lazik-Palm A., Kraff O., Rietsch S.H.G., Ladd M.E., Kamminga M., Beck S., Quick H.H., Theysohn J.M. (2020). 7-T clinical MRI of the shoulder in patients with suspected lesions of the rotator cuff.. Eur. Radiol. Exp..

[184] Cantarelli Rodrigues T., Deniz C.M., Alaia E.F., Gorelik N., Babb J.S., Dublin J., Gyftopoulos S. (2020). Three-dimensional MRI bone models of the glenohumeral joint using deep learning: Evaluation of normal anatomy and glenoid bone loss.. Radiol. Artif. Intell..

[185] Medina G., Buckless C.G., Thomasson E., Oh L.S., Torriani M. (2021). Deep learning method for segmentation of rotator cuff muscles on MR images.. Skeletal Radiol..

[186] Conze P.H., Brochard S., Burdin V., Sheehan F.T., Pons C. (2020). Healthy versus pathological learning transferability in shoulder muscle MRI segmentation using deep convolutional encoder-decoders.. Comput. Med. Imaging Graph..

[187] Wang G., Han Y. (2021). Convolutional neural network for automatically segmenting magnetic resonance images of the shoulder joint.. Comput. Methods Programs Biomed..

[188] Mu X., Cui Y., Bian R., Long L., Zhang D., Wang H., Shen Y., Wu J., Zou G. (2021). In-depth learning of automatic segmentation of shoulder joint magnetic resonance images based on convolutional neural networks.. Comput. Methods Programs Biomed..

[189] Kim H., Shin K., Kim H., Lee E., Chung S.W., Koh K.H., Kim N. (2022). Can deep learning reduce the time and effort required for manual segmentation in 3D reconstruction of MRI in rotator cuff tears?. PLoS One.

[190] Lee S.H., Lee J., Oh K.S., Yoon J.P., Seo A., Jeong Y., Chung S.W. (2023). Automated 3-dimensional MRI segmentation for the posterosuperior rotator cuff tear lesion using deep learning algorithm.. PLoS One.

[191] Hess H., Ruckli A.C., Bürki F., Gerber N., Menzemer J., Burger J., Schär M., Zumstein M.A., Gerber K. (2023). Deep-learning-based segmentation of the shoulder from MRI with inference accuracy prediction.. Diagnostics.

[192] Wong V., Calivá F., Su F., Pedoia V., Lansdown D. (2023). Comparing bone shape models from deep learning processing of magnetic resonance imaging to computed tomography-based models.. JSES Int..

[193] Sezer A. (2023). Mask region-based convolutional neural network segmentation of the humerus and scapula from proton density-weighted axial shoulder magnetic resonance images.. Jt. Dis. Relat. Surg..

[194] Alipour E., Chalian M., Pooyan A., Azhideh A., Shomal Zadeh F., Jahanian H. (2024). Automatic MRI–based rotator cuff muscle segmentation using U-Nets.. Skeletal Radiol..

[195] Sezer A., Sezer H.B. (2019). Capsule network-based classification of rotator cuff pathologies from MRI.. Comput. Electr. Eng..

[196] Kim J.Y., Ro K., You S., Nam B.R., Yook S., Park H.S., Yoo J.C., Park E., Cho K., Cho B.H., Kim I.Y. (2019). Development of an automatic muscle atrophy measuring algorithm to calculate the ratio of supraspinatus in supraspinous fossa using deep learning.. Comput. Methods Programs Biomed..

[197] Shim E., Kim J.Y., Yoon J.P., Ki S.Y., Lho T., Kim Y., Chung S.W. (2020). Automated rotator cuff tear classification using 3D convolutional neural network.. Sci. Rep..

[198] Ro K., Kim J.Y., Park H., Cho B.H., Kim I.Y., Shim S.B., Choi I.Y., Yoo J.C. (2021). Deep-learning framework and computer assisted fatty infiltration analysis for the supraspinatus muscle in MRI.. Sci. Rep..

[199] Key S., Demir S., Gurger M., Yilmaz E., Barua P.D., Dogan S., Tuncer T., Arunkumar N., Tan R.S., Acharya U.R. (2022). ViVGG19: Novel exemplar deep feature extraction-based shoulder rotator cuff tear and biceps tendinosis detection using magnetic resonance images.. Med. Eng. Phys..

[200] Yao J., Chepelev L., Nisha Y., Sathiadoss P., Rybicki F.J., Sheikh A.M. (2022). Evaluation of a deep learning method for the automated detection of supraspinatus tears on MRI.. Skeletal Radiol..

[201] Saavedra J.P., Droppelmann G., García N., Jorquera C., Feijoo F. (2023). High-accuracy detection of supraspinatus fatty infiltration in shoulder MRI using convolutional neural network algorithms.. Front. Med..

[202] Ni M., Gao L., Chen W., Zhao Q., Zhao Y., Jiang C., Yuan H. (2024). Preliminary exploration of deep learning-assisted recognition of superior labrum anterior and posterior lesions in shoulder MR arthrography.. Int. Orthop..

[203] Lee K.C., Cho Y., Ahn K.S., Park H.J., Kang Y.S., Lee S., Kim D., Kang C.H. (2023). Deep-learning-based automated rotator cuff tear screening in three planes of shoulder MRI.. Diagnostics.

[204] Guo D., Liu X., Wang D., Tang X., Qin Y. (2023). Development and clinical validation of deep learning for auto-diagnosis of supraspinatus tears.. J. Orthop. Surg. Res..

[205] Esfandiari M.A., Fallah Tafti M., Jafarnia Dabanloo N., Yousefirizi F. (2023). Detection of the rotator cuff tears using a novel convolutional neural network from magnetic resonance image (MRI).. Heliyon.

[206] Lin D.J., Schwier M., Geiger B., Raithel E., von Busch H., Fritz J., Kline M., Brooks M., Dunham K., Shukla M., Alaia E.F., Samim M., Joshi V., Walter W.R., Ellermann J.M., Ilaslan H., Rubin D., Winalski C.S., Recht M.P. (2023). Deep learning diagnosis and classification of rotator cuff tears on shoulder MRI.. Invest. Radiol..

[207] Oeding J.F., Pareek A., Nieboer M.J., Rhodes N.G., Tiegs-Heiden C.A., Camp C.L., Martin R.K., Moatshe G., Engebretsen L., Sanchez-Sotelo J. (2024). A machine learning model demonstrates excellent performance in predicting subscapularis tears based on pre-operative imaging parameters alone.. Arthroscopy.

[208] Fei Y., Wan Y., Xu L., Huang Z., Ruan D., Wang C., He P., Zhou X., Heng B.C., Niu T., Shen W., Wu Y. (2024). Novel methods to diagnose rotator cuff tear and predict post-operative Re-tear: Radiomics models.. Asia. Pac. J. Sports Med. Arthrosc. Rehabil. Technol..

[209] Zhang Z., Ke C., Zhang Z., Chen Y., Weng H., Dong J., Hao M., Liu B., Zheng M., Li J., Ding S., Dong Y., Peng Z. (2024). Re-tear after arthroscopic rotator cuff repair can be predicted using deep learning algorithm.. Front. Artif. Intell..

[210] Koch K.M., Sherafati M., Arpinar V.E., Bhave S., Ausman R., Nencka A.S., Lebel R.M., McKinnon G., Kaushik S.S., Vierck D., Stetz M.R., Fernando S., Mannem R. (2021). Analysis and evaluation of a deep learning reconstruction approach with denoising for orthopedic MRI.. Radiol. Artif. Intell..

[211] Kaniewska M., Deininger-Czermak E., Getzmann J.M., Wang X., Lohezic M., Guggenberger R. (2022). Application of deep learning–based image reconstruction in MR imaging of the shoulder joint to improve image quality and reduce scan time.. Eur. Radiol..

[212] Obama Y., Ohno Y., Yamamoto K., Ikedo M., Yui M., Hanamatsu S., Ueda T., Ikeda H., Murayama K., Toyama H. (2022). MR imaging for shoulder diseases: Effect of compressed sensing and deep learning reconstruction on examination time and imaging quality compared with that of parallel imaging.. Magn. Reson. Imaging.

[213] Hahn S., Yi J., Lee H.J., Lee Y., Lim Y.J., Bang J.Y., Kim H., Lee J. (2022). Image quality and diagnostic performance of accelerated shoulder MRI with deep learning–based reconstruction.. AJR Am. J. Roentgenol..

[214] Dratsch T., Siedek F., Zäske C., Sonnabend K., Rauen P., Terzis R., Hahnfeldt R., Maintz D., Persigehl T., Bratke G., Iuga A. (2023). Reconstruction of shoulder MRI using deep learning and compressed sensing: A validation study on healthy volunteers.. Eur. Radiol. Exp..

[215] Feuerriegel G.C., Weiss K., Kronthaler S., Leonhardt Y., Neumann J., Wurm M., Lenhart N.S., Makowski M.R., Schwaiger B.J., Woertler K., Karampinos D.C., Gersing A.S. (2023). Evaluation of a deep learning-based reconstruction method for denoising and image enhancement of shoulder MRI in patients with shoulder pain.. Eur. Radiol..

[216] Hahn S., Yi J., Lee H.J., Lee Y., Lee J., Wang X., Fung M. (2023). Comparison of deep learning-based reconstruction of PROPELLER Shoulder MRI with conventional reconstruction.. Skeletal Radiol..

[217] Shiraishi K., Nakaura T., Uetani H., Nagayama Y., Kidoh M., Kobayashi N., Morita K., Yamahita Y., Miyamoto T., Hirai T. (2023). Combination use of compressed sensing and deep learning for shoulder magnetic resonance imaging with various sequences.. J. Comput. Assist. Tomogr..

[218] Liu Z., Wen B., Wang Z., Wang K., Xie L., Kang Y., Tao Q., Wang W., Zhang Y., Cheng J., Zhang Y. (2024). Deep learning-based reconstruction enhances image quality and improves diagnosis in magnetic resonance imaging of the shoulder joint.. Quant. Imaging Med. Surg..

[219] Feuerriegel G.C., Weiss K., Tu Van A., Leonhardt Y., Neumann J., Gassert F.T., Haas Y., Schwarz M., Makowski M.R., Woertler K., Karampinos D.C., Gersing A.S. (2024). Deep-learning-based image quality enhancement of CT-like MR imaging in patients with suspected traumatic shoulder injury.. Eur. J. Radiol..

[220] Herrmann J., Feng Y.S., Gassenmaier S., Grunz J.P., Koerzdoerfer G., Lingg A., Almansour H., Nickel D., Othman A.E., Afat S. (2024). Fast 5-minute shoulder MRI protocol with accelerated TSE-sequences and deep learning image reconstruction for the assessment of shoulder pain at 1.5 and 3 Tesla.. Eur. J. Radiol. Open.

[221] Xie Y., Tao H., Li X., Hu Y., Liu C., Zhou B., Cai J., Nickel D., Fu C., Xiong B., Chen S. (2024). Prospective comparison of standard and deep learning–reconstructed turbo spin-echo MRI of the shoulder.. Radiology.

[222] Kim M., Kim J.Y., Kim S.H., Van Hoeke S., De Neve W., Kim M. (2020). MRI-based diagnosis of rotator cuff tears using deep learning and weighted linear combinations.. Proc. Mach. Learn. Res..

[223] Bélanger V., Dupuis F., Leblond J., Roy J. (2019). Accuracy of examination of the long head of the biceps tendon in the clinical setting: A systematic review.. J. Rehabil. Med..

[224] Lädermann A., Collin P., Zbinden O., Meynard T., Saffarini M., Chiu J.C.H. (2021). Diagnostic accuracy of clinical tests for subscapularis tears: A systematic review and meta-analysis.. Orthop. J. Sports Med..

[225] Lizzio V.A., Meta F., Fidai M., Makhni E.C. (2017). Clinical evaluation and physical exam findings in patients with anterior shoulder instability.. Curr. Rev. Musculoskelet. Med..

[226] Balevi Batur E., Bekin Sarıkaya P.Z., Kaygısız M.E., Albayrak Gezer I., Levendoglu F. (2022). Diagnostic dilemma: Which clinical tests are most accurate for diagnosing supraspinatus muscle tears and tendinosis when compared to magnetic resonance imaging?. Cureus.

[227] Hanchard N.C.A., Lenza M., Handoll H.H.G., Takwoingi Y. (2013). Physical tests for shoulder impingements and local lesions of bursa, tendon or labrum that may accompany impingement.. Cochrane Libr..

[228] Sasiponganan C., Dessouky R., Ashikyan O., Pezeshk P., McCrum C., Xi Y., Chhabra A. (2019). Subacromial impingement anatomy and its association with rotator cuff pathology in women: Radiograph and MRI correlation, a retrospective evaluation.. Skeletal Radiol..

[229] Pandey V., Vijayan D., Tapashetti S., Agarwal L., Kamath A., Acharya K., Maddukuri S., Willems W.J. (2016). Does scapular morphology affect the integrity of the rotator cuff?. J. Shoulder Elbow Surg..

[230] Balke M., Schmidt C., Dedy N., Banerjee M., Bouillon B., Liem D. (2013). Correlation of acromial morphology with impingement syndrome and rotator cuff tears.. Acta Orthop..

[231] Chalmers P.N., Beck L., Miller M., Kawakami J., Dukas A.G., Burks R.T., Greis P.E., Tashjian R.Z. (2020). Acromial morphology is not associated with rotator cuff tearing or repair healing.. J. Shoulder Elbow Surg..

[232] Klatte-Schulz F., Thiele K., Scheibel M., Duda G.N., Wildemann B. (2022). Subacromial bursa: A neglected tissue is gaining more and more attention in clinical and experimental research.. Cells.

[233] De Filippo M., Schirò S., Sarohia D., Barile A., Saba L., Cella S., Castagna A. (2020). Imaging of shoulder instability.. Skeletal Radiol..

[234] Aydingöz Ü., Canbulat N., Demirhan M. (2014). Omuz bölgesinin radyolojik deǧerlendirmesi.. Turk. Fizik. Rehabil. Derg..

[235] Sanders T.G., Jersey S.L. (2005). Conventional radiography of the shoulder.. Semin. Roentgenol..

[236] Bao M.H., DeAngelis J.P., Wu J.S. (2022). Imaging of traumatic shoulder injuries – Understanding the surgeon’s perspective.. Eur. J. Radiol. Open.

[237] Weaver J., Omar I., Chadwick N., Shechtel J., Elifritz J., Shultz C., Taljanovic M. (2023). Update on shoulder arthroplasties with emphasis on imaging.. J. Clin. Med..

[238] Sivagurunathan G., Shirodkar K., Hegde G., Shamshuddin S., Proctor R., Naqvi J., Knowles D., Ali I. (2023). Musculoskeletal computed tomography: How to add value when reporting adult upper limb trauma.. J. Comput. Assist. Tomogr..

[239] Singh A., Thukral C.L., Gupta K., Singh M.I., Lata S., Arora R.K. (2017). Role and correlation of high resolution ultrasound and magnetic resonance imaging in evaluation of patients with shoulder pain.. Pol. J. Radiol..

[240] Zoga A.C., Kamel S.I., Hynes J.P., Kavanagh E.C., O’Connor P.J., Forster B.B. (2021). The evolving roles of MRI and ultrasound in first-line imaging of rotator cuff injuries.. AJR Am. J. Roentgenol..

[241] Saraya S., El Bakry R. (2016). Ultrasound: Can it replace MRI in the evaluation of the rotator cuff tears?. Egypt. J. Radiol. Nucl. Med..

[242] Roy J.S., Braën C., Leblond J., Desmeules F., Dionne C.E., MacDermid J.C., Bureau N.J., Frémont P. (2015). Diagnostic accuracy of ultrasonography, MRI and MR arthrography in the characterisation of rotator cuff disorders: A systematic review and meta-analysis.. Br. J. Sports Med..

[243] Shen P.C., Lin T.Y., Wu W.T., Özçakar L., Chang K.V. (2024). Comparison of ultrasound- *vs*. landmark-guided injections for musculoskeletal pain: An umbrella review.. J. Rehabil. Med..

[244] Tortora S., Messina C., Gitto S., Chianca V., Serpi F., Gambino A., Pedone L., Carrafiello G., Sconfienza L.M., Albano D. (2021). Ultrasound-guided musculoskeletal interventional procedures around the shoulder.. J. Ultrason..

[245] Moor B.K., Bouaicha S., Rothenfluh D.A., Sukthankar A., Gerber C. (2013). Is there an association between the individual anatomy of the scapula and the development of rotator cuff tears or osteoarthritis of the glenohumeral joint?: A radiological study of the critical shoulder angle.. Bone Joint J..

[246] Gumina S., Polizzotti G., Spagnoli A., Carbone S., Candela V. (2022). Critical shoulder angle (CSA): Age and gender distribution in the general population.. J. Orthop. Traumatol..

[247] Bigliani L.U., Ticker J.B., Flatow E.L., Soslotcsky L.J., Mow V.C. (1991). The relationship of acromial architecture to rotator cuff disease.. Clin. Sports Med..

[248] Liu J., Dai S., Deng H., Qiu D., Liu L., Li M., Chen Z., Kang J., Tao J. (2023). Evaluation of the prognostic value of the anatomical characteristics of the bony structures in the shoulder in bursal-sided partial-thickness rotator cuff tears.. Front. Public Health.

[249] Liu C.T., Miao J.Q., Wang H., an Ge H., Wang X.H., Cheng B. (2021). The association between acromial anatomy and articular-sided partial thickness of rotator cuff tears.. BMC Musculoskelet. Disord..

[250] Kim J.M., Kim Y.W., Kim H.S., Lee S.C., Chun Y.M., Joo S.H., Lim H.S. (2019). The relationship between rotator cuff tear and four acromion types: Cross-sectional study based on shoulder magnetic resonance imaging in 227 patients.. Acta Radiol..

[251] Ilyas G., Senyuva G., Ipci F.B. (2023). Evaluation of preoperative magnetic resonance imaging parameters with arthroscopic validation of subscapularis tendon abnormalities in 187 patients at a single center in Turkey.. Med. Sci. Monit..

[252] Cetinkaya M., Öner A.Y., Ataoglu M.B., Ozer M., Ayanoglu T., Kanatli U. (2017). Lesser tuberosity cysts and their relationship with subscapularis tears and subcoracoid impingement.. J. Orthop. Sci..

[253] nair A., Rao S.N., Kumaran C.K., Kochukunju B.V. (2016). Clinico-radiological correlation of subcoracoid impingement with reduced coracohumeral interval and its relation to subscapularis tears in Indian patients.. J. Clin. Diagn. Res..

[254] Asal N., Şahan M.H. (2018). Radiological variabilities in subcoracoid impingement: Coracoid morphology, coracohumeral distance, coracoglenoid angle, and coracohumeral angle.. Med. Sci. Monit..

[255] Lee R.K.L., Griffith J.F., Tong M.M.P., Sharma N., Yung P. (2013). Glenoid bone loss: Assessment with MR imaging.. Radiology.

[256] Gyftopoulos S., Hasan S., Bencardino J., Mayo J., Nayyar S., Babb J., Jazrawi L. (2012). Diagnostic accuracy of MRI in the measurement of glenoid bone loss.. AJR Am. J. Roentgenol..

[257] Weber A.E., Bolia I.K., Horn A., Villacis D., Omid R., Tibone J.E. (2021). Glenoid bone loss in shoulder instability: Superiority of 3-D computed tomography over two-dimensional magnetic resonance imaging using established methodology.. Clin Orthop Surg.

[258] Yamamoto N., Itoi E., Abe H., Minagawa H., Seki N., Shimada Y., Okada K. (2007). Contact between the glenoid and the humeral head in abduction, external rotation, and horizontal extension: A new concept of glenoid track.. J. Shoulder Elbow Surg..

[259] Probyn L.J., White L.M., Salonen D.C., Tomlinson G., Boynton E.L. (2007). Recurrent symptoms after shoulder instability repair: Direct MR arthrographic assessment--correlation with second-look surgical evaluation.. Radiology.

[260] Di Giacomo G., Itoi E., Burkhart S.S. (2014). Evolving concept of bipolar bone loss and the Hill-Sachs lesion: From “engaging/non-engaging” lesion to “on-track/off-track” lesion.. Arthroscopy.

[261] Gyftopoulos S., Beltran L.S., Bookman J., Rokito A. (2015). MRI evaluation of bipolar bone loss using the on-track off-track method: A feasibility study.. AJR Am. J. Roentgenol..

[262] Gyftopoulos S., Yemin A., Bencardino J., Babb J., Bencardino J. (2013). Engaging Hill-Sachs lesion: Is there an association between this lesion and findings on MRI?. AJR Am. J. Roentgenol..

[263] Li R.T., Kane G., Drummond M., Golan E., Wilson K., Lesniak B.P., Rodosky M., Lin A. (2021). On-track lesions with a small distance to dislocation are associated with failure after arthroscopic anterior shoulder stabilization.. J. Bone Joint Surg. Am..

[264] Friedman L.G.M., Ulloa S.A., Braun D.T., Saad H.A., Jones M.H., Miniaci A.A. (2014). Glenoid bone loss measurement in recurrent shoulder dislocation: Assessment of measurement agreement between CT and MRI.. Orthop. J. Sports Med..

[265] Ahmad R.G. (2022). Shoulder impingement: Various risk factors for supraspinatus tendon tear.. Medicine.

[266] Siow M.Y., Mitchell B.C., Hachadorian M., Wang W., Bastrom T., Kent W.T., Huang B.K., Edmonds E.W. (2021). Association between rotator cuff tears and superior migration of the humeral head: An MRI-based anatomic study.. Orthop. J. Sports Med..

[267] Tamura T., Tokunaga T., Karasugi T., Miyamoto T., Kikukawa K. (2023). The remaining teres minor and subscapularis may contribute to preventing superior migration of the humeral head and progression of osteoarthritic change in rotator cuff tears.. JSES Int..

[268] Garg A.K., Meena A., Farinelli L., D’Ambrosi R., Tapasvi S., Braun S. (2024). Partial subscapularis tear: State-of-the-art.. J. ISAKOS.

[269] Kikukawa K., Ide J., Kikuchi K., Morita M., Mizuta H., Ogata H. (2014). Hypertrophic changes of the teres minor muscle in rotator cuff tears: Quantitative evaluation by magnetic resonance imaging.. J. Shoulder Elbow Surg..

[270] Almeida G.G., Graf N., Wildermuth S., Fischer T., Waelti S., Jacxsens M., Leschka S., Dietrich T.J. (2023). Diagnostic performance of long head of biceps tendon tears on MRI: Systematic review and meta-analysis.. Eur. Radiol..

[271] Dong W., Du K., Shi B., Wang T., Lu B., Hou Z., Zhang Y., Guo J. (2024). Distribution and analysis of subacromial spurs and the relationship with acromial classification and angle in healthy individuals.. PLoS One.

[272] Ahn K.S., Kang C.H., Kim Y., Jeong W.K. (2015). Diagnosis of adhesive capsulitis: Comparison of contrast-enhanced MRI with noncontrast-enhanced MRI.. Clin. Imaging.

[273] Major N.M., Browne J., Domzalski T., Cothran R.L., Helms C.A. (2011). Evaluation of the glenoid labrum with 3-T MRI: Is intraarticular contrast necessary?. AJR Am. J. Roentgenol..

[274] Tamai K., Mashitori H., Ohno W., Hamada J., Sakai H., Saotome K. (2004). Synovial response to intraarticular injections of hyaluronate in frozen shoulder: A quantitative assessment with dynamic magnetic resonance imaging.. J. Orthop. Sci..

[275] Costa C., Incio J., Soares R. (2007). Angiogenesis and chronic inflammation: Cause or consequence?. Angiogenesis.

[276] Okuno Y., Yasumoto T., Koganemaru M., Suyama Y., Nishiofuku H., Horikawa M., Komemushi A. (2022). Transarterial embolization of neovascularity for refractory nighttime shoulder pain: A multicenter, open-label, feasibility trial.. J. Vasc. Interv. Radiol..

[277] Shintaku T., Inui S., Ikegami H., Yoshizawa S., Ishii H., Sakamoto M., Musha Y., Okuno Y., Kuji I. (2023). Alteration of chronic inflammatory status by transarterial embolization in frozen shoulder evaluated by fluorine-18 fluorodeoxyglucose positron-emission tomography/computed tomography.. J. Shoulder Elbow Surg..

[278] Aly M., Fashina C., Hagiga A., Hafez A., Di Mascio L. (2023). Transcatheter arterial embolisation for the relief of shoulder and elbow chronic joint pain refractory to conventional treatment: Systematic review.. J. Med. Imaging Radiat. Oncol..

[279] Mohtadi N.G., Vellet A.D., Clark M.L., Hollinshead R.M., Sasyniuk T.M., Fick G.H., Burton P.J. (2004). A prospective, double-blind comparison of magnetic resonance imaging and arthroscopy in the evaluation of patients presenting with shoulder pain.. J. Shoulder Elbow Surg..

[280] Gyftopoulos S., Beltran L.S., Yemin A., Strauss E., Meislin R., Jazrawi L., Recht M.P. (2014). Use of 3D MR reconstructions in the evaluation of glenoid bone loss: A clinical study.. Skeletal Radiol..

[281] Nozaki T., Tasaki A., Horiuchi S., Osakabe C., Ohde S., Saida Y., Yoshioka H. (2015). Quantification of fatty degeneration within the supraspinatus muscle by using a 2-point dixon method on 3-T MRI.. AJR Am. J. Roentgenol..

[282] Bureau N.J., Deslauriers M., Lepage-Saucier M., Rouleau D.M., Roy A., Tétreault P., Hagemeister N. (2018). Rotator cuff tear morphologic parameters at magnetic resonance imaging: Relationship with muscle atrophy and fatty infiltration and patient-reported function and health-related quality of life.. J. Comput. Assist. Tomogr..

[283] Goutallier D., Postel J.M., Bernageau J., Lavau L., Voisin M.C. (1994). Fatty muscle degeneration in cuff ruptures. Pre- and postoperative evaluation by CT scan.. Clin. Orthop. Relat. Res..

[284] Cheung S., Dillon E., Tham S.C., Feeley B.T., Link T.M., Steinbach L., Ma C.B. (2011). The presence of fatty infiltration in the infraspinatus: Its relation with the condition of the supraspinatus tendon.. Arthroscopy.

[285] Kellman P., Hernando D., Shah S., Zuehlsdorff S., Jerecic R., Mancini C., Liang Z.P., Arai A.E. (2009). Multiecho dixon fat and water separation method for detecting fibrofatty infiltration in the myocardium.. Magn. Reson. Med..

[286] Oak N.R., Gumucio J.P., Flood M.D., Saripalli A.L., Davis M.E., Harning J.A., Lynch E.B., Roche S.M., Bedi A., Mendias C.L. (2014). Inhibition of 5-LOX, COX-1, and COX-2 increases tendon healing and reduces muscle fibrosis and lipid accumulation after rotator cuff repair.. Am. J. Sports Med..

[287] Lee S., Lucas R.M., Lansdown D.A., Nardo L., Lai A., Link T.M., Krug R., Ma C.B. (2015). Magnetic resonance rotator cuff fat fraction and its relationship with tendon tear severity and subject characteristics.. J. Shoulder Elbow Surg..

[288] Kuzel B.R., Grindel S., Papandrea R., Ziegler D. (2013). Fatty infiltration and rotator cuff atrophy.. J. Am. Acad. Orthop. Surg..

[289] Nardo L., Karampinos D.C., Lansdown D.A., Carballido-Gamio J., Lee S., Maroldi R., Ma C.B., Link T.M., Krug R. (2014). Quantitative assessment of fat infiltration in the rotator cuff muscles using water-fat MRI.. J. Magn. Reson. Imaging.

[290] Dardzinski B.J., Mosher T.J., Li S., Van Slyke M.A., Smith M.B. (1997). Spatial variation of T2 in human articular cartilage.. Radiology.

[291] Mosher T.J., Dardzinski B.J. (2004). Cartilage MRI T2 relaxation time mapping: Overview and applications.. Semin. Musculoskelet. Radiol..

[292] Choi H.S., Lee B.I., Kim J.H., Cho H.K., Seo G.W. (2021). A technique for repairing rotator cuff transtendinous tears with a remnant attached to the footprint.. J. Orthop. Surg. Res..

[293] Kim Y.K., Jung K.H., Kim J.W., Kim U.S., Hwang D.H. (2018). Factors affecting rotator cuff integrity after arthroscopic repair for medium-sized or larger cuff tears: A retrospective cohort study.. J. Shoulder Elbow Surg..

[294] Kim I.B., Kim M.W. (2016). Risk factors for retear after arthroscopic repair of full-thickness rotator cuff tears using the suture bridge technique: Classification system.. Arthroscopy.

[295] Lee K.W., Seo D.W., Bae K.W., Choy W.S. (2013). Clinical and radiological evaluation after arthroscopic rotator cuff repair using suture bridge technique.. Clin. Orthop. Surg..

[296] Kenn W., Böhm D., Gohlke F., Hümmer C., Köstler H., Hahn D. (2004). 2D SPLASH: A new method to determine the fatty infiltration of the rotator cuff muscles.. Eur. Radiol..

[297] Pfirrmann C.W.A., Schmid M.R., Zanetti M., Jost B., Gerber C., Hodler J. (2004). Assessment of fat content in supraspinatus muscle with proton MR spectroscopy in asymptomatic volunteers and patients with supraspinatus tendon lesions.. Radiology.

[298] van de Sande M.A.J., Stoel B.C., Obermann W.R., Tjong a Lieng J.G.S., Rozing P.M. (2005). Quantitative assessment of fatty degeneration in rotator cuff muscles determined with computed tomography.. Invest. Radiol..

[299] Robertson P.L., Schweitzer M.E., Mitchell D.G., Schlesinger F., Epstein R.E., Frieman B.G., Fenlin J.M. (1995). Rotator cuff disorders: Interobserver and intraobserver variation in diagnosis with MR imaging.. Radiology.

[300] Puel U., Lombard C., Hossu G., Louis M., Blum A., Teixeira P.A.G., Gillet R. (2023). Zero echo time MRI in shoulder MRI protocols for the diagnosis of rotator cuff calcific tendinopathy improves identification of calcific deposits compared to conventional MR sequences but remains sub-optimal compared to radiographs.. Eur. Radiol..

[301] Lippitt S., Matsen F. (1993). Mechanisms of glenohumeral joint stability.. Clin. Orthop. Relat. Res..

[302] Friedman R.J., Bonutti P.M., Genez B. (1998). Cine magnetic resonance imaging of the subcoracoid region.. Orthopedics.

[303] Allmann K.H., Uhl M., Gufler H., Biebow N., Hauer M.P., Kotter E., Reichelt A., Langer M. (1997). Cine-mr imaging of the shoulder.. Acta Radiol..

[304] Bonutti P.M., Norfray J.F., Friedman R.J., Genez B.M. (1993). Kinematic MRI of the shoulder.. J. Comput. Assist. Tomogr..

[305] Hatta T., Yamamoto N., Sano H., Omori Y., Sugamoto K., Suzuki K., Itoi E. (2017). Three-dimensional morphometric analysis of the coracohumeral distance using magnetic resonance imaging.. Orthop. Rev..

[306] Pierrart J., Lefèvre-Colau M.M., Skalli W., Vuillemin V., Masmejean E.H., Cuénod C.A., Gregory T.M. (2014). New dynamic three-dimensional MRI technique for shoulder kinematic analysis.. J. Magn. Reson. Imaging.

[307] Adams G.R., Duvoisin M.R., Dudley G.A. (1992). Magnetic resonance imaging and electromyography as indexes of muscle function.. J. Appl. Physiol..

[308] Shellock F.G., Fukunaga T., Mink J.H., Edgerton V.R. (1991). Acute effects of exercise on MR imaging of skeletal muscle: Concentric vs eccentric actions.. AJR Am. J. Roentgenol..

[309] Meyer R.A., Prior B.M. (2000). Functional magnetic resonance imaging of muscle.. Exerc. Sport Sci. Rev..

[310] Ploutz-Snyder L.L., Yackel-Giamis E.L., Rosenbaum A.E., Formikell M. (2000). Use of muscle functional magnetic resonance imaging with older individuals.. J. Gerontol. A Biol. Sci. Med. Sci..

[311] Dickx N., Cagnie B., Achten E., Vandemaele P., Parlevliet T., Danneels L. (2008). Changes in lumbar muscle activity because of induced muscle pain evaluated by muscle functional magnetic resonance imaging.. Spine.

[312] Disler D.G., Cohen M.S., Krebs D.E., Roy S.H., Rosenthal D.I. (1995). Dynamic evaluation of exercising leg muscle in healthy subjects with echo planar MR imaging: Work rate and total work determine rate of T2 change.. J. Magn. Reson. Imaging.

[313] Yue G., Alexander A.L., Laidlaw D.H., Gmitro A.F., Unger E.C., Enoka R.M. (1994). Sensitivity of muscle proton spin-spin relaxation time as an index of muscle activation.. J. Appl. Physiol..

[314] Takeda Y., Kashiwaguchi S., Endo K., Matsuura T., Sasa T. (2002). The most effective exercise for strengthening the supraspinatus muscle: Evaluation by magnetic resonance imaging.. Am. J. Sports Med..

[315] Rosenkrantz A.B., Mannelli L., Mossa D., Babb J.S. (2011). Breath-hold T2-weighted MRI of the liver at 3 T using the BLADE technique: Impact upon image quality and lesion detection.. Clin. Radiol..

[316] Pipe J.G. (1999). Motion correction with PROPELLER MRI: Application to head motion and free-breathing cardiac imaging.. Magn. Reson. Med..

[317] Mavroidis P., Giankou E., Tsikrika A., Kapsalaki E., Chatzigeorgiou V., Batsikas G., Zaimis G., Kostopoulos S., Glotsos D., Ninos K., Georgountzos V., Kavouras D., Lavdas E. (2017). Brain imaging: Comparison of T1W FLAIR BLADE with conventional T1W SE.. Magn. Reson. Imaging.

[318] Fritz J., Guggenberger R., Grande F.D. (2021). Rapid Musculoskeletal MRI in 2021: Clinical Application of advanced accelerated techniques.. AJR Am. J. Roentgenol..

[319] Breuer F.A., Blaimer M., Heidemann R.M., Mueller M.F., Griswold M.A., Jakob P.M. (2005). Controlled aliasing in parallel imaging results in higher acceleration (CAIPIRINHA) for multi-slice imaging.. Magn. Reson. Med..

[320] Knoll F., Murrell T., Sriram A., Yakubova N., Zbontar J., Rabbat M., Defazio A., Muckley M.J., Sodickson D.K., Zitnick C.L., Recht M.P. (2020). Advancing machine learning for MR image reconstruction with an open competition: Overview of the 2019 fastMRI challenge.. Magn. Reson. Med..

[321] Lebel RM (2008). Performance characterization of a novel deep learning-based MR image reconstruction pipeline.. arXiv.

[322] Kim M., Kim H.S., Park J.E., Park S.Y., Kim Y.H., Kim S.J., Lee J., Lebel M.R. (2022). Thin-slice pituitary MRI with deep learning–based reconstruction for preoperative prediction of cavernous sinus invasion by pituitary adenoma: A prospective study.. AJNR Am. J. Neuroradiol..

[323] Subhas N., Li H., Yang M., Winalski C.S., Polster J., Obuchowski N., Mamoto K., Liu R., Zhang C., Huang P., Gaire S.K., Liang D., Shen B., Li X., Ying L. (2020). Diagnostic interchangeability of deep convolutional neural networks reconstructed knee MR images: Preliminary experience.. Quant. Imaging Med. Surg..

[324] Recht M.P., Zbontar J., Sodickson D.K., Knoll F., Yakubova N., Sriram A., Murrell T., Defazio A., Rabbat M., Rybak L., Kline M., Ciavarra G., Alaia E.F., Samim M., Walter W.R., Lin D.J., Lui Y.W., Muckley M., Huang Z., Johnson P., Stern R., Zitnick C.L. (2020). Using deep learning to accelerate knee MRI at 3 T: Results of an interchangeability study.. AJR Am. J. Roentgenol..

[325] Almansour H., Herrmann J., Gassenmaier S., Afat S., Jacoby J., Koerzdoerfer G., Nickel D., Mostapha M., Nadar M., Othman A.E. (2023). Deep learning reconstruction for accelerated spine MRI: Prospective analysis of interchangeability.. Radiology.

[326] Rajpurkar P., Chen E., Banerjee O., Topol E.J. (2022). AI in health and medicine.. Nat. Med..

[327] Lin C.C., Wang C.N., Ou Y.K., Fu J. (2014). Combined image enhancement, feature extraction, and classification protocol to improve detection and diagnosis of rotator-cuff tears on MR imaging.. Magn. Reson. Med. Sci..

