# Supplementary material for: Advances in Shoulder Pain Imaging: A Narrative Review of Current Practice and Emerging Trends
Source: Curr Med Imaging. 2025 Nov 29;21:e15734056441053. doi: 10.2174/0115734056441053251112071648 (PMC13223507; doi:10.2174/0115734056441053251112071648)
Supplement: Supplementary file 1 [file CMIM-21-E15734056441053_SD1.pdf]

1573-4056/25 *Send Orders for Reprints to reprints@benthamscience.net*

BENTHAM SCIENCE

CrossMark

Current Medical Imaging

Content list available at: https://benthamscience.com/journals/cmir

Supplementary Material

Advances in Shoulder Pain Imaging: A Narrative Review of Current Practice and Emerging Trends

António Proença Caetano[1,*], André Barros[2], Augusto Gaspar[3], Marina Ribeiro[3], Fernando Gonçalves[3], Pedro Soares Branco[4] and Vasco Vogado Mascarenhas[3]

[1]*Interventional Radiology Unit, Unidade Local de Saúde – São José, Lisboa, Portugal Radiology Department, Hospital da Luz, Lisbon, Portugal Nova Medical School, Faculdade de Ciências Médicas, Universidade Nova de Lisboa, Lisboa, Portugal*
[2]*Department of Orthopedic, Hospital da Luz, Lisbon, Portugal*
[3]*Department of Radiology, Hospital da Luz, Lisbon, Portugal*
[4]*Department of Physical Rehabilitation, Unidade Local de Saúde – São José, Lisbon, Portugal Nova Medical School, Lisbon, Portugal*

Table S1. Recent reviews and guidelines addressing shoulder pain conditions and imaging.

Author	Year	Article Type	Clinical application	Title
Cagnie *et al*. [45]	2011	Review	Functional MRI	Muscle functional MRI as an imaging tool to evaluate muscle activity
Saliken *et al*. [46]	2015	Review	Shoulder instability	Imaging methods for quantifying glenoid and Hill-Sachs bone loss in traumatic instability
McGarvey *et al*. [47]	2016	Meta-analysis	RCT and MRI / MRA	Comparison 3.0 T MRI and 3.0 T MRA for diagnosis of RCT
Garving *et al*. [4]	2017	Review	Shoulder impingement	Impingement syndrome of the shoulder
Gold *et al*. [48]	2017	Systematic review	Imaging biomarkers	Quantitative imaging biomarkers for neck and shoulder disorders
Gottsegen *et al*. [49]	2017	Review	Advanced MRI	Advanced MRI techniques for the shoulder joint
Ajuied *et al*. [50]	2018	Meta-analysis	MRI and MRA	Diagnosis of glenoid labral tears using 3.0 T MRI vs 3.0 T MRA
Pesquer *et al*. [51]	2018	Review	Shoulder impingement	Multimodality imaging of subacromial impingement
Alaia *et al*. [52]	2019	Review	Advanced MRI / MRA	Shoulder MRI and MRA techniques: new advances
Maio *et al*. [53]	2019	Systematic review	Shoulder instability	How to measure a Hill-Sachs lesion
Loriaud *et al*. [54]	2020	Review	Measurement	Critical shoulder angle – what do radiologists need to know?
Liu *et al*. [55]	2020	Meta-analysis	MRI and MRA	Comparison MRI versus MRA for the diagnosis of RCTs
Jerban *et al*. [56]	2020	Review	Advanced MRI - UTE	Qualitative imaging of bone using UTE-MRI
Vopat *et al*. [57]	2021	Systematic review	Shoulder instability	Imaging modalities for diagnosis and quantification of Hill-Sachs lesions
Daniels *et al*. [22]	2021	Review: 3D MRI	3D MRI	3D MRI of the shoulder
Familiari *et al*. [58]	2022	Review	AI and RCT	AI in management of RCT
Thacher *et al*. [59]	2023	Review	Shoulder instability	Current concepts in measurement of glenohumeral bone loss
Gupta *et al*. [61]	2023	Systematic review	AI and shoulder surgery	AI-based applications in shoulder surgery leaves much to be desired
Altmann *et al*. [62]	2023	Systematic review	MRA	ABER position in MRA – utility
Keeling *et al*. [63]	2023	Review	Shoulder instability	Bone loss in shoulder instability: putting it all together
Eckers *et al*. [64]	2023	Review	Surgery and RCT	Current understanding and new advances in the surgical management of reparable RCT: a scoping review
Rodriguez *et al*. [44]	2023	Review	AI and RCT	Artificial intelligence and Machine Learning in RCTs
Horowitz *et al*. [65]	2023	Review	Shoulder impingement	Shoulder impingement syndrome

DOI: 10.2174/0115734056441053251112071648, 2025, *21*, e15734056441053

Author	Year	Article Type	Clinical application	Title
Vosshenrich *et al*. [43]	2024	Review	Protocol optimization and MRI	Modern acceleration in musculoskeletal MRI – applications implications and challenges
Oladimeji *et al*. [60]	2024	Review	Measurement	Impact of critical shoulder angle in shoulder pathology: a current concepts review
Chang *et al*. [18]	2024	Guidelines	MRA	Guidelines for utilization and performance of direct MRA
Feuerriegel *et al*. [66]	2024	Review	Protocol optimization and MRI	Image quality: metal-related artefacts
Chen *et al*. [41]	2024	Review	AI and upper extremity	AI powered advancements in upper extremity joint MRI
Garcia *et al*. [42]	2024	Review	AI and RCT	Advancements in diagnosis and management of RCT – role of AI
Malhi *et al*. [33]	2024	Review	Advanced MRI – UTE	Tendon evaluation with UTE-MRI
Tisherman *et al*. [67]	2024	Systematic review	Measurement	There is high variability in quantitative measurement techniques in GH capsular measurements for shoulder instability
Ibounig *et al*. [68]	2024	Systematic review	GHJ and imaging	Systematic review of shoulder imaging abnormalities in asymptomatic adult shoulders (SCRUTINY): abnormalities of the GHJ
Housset *et al*. [69]	2024	Systematic review	Shoulder instability	MDI of the shoulder – a systematic review with a novel classification
Rawal *et al*. [70]	2024	Review	Shoulder instability	Current evidence regarding shoulder instability in the pediatric and adolescent population
Darbandi *et al*. [71]	2024	Systematic review	RCT and surgery	Fatty infiltration, tear size, and retraction size are significant risk factors for retear following arthroscopic RC repair

3D – 3 dimensional. ABER – abduction and external rotation. AI – artificial intelligence. CT – computed tomography. GHJ – glenohumeral joint. MDI – multidirectional instability. MRA – magnetic resonance arthrography. MRI – magnetic resonance imaging. RCT – rotator cuff tear. T – tesla. UTE – ultra-short time to echo.

Table S2. Recent studies in conventional MRI and MRA.

Author	Year	Clinical application	Patients / dataset	Findings / results
**Rotator cuff**				
Cauchon *et al*. [72]	2020	Correlation between morphological and radiological parameters with shoulder function in RCT	52 patients	A regression model was built that explains 43,3% of the variability in Constant score at diagnosis for patients with RCT
Wang *et al*. [73]	2022	Quantitative MRI indicators for partial SSC tears	576 patients (437 underwent arthroscopy, 89 patients with partial SSC tear, 50 controls)	The fissure sign on oblique sagittal MRI was the most specific sign for diagnosis of partial SSC tears; moderate sensitivity and specificity of lesser tuberosity cysts for SSC tears; thinning of SSC, fluid collection and coraco-glenoid arch and combined SSP complete tear have high specificity and moderate sensitivity
Ma *et al*. [74]	2022	Analysis of combined predictors to diagnose and predict RCTs	139 patients	Combined utilization of predictors is a better approach to diagnose and predict RCTs compared to single predictor; CSA and DRR present the strongest predictability
Minici *et al*. [75]	2023	Advantages of the use of axial traction MRI of the shoulder in patients with suspected RCT	11 patients	Significant morphological changes in the shoulder of patients with suspected RCT who underwent a GHJ axial traction MRI
Gafoor *et al*. [76]	2023	Correlation between MRI diagnosis of RC injuries and arthroscopy findings	36 patients	MRI RCT identification had a high sensitivity, specificity and positive predictive value compared to arthroscopy
Gowda *et al*. [77]	2024	RCTs: correlation between clinical examination, MRI and arthroscopy	28 patients with shoulder pain and clinically suggestive of RCT	MRI is better at predicting SSP, ISP, SSC, teres and LHB tendon appearance than clinical diagnosis in terms of sensitivity, specificity, PPV and NPV
Ben *et al*. [78]	2024	Correlation between MRI RC tendon stump classification and surgical outcomes following superior capsular reconstruction	75 patients	Patients with stump classification type 1 had better functional scores and forward flexion
**Shoulder impingement**				
Zhang *et al*. [79]	2019	CHI and CGI as predictors for different types of degenerative SSC tendon tears	235 shoulders	CHI and CGI are potential valuable predictors of the types of degenerative SSC tendon tears

Author	Year	Clinical application	Patients / dataset	Findings / results
Watson *et al.*	2019	MRI based coracoid morphology and its association with SSC tears	56 patients (33 arthroscopically proven SSC tears, 23 controls)	Acute inferior angulation of the coracoid in sagittal plane may be associated with SSC tears
Zaid *et al.* [81]	2019	Anatomic shoulder parameters and their relationship to the presence of degenerative RCTs and GHJ OA	30 articles (SR and MA)	Degenerative RCTs appear to be significantly associated with AI, CSA and LAA morphological parameters
Kim *et al.* [82]	2019	Association of RCT incidence with CSA and subacromial osteophytes	323 patients (214 with RCT, 109 controls)	RCT was affected more by osteophytes than CSA
Liu *et al.* [83]	2020	The acromion-greater tuberosity impingement index and its association with RC pathology	159 patients (83 with RC pathology and 76 with acute RCT)	ATI is a good predictor of degenerative SSP tendon tears or SAI syndrome, and can guide acromion surgery
Seo *et al.* [84]	2020	New predictors for SSC tear: coraco-lesser tuberosity angle, lesser tuberosity angle, and lesser tuberosity height	171 patients (114 with SSC tears, 57 with SIS)	There is an association between CLA and LTA and SSC tears
Vaz *et al.* [85]	2020	Effect of the acromial inferolateral tilt on shoulder impingement syndrome	346 shoulders	Inferolateral AT may have some impact on SAI; glenoacromial angle has a fair performance to assess the risk of complete SSP tendon tear
Joo *et al.* [86]	2020	Evaluation of the cross-sectional area of the acromion process for shoulder impingement syndrome	95 patients	Acromion process cross-sectional area is a newly devised parameter that is associated with higher possibility of SIS
İncesoy *et al.* [87]	2021	Association of RCT with CSA, AI, GVA and AA	870 patients (437 with RCTs and 433 with intact RC)	AI, CSA, AA and GVA may be considered risk factors for degenerative RCTs
Hufeland *et al.* [88]	2021	Acromiohumeral distance evaluation on MRI of shoulders with intact RC tendons	234 patients	AHD is significantly smaller on MRI in comparison to AP radiographs in shoulders with intact RC tendons and should not be used as a decision criterion to assess GHJ centering or subacromial space width
Ferenczi *et al.* [89]	2022	Clinical and MRI outcomes of SAI with conservative treatment	26 patients	Clinical improvement was not related to MRI changes over time
Obaid *et al.* [90]	2022	Coracoclavicular bursal changes on MRI in patients with shoulder pain and reduced coracoclavicular distance	198 shoulders	Coracoclavicular bursal changes were associated with reduced coracoclavicular distance, suggesting friction or impingement process
Miyake *et al.* [91]	2022	Alteration of CAL thickness at the acromial undersurface in patients with RCTs	213 patients	Increasing RCT size is significantly associated with CAL thickness at the acromial undersurface
El-Amin *et al.* [92]	2022	Association of coracoid impingement and morphology with fatty infiltration and RCTs	193 shoulders	Patients with anterior-superior RCT had significantly increased SSC and SSP Goutallier FI score compared to no tear and isolated SSP tears
Yu *et al.* [93]	2022	Comparison of MRI findings in shoulders with subcoracoid impingement syndrome and normal shoulders	147 shoulders (100 normal, 47 with subcoracoid impingement syndrome)	CHD and height of the lesser tuberosity were key MRI diagnostic findings for subcoracoid impingement syndrome
Xie *et al.* [94]	2022	Correlation between acromion-greater tuberosity impingement index and risk of retear after ARCR	132 patients	Acromion-greater tuberosity index is associated with risk of re-tear after ARCR
Smith *et al.* [95]	2022	Association of CSA values with full-thickness posterior-superior RCT and primary GHJ OA	10 patients	A difference was found regarding CSA between patients with symptomatic full-thickness RCTs, symptomatic GHJ OA and GHJ instability or labral pathology with no RCT or GHJ OA
Kocadal *et al.* [96]	2022	Volumetric evaluation of subacromial space in shoulder impingement syndrome	52 patients (26 controls, 26 with SIS)	There is a reduction in subacromial volume in patients with SIS; sagittal plane morphology of the acromion is more closely related to subacromial volume
Kucukciloglu *et al.* [97]	2022	Relationship between radiological measurements of subcoracoid impingement and SSC tendon lesions	298 patients	There are no relationships between radiological measurements and SSC tendon tears
Caffard *et al.* [98]	2023	Association of AS and AHD with risk of retear of the SSP tendon after ARCR	55 patients	Preoperative AHD and AS area associated with SSP tendon re-rupture after ARCR
Jäschke *et al.* [99]	2023	Association of multiple MRI parameters with shoulder function and pain in shoulder impingement syndrome	69 patients	The “halo sign” around the LHB tendon and width of the subdeltoid bursa better predict shoulder function and pain in SAI

Author	Year	Clinical application	Patients / dataset	Findings / results
Ting t al. [100]	2023	Alterations in CHL and distance in patients with symptoms of subcoracoid impingement	40 patients	CHD is related to ligament thickness in patients with SAI
Chuang *et al*. [101]	2023	Association of CAL degeneration with RCT patterns and retear rate	44 patients	CAL degeneration was more severe in anterosuperior type massive RCTs
Fallahpour *et al*. [102]	2023	Evaluation of acromion variants on MRI and their association with RC injuries in non-traumatic patients	371 patients	Tendon involvement levels increase with age and acromion type progresses to hook shape; hook-shaped acromion was related to tendinopathy level; down-sloped acromion was higher than normal variant in patients with shoulder pain
Çetinkaya *et al*. [103]	2023	Relationship between subcoracoid impingement, subacromial impingement and SSC tears	16 patients	There is no significant difference between operated and opposite-side healthy shoulders of patients undergoing SSC tear surgical repair
Çağlar *et al*. [104]	2023	Relationship of GHJ morphology and RCTs	408 patients	High AI, CSA, AA, ATI, GVA and low AHD and LAA are risk factors for RCT
Schiefer *et al*. [105]	2023	Reliability of MRI for measurement of CSA and AI	124 patients (134 shoulders)	MRI and CXR provide high intra- and interobserver agreement for measurement of AI and CSA
Lopez *et al*. [106]	2024	Anatomic factors associated with degeneration and fraying of the CAL	93 patients	Increased severity of CAL degeneration is associated with older age, larger RCT size, ISP tear, and increased preoperative CSA
Mi *et al*. [107]	2024	MRI-based coracoid process morphology and its association with isolated SSC tendon tears	60 patients	Coracoid overlap is positively associated with isolated SSC tears; coracoid bursa effusion, cysts in lesser tuberosity or a tear and malposition of LHB tendon may predict SSC tear
Sakdapanichkul *et al*. [108]	2024	Evaluation of AHD to glenoid ratio as a universal diagnostic tool for massive RCTs	145 patients	AHD-glenoid ratio is a promising diagnostic tool for massive RCTs, with improved sensitivity and specificity compared to AHD
Ozdemir *et al*. [109]	2024	Preoperative MRI evaluation of CAL degeneration	560 patients	CAL thickness decreases on MRI as arthroscopic CAL degeneration inscreases; SIS can be prediceted by looking at the CAL thickness on MRI
**Shoulder instability**				
Locher *et al*. [110]	2016	Hill-Sachs off-track lesions as risk factor for recurrence of instability after arthroscopic Bankart repair	254 patients	Off-track lesion is a significant and important risk factor for recurrence of instability and need for revision surgery after Bankart repair
Stillwater *et al*. [111]	2017	Comparison of 3D MR vs 3D CT bone models of the shoulder in patients with GHJ instability	12 shoulders	3D MRI bone models were equivalent to 3D CT bone models
Vopat *et al*. [112]	2018	Measurement of glenoid bone loss with 3D MRI matched with 3d CT	8 patients	3D MRI and 3D CT measurements of glenoid bone surface area and glenoid bone loss are nearly identical
Yang *et al*. [113]	2018	Use of hill-Sachs interval to glenoid track width ratio as a predictor of recurrent instability after arthroscopic Bankart repair	160 patients	Hill-Sachs interval to glenoid track width ratio appears to be a reliable measurement for predicting recurrent instability
Lansdown *et al*. [114]	2019	Comparison of automated 3D MRI and 3D CT to evaluate glenoid bone loss	16 patients	3D MRI reconstructions of the glenoid can evaluate glenoid bone loss with similar performance compared to 3D CT reconstructions
Dyrna *et al*. [115]	2021	Off-track HSL measurement and recurrence after nonoperative management of first-time anterior shoulder dislocations	54 patients	Off-track Hill-Sachs lesion leads to significantly higher recurrence rates compared to on-track or no Hill-Sachs lesion in patients with nonoperative management
Karahan *et al*. [116]	2021	Evaluation of CSA and AI in patients with anterior shoulder instability and RCT	100 patients (50 with RCT and 50 with anterior instability)	Lower AI, GI and antevert glenoid version may be associated with anterior instability
Sgroi *et al*. [117]	2022	Accuracy of glenoid bone loss assessment with MRI	80 patients	CT and MRI can accurately detect glenoid bone loss
Cohn *et al*. [118]	2022	MRI analysis of native glenoid anatomy and predisposition to shoulder instability	214 patients (107 instability patients, 107 controls)	Males with instability had smaller glenoid width and surface are, and glenoid width was proportionally smaller relative to humeral width; bony GH morphology did not appear to be a risk among females

Author	Year	Clinical application	Patients / dataset	Findings / results
Ishikawa *et al.* [119]	2023	Association of RC muscle imbalance and shoulder instability direction	189 patients (63 per group; anterior, posterior and multidirectional instability)	Patients with anterior instability have smaller muscle area of the posterior RC compared to anterior RC; the contrary was found for posterior instability
Kim *et al.* [120]	2024	RC muscle imbalance evaluation in patients with chronic anterior shoulder instability	56 patients	Larger areas of SSP muscle compared with acute instability were observed in patients with chronic anterior shoulder instability
Bhatia *et al.* [121]	2024	Comparison of dynamic arthroscopic tracking and radiologic glenoid track to identify on and off-track lesions in anterior shoulder instability	114 patients	Incorporating a standardized arthroscopic tracking method into current algorithms may help reduce variability in surgical decision making
**Magnetic resonance arthrography**				
Park *et al.* [122]	2019	Evaluation of inferior capsular laxity in patients with atraumatic MDI	130 patients (65 with MDI, 65 controls)	CSA and GCR were significantly higher in patients than controls; GCR was more sensitive and specific for detection of MDI than CSA
Wengert *et al.* [123]	2019	Diagnostic reliability of US and MRA for shoulder injuries in sports patients	67 patients with clinically suspected labra lesions, RCT or LHB tendon injury	Moderate to substantial correlation and diagnostic accuracy for assessment of shoulder joint disorders carried out with US and MRA; good agreement was achieved in RC lesions
Celentano *et al.* [124]	2022	Evaluation of inferior capsular width with MRA in patients with MDI	176 patients	Inferior capsular redundancy may be an important predisposing factor in MDI, while glenoid circumference is not related to MDI
Dwivedi *et al.* [273]	2024	Evaluation of shoulder injuries: MRI and MRA comparative study	100 patients	MRA is a non-surgical effective method in evaluating and diagnosing RC injuries in comparison to MRI
Yamamoto *et al.* [126]	2024	Glenoid track and subcritical HSL	1332 patients	3.0 T MRI appears equivalent to 3.0 T MRA in diagnosis of full and partial-thickness RCTs, with a trend for greater accuracy in diagnosis of SSC tears with MRA
**Other**				
Ozel *et al.* [127]	2020	Implications of the glenoid angles and RC status in patients with OA undergoing arthroplasty	202 patients (231 shoulders)	3D evaluation of glenoid inclination is mandatory for preoperative planning of shoulder replacement, and reverse arthroplasty may be considered more frequently than previously expected, even when RC is intact
Kapoor *et al.* [128]	2024	Posterior capsule edema in adhesive capsulitis – comparison with established non-contrast MRI findings and multivariable analysis	114 patients (57 with AC, 57 controls)	Posterior joint capsule edema may be helpful to confirm AC; posterior capsule edema, CHL edema, and axillary pouch thickness produce a strong model for distinguishing AC from controls

3D – 3 dimensional. AA – acromial angulation. AC – adhesive capsulitis. AI – artificial intelligence. AP – antero-posterior. ARCR – arthroscopic rotator cuff repair. AS – acromial slope. AT – acromial tilt. ATI – acromion-greater tuberosity index. CAL – coracoacromial ligament. CHD – coracohumeral distance. CHL – coracohumeral ligament. CGI – coracoglenoid inclination. CHI – coracohumeral interval. CLA – coracoid-lesser tuberosity angle. CSA – critical shoulder angle. CT – computed tomography. CXR – conventional radiography. DRR – double circle radius. FI – fatty infiltration. GCR – glenocapsular ratio. GHJ – glenohumeral joint. GVA – glenoid version angle. HSL – hill-sachs lesion. ISP – infraspinatus. LAA – lateral angle of the acromion. LHB – long head of the biceps. LTA – lesser tuberosity angle. MA – meta-analysis. MDI – multidirectional instability. MRA – magnetic resonance arthrography. MRI – magnetic resonance imaging. NPV – negative predictive value. OA – osteoarthritis. PPV – positive predictive value. RC – rotator cuff. RCT – rotator cuff tear. SAI – subacromial impingement. SIS – subacromial impingement syndrome. SR – systematic review. SSC – subscapularis. SSP – supraspinatus. US – ultrasound. T – tesla.

Table S3. Recent studies in advanced MRI techniques.

Author	Year	Clinical application	Patients / dataset	Findings / application
**Contrast**				
Sasanuma *et al.* [129]	2017	Dynamic-MRI characteristics of idiopathic severe frozen shoulder	21 patients (5 healthy volunteers and 16 patients with idiopathic severe FSh)	Dynamic-MRI demonstrated the pathologic significance of the burning sign associated with severe FSh, and may be associated with pain and inflammation in idiopathic FSh
Sasanuma *et al.* [130]	2018	Blood flow evaluation of symptomatic RCT and frozen shoulders	61 patients (31 symptomatic RCT and 30 with FSh)	The burning sign occurred in the rotator interval and axillary pouch in all cases of FSh and approximately 50% of cases with RCT. Burning sign in symptomatic RCT occurred in the rotator interval and was associated with pain score, tear size and ROM

Author	Year	Clinical application	Patients / dataset	Findings / application
Lee *et al*. [131]	2020	Voxel-based 3D segmentation of capsulo-synovium from contrast-enhanced MRI in adhesive capsulitis	169 patients	Voxel-based 3D segmentation of entire capsulo-synovial enhanced portion from CE-MRI can represent the severity of clinical impairments in AC
Saito *et al*. [132]	2020	Dynamic-MRI characteristics of symptomatic chronic calcifying tendinitis	6 patients	Dynamic-MRI identified 2 types of findings in patients with symptomatic chronic calcifying tendinitis - burning sign at rotator interval and axillary pouch or around calcium deposit
Saito *et al*. [133]	2021	Dynamic-MRI characteristics of post-traumatic shoulder stiffness	5 patients	All patients had abnormal enhancement at the rotator interval and axillary pouch
Iijima *et al*. [134]	2022	Changes in abnormal blood flow in FSh after shoulder manipulation under US-guided cervical nerve root block	19 patients with FS	Dynamic-MRI semiquantitative evaluation demonstrated a reduction in abnormal blood flow and improvement in clinical results after block in patients with FSh
Erber *et al*. [19]	2024	Diagnostic performance and interreader agreement of individual and combined non-enhanced and contrast-enhanced MRI parameters in adhesive capsulitis	180 patients (60 with and 120 without AC)	Contrast-enhanced signs and findings have higher agreement among readers and diagnostic performance compared to non-enhanced imaging signs
**Radial**				
Furukawa *et al*. [135]	2014	Detection of SSC tendon tear	54 patients (55 shoulders)	Radial MRI has high sensitivity for SSC tears, and higher than conventional orientations for the superior part
Honda *et al*. [136]	2015	Detection of RCTs	48 patients (18 patients with normal cuff and 30 with RCT)	Adequate 0º to 120º could be visualized in radial sequences, compared to the posterior portions of the RC insertions at 45º that could not be adequately visualized in some coronal oblique and axial sequences
Ogura *et al*. [137]	2018	Validity of radial MRI to determine extent of bankart lesions	75 patients (45 patients without labrum injury and 30 patients with bankart lesions)	Radial images enabled precise assessment of injured glenoid labra, except at the anterosuperior labrum, and permitted wider range of anteroinferior labrum morphology and injury assessment
Sasaki *et al*. [138]	2018	Evaluation of RCT morphology	52 patients (52 shoulders)	Tear morphology was visualized in 100% of radial sequences and only 46% in axial and coronal oblique sequences, and radial MRI was better at characterizing tear morphology with length ≥ 3 cm
Shibayama *et al*. [139]	2022	Relationship between preoperative RCT size using radial imaging and postoperative RC integrity	102 patients	Pre-operative tear area was independently correlated with rate of retear after shoulder ARCR, evaluated by radial MRI
Shibayama *et al*. [21]	2022	Diagnostic accuracy of radial imaging for partial tears of the LHB tendon in patients with RCT	118 patients	Radial MRI has higher sensitivity compared to axial, sagittal and coronal oblique sequences for diagnosing LHB tendon partial tears
Takeshima *et al*. [20]	2023	Efficacy of biceps-radial slice MRI for the diagnosis of biceps and pulley lesions	84 patients	Biceps-radial slices allow tracking of the LHB tendon and pulley from the supraglenoid tuberosity to the bicipital groove in the GHJ and accurate evaluation of lesions
**3D applications**				
Gyftopoulos *et al*. [140]	2016	RCT shape characterization: comparison of 2D sequences and 3D MR reconstructions	34 patients	3D MR reconstructions of the RC improve characterization of RCT shapes compared to conventional 2D MRI
Schleich *et al*. [141]	2017	Assessment of GHJ cartilage thickness distribution	38 patients	There is radial mismatch between the joint surface of the humeral head and glenoid cavity at the cartilaginous level; cartilage distribution is homogeneous except at posterior aspect of humeral head
Lansdown *et al*. [114]	2019	Automated 3D MRI evaluation of glenoid bone loss	16 patients	3D MRI reconstruction can evaluate glenoid bone loss similarly to the performance of 3D CT reconstruction
Khanna *et al*. [142]	2019	Development of a 3D method to assess intramuscular spatial distribution of fatty infiltration in RCT	13 patients	Current study method to quantify 3D spatial distribution of muscle pathophysiology is feasible and useful to reliably quantify RC muscle degeneration
Wuennemann *et al*. [143]	2019	Diagnostic performance of 3D-MEDIC for SLAP lesions	17 patients	Sensitivity and NPV was higher for 3D-MEDIC compared to the respective diagnostic values of the 2D FS PD images.

Author	Year	Clinical application	Patients / dataset	Findings / application
Henninger *et al*. [144]	2020	Prediction of muscle volume through muscle cross-sectional area evaluation on MRI	10 fresh-frozen human cadaver specimens from 10 patients	Simple linear equations were derived that allow cross-sectional areas of the deltoid and RC muscles to predict 3D muscle volumes with low error
Wallenberg *et al*. [145]	2022	Assessment of fatty infiltration distribution and muscle atrophy in RCTs	109 patients	Distribution of fat content in SSP muscle appears to change once the global fat content of the muscle increases >10% (more uniformly) compared to <10% (more lateral/myotendinous junction)
Feuerriegel *et al*. [146]	2022	Comparison of different measures on simulated CT-like images, simulated radiographs and CXR in shoulder pain	25 patients with suspected shoulder pathology	Measurements of simulated radiographs and CXR showed substantial to almost perfect inter- and intra-rater agreement
Rosenthal *et al*. [147]	2022	Comparison of 3D MRI and 3D CT in evaluation of glenoid deformity in GHJ OA using Dixon 3D FLASH sequence	29 patients	3D MRI is comparable to 3D CT with respect to axial glenoid bone loss, as measured by glenoid version; 3D CT remains gold standard for coronal bone loss estimation
Lander *et al*. [148]	2022	Comparison of CT and 3D MRI evaluation of GHJ instability bone loss	18 patients	3D MRI measurements for bone loss in GHJ instability through use of VIBE sequences were equivalent to 3D CT; 3D MRI was 1.67 times cheaper than MRI and CT with 3D reconstruction
Riem *et al*. [25]	2023	Objective analysis of RC muscle volume and fatty infiltration	170 healthy shoulders	Lateral characteristics of RC musculature demonstrated diminished muscle size with age and increased fatty infiltration in females; strong significant relationship of the expected lateral 30-40% RC muscle in cases with complete coverage
Kim *et al*. [149]	2023	Adequacy of 3D MRI evaluation for preoperative planning in distal clavicle autograft versus traditional and congruent arc latarjet procedures	24 patients	3D MRI is a viable method for preoperative planning and graft selection in glenoid bone loss
Nizinski *et al*. [26]	2024	Reliability of glenoid measurements using MRI multiplanar reconstructions	80 patients with anterior shoulder instability	High-resolution MPR MRI measurement of the anterior glenoid lesion is a very good tool for linear glenoid parameters, resistant to the human factor and low experience; Pico index measurement is unreliable; complex measurements are not resistant to low experience
Nagawa *et al*. [150]	2024	3D sectional measurement analysis for serial volume changes in shoulder muscles after ARCR	31 patients (33 shoulders)	Perioperative muscle volume changes in SSP and ISP were different from those in teres minor and SSC; postoperative differences might correlate with tear size and symptom duration in the SSP, history of steroid injections and work and sports activity levels for ISP and teres minor
Paul *et al*. [151]	2024	Comparison of shoulder instability preoperative planning with 3D CT or 3D MRI	11 surgeons surveyed	There is significant variability in surgical procedure selection driven by time alone in shoulder instability. Surgical decision making with 3D MRI was similar to 3D CT
Khandare *et al*. [152]	2024	Utility of a novel 3D MRI-based approach to assess SSP muscle length	6 patients	Novel and reliable approach to quantifying SSP muscle length and preliminary demonstration of its utility by assessing muscle length changes associated with RC pathology and surgical repair
Haider *et al*. [153]	2024	Correlation of single-Plane 3D isotropic spin-echo MRI of the shoulder with surgical findings compared to 2D Dixon multiplanar MRI	72 patients	3D MRI of the shoulder has shorter acquisition time and exhibits comparable results with superior correlation to surgical findings than 2D MRI for detection of labral tears and some RCTs
Xu *et al*. [24]	2024	Longitudinal changes in overall 3D SSP muscle volume and intramuscular fatty infiltration after ARCR	47 patients	3D SSP volume and 3D FI showed no longitudinal change between the preoperative and 12-month follow-up after ARCR; 3D volume decreased initially but recovered at 12 months; 3D FI was stable
Cui *et al*. [23]	2024	Comparison of 3D MRI fast field echo resembling a CT using restricted echo-spacing sequence with 3D CT for quantification of bone loss and shoulder morphology in patients with shoulder dislocation	56 patients	3D MRI (FRACTURE) is equivalent to 3D CT for bone loss quantification and shoulder morphologic measurements in patients with shoulder dislocation
**Fat quantification**				
Nozaki *et al*. [281]	2015	Quantification of SSP muscle fatty degeneration using a 2-point Dixon method on 3.0 T MRI	359 patients	Increase in SSP fatty degeneration was statistically significantly correlated with severity of SSP tears and moderately correlated with muscular atrophy

Author	Year	Clinical application	Patients / dataset	Findings / application
Nozaki *et al.* [154]	2016	Retear prediction after repair of full-thickness RCT using 2-point Dixon MRI quantification of fatty muscle degeneration	50 patients	MRI quantification of preoperative fat fractions by using 2-point Dixon method may be a viable method for predicting postoperative retear of the SSP tendon
Gilbert *et al.* [155]	2016	Comparison of MRI-based Goutallier classification with experimental quantification MR spectroscopic fat measurement of the SSP muscle	42 patients	Correlation between semi-quantitative Goutallier system and MR spectroscopic fat measurement is weak.
Karampinos *et al.* [156]	2017	Association of PD fat-fraction of RC muscles with isometric strength 10 years after ARCR	13 patients	RC muscle PD fat fraction is associated with isometric strength independently of muscle atrophy and tendon rupture in shoulders with early and advanced degenerative changes
Kalin *et al.* [157]	2019	Multiecho Dixon-based fat quantification of intact RC muscles	40 patients	Quantitative MRI with estimation of fat fraction and DTI parameters shows significant age-associated changes among visually intact RC muscles
Wieser *et al.* [158]	2019	Changes of SSP muscle volume and fat fraction after successful or failed ARCR	40 patients (19 failed and 21 healed arthroscopic SSP repairs)	After ARCR, RCT-induced FI can be almost stopped and muscle atrophy can be slightly reversed
Lansdown *et al.* [159]	2019	Association of preoperative IDEAL MRI RC muscle fat fractions with RC repair outcomes	80 patients	Intramuscular fat is an important factor in postoperative outcomes even in patients with lower levels of preoperative fatty infiltration
Monroe *et al.* [29]	2020	Dependance of arthroscopic SSC tendon repair outcomes on RC fatty infiltration	140 shoulders	ARCR of SSC tendon with poor ISP muscle quality have worse patient-reported outcomes
Davis *et al.* [28]	2021	Correlation of quantitative versus semi-quantitative measures of SSP intramuscular fatty infiltration with shoulder ROM and strength: a pilot study	37 patients	SSP Dixon fat-fraction has superior correlation with shoulder ROM and strength relative to Goutallier grade on MRI
Liu *et al.* [160]	2022	Advantages of 3D measurements for SSP intramuscular fat evaluation in patients with medium to massive RCT - comparison with single sagittal slice	-	3D fat-fraction shows inhomogeneous distribution of intramuscular fat; 2D fat-fraction was higher than 3D fat-fraction in large and massive RCTs; 3D fat-fraction was more reliable for estimating FI in the SSP
Addona *et al.* [161]	2023	3D SSP intramuscular fatty infiltration assessment in older adults	10 patients	SSP 3D Dixon fat-fraction has strong correlation with 2D Dixon fat-fraction and 2D Goutallier grading
Hochreiter *et al.* [27]	2024	Natural history of fatty infiltration and 3D muscle volume after nonoperative treatment of symptomatic RCTs	79 shoulders	Risk factors for relevant FI progression were identified
**T2 mapping**				
Matsuki *et al.* [162]	2014	Quantitative evaluation of fatty degeneration of the SSP and ISP muscles using T2 mapping	180 patients, 184 shoulders (83 without tears, 101 with tears)	T2 values of SSP and ISP significantly increase with the extent of RCT
Iijima *et al.* [163]	2017	Differences in fatty degeneration of RC muscles at different sites	675 patients, 702 shoulders (357 with tears and 345 without tears)	Degree of fatty degeneration at the Y-view and 14 mm medially was similar, although significant differences between sites were detected in larger rotator cuff tears; diagnostic value of T2 measurement at the medial site is comparable to that at Y-view
Ganal *et al.* [164]	2016	Comparison of tendon tears, tendinosis and asymptomatic SSP tendons with T2 mapping	15 patients	T2 mapping accurately quantifies early RC pathology, differentiating partial and full-thickness tears from tendinosis and normal SSP tendons
Kang *et al.* [165]	2016	T2 mapping of articular cartilage of the GHJ at 3.0 T MRI in healthy volunteers	15 patients	Mean T2 values of the humeral head and glenoid cartilage were similar to quantitative T2 values reported in femoral and talar cartilage
Karampinos *et al.* [156]	2017	Evaluation of PD fat-fraction of RC muscles after surgical repair	13 patients	Correlation of more severe fatty infiltration of RC muscles with clinical shoulder abduction strength measurements in patients with early or advanced OA Higher PD fat-fraction of SSP remained significantly associated with reduced physical shoulder function; PD fat-fraction of SSP correlated with cartilage scores at the humerus
Krepkin *et al.* [166]	2017	Quantitative assessment of SSP tendon T2* values and shear-wave US elastography	8 patients	There is a potential relationship between T2* values and shear wave velocity values in the SSP tendon
Iijima *et al.* [167]	2019	Relationship between postoperative retear and preoperative fatty degeneration in massive RC tears	107 patients (83 with large and 34 with massive RCT)	Preoperative T2 values of retear group were significantly higher than the intact group in SSP and ISP

Author	Year	Clinical application	Patients / dataset	Findings / application
Lee *et al*. [32]	2019	Quantitative T2 mapping of articular cartilage of the GHJ at 3.0 T in patients with RC disease	62 patients	T2 mapping of the GHJ does not show statistically significant differences in quantitative analysis of the degenerative changes of articular cartilage based on degree of RCT
Lockard *et al*. [31]	2021	T2 mapping of GHJ cartilage in asymptomatic patients and patients with RC pathology	59 patients (15 asymptomatic, 10 tendinosis, 13 partial tear, 8 full-thickness tear, 13 massive tear)	Massive tear group T2 values were significantly higher than asymptomatic group values for the humeral head cartilage; mean T2 was higher for massive compared to full-thickness tear, partial tear, and tendinosis
Matsuki *et al*. [30]	2024	Fatty degeneration of RC muscles after ARCR	103 patients (103 shoulders, 13 partial, 18 small, 34 medium, 33 large, 4 massive tears)	Shoulders with successful repair had significantly smaller post-operative T2 values, and improved ER strength; fatty degeneration can be partially reversed
**UTE / ZTE**				
Breighner *et al*. [168]	2018	Enhancement of osseous detail	34 patients	ZTE MRI provides CT-like contrast for bone
Zhu *et al*. [169]	2018	RC tendon assessment (ex vivo)	20 cadaveric rotator cuff tendons	MMF obtained with 3D UTE technique is resistant to the magic angle effect and is more sensitive to RC tendon degeneration
Ma *et al*. [170]	2018	Quantification of glenoid bone loss	11 cadaveric shoulder specimens (11 patients), 3 patients	3D IR-UTE-Cones selectively visualizes lamellar bone and produces similar contrast to 2D CT in glenoid bone; evaluation of glenoid width is similar to measurement in 3D and 2D CT, compared to 2D T1WI
Guo *et al*. [171]	2019	RC tendon degeneration evaluation with biochemical and histological correlation (in vitro)	36 pieces of SSP tendons from 6 cadaveric donors	MMF, adiabatic T1rho and T2* measured and US BSC can detect alterations in collagen; MMF showed highest correlation with collagen loss
Ashir *et al*. [172]	2020	Comparison of RC tendon assessment in healthy versus symptomatic patients	24 patients, 48 shoulders (14 with unilateral tendinopathy, 10 controls)	Symptomatic RC tendinopathy shoulders had lower MMF values and higher T2 values compared with controls; the contralateral shoulder of patients with unilateral tendinopathy can demonstrate asymptomatic tendinopathy quantified by MMF or T2
Mello *et al*. [173]	2020	Comparison of 3D ZTE MRI sequence and 3D CT for glenoid bone assessment	6 shoulder specimens (cadaveric), 10 GHJ instability patients	3D MRI and 3D CT techniques offer similar results; ZTE MRI technique is very promising with high quality 2D images; there is no apparent difference between MRI obtained at 0,6-0,7 mm, 0,9 mm and 1 mm isotropic resolutions
Xie *et al*. [174]	2020	Evaluation of tendon healing after ARCR	40 patients (25 patients with ARCR, 15 healthy controls)	UTE-T2* can detect changes in the process of tendon healing of the repaired RC postoperatively
Yildiz *et al*. [175]	2022	Optimization of Grashey view radiograph for CSA measurement by comparison with ZTE sequences	65 patients	CSA measurement on ZTE MRI scans with anatomic point cross-referencing was significantly different from that on Grashey view radiographs, even with optimal positioning
Xie *et al*. [35]	2023	Comparison of RC tendon signal values between suture-bridge and single-row repair techniques	31 patients (17 suture-bridge and 14 single-row repair)	In early postoperative period, higher T2* values in the proximal region of repaired tendon were seen in suture-bridge technique compared to single-row, but was resolved over time
Feuerriegel *et al*. [34]	2023	Comparison of UTE sequences and CT for evaluation glenoid bone loss and other osseous pathologies	46 patients	Evaluation of bony Bankart lesions and other osseous pathologies of shoulder is feasible and accurate using CT-like images based on a T1 GRE, UTE and FRACTURE sequence compared to conventional CT
**Cine-MRI**				
Tempelaere *et al*. [39]	2016	Dynamic 3D shoulder MRI during active motion for investigation of RC diseases	20 shoulders (14 shoulders with pathology and 6 normal shoulders)	Dynamic MRI technique enabled a novel measure, “looseness”, i.e., translation of the humeral head on the glenoid during an abduction cycle, and was able to distinguish different forms of RC disease
Matsui *et al*. [38]	2018	Translational movement within GHJ at different rotation velocities	10 healthy patients, bilateral shoulder MRI	Translation undergoes no significant change throughout the ranges of internal and external rotation or among different rotational velocities

Author	Year	Clinical application	Patients / dataset	Findings / application
Kenmoku *et al.* [37]	2019	Comparison of GHJ rotation between asymptomatic and SAI syndrome patients	183 shoulders (73 shoulders in 42 asymptomatic patients and 110 SAI syndrome shoulders in 103 patients)	SAI syndrome shoulders have significantly restricted GHJ rotation compared to asymptomatic shoulders, and may be associated with RC dysfunction
Ishii *et al.* [36]	2021	Limitation of the external GHJ rotation is associated with SAI syndrome	95 patients (100 shoulders)	ER of shoulders with SAI syndrome was significantly positively correlated with the Constant-Murley UCLA scale and night pain
Kenmoku *et al.* [176]	2023	Kinematic analysis of damaged capsulolabral structure in patients with anterior shoulder instability	28 patients (29 shoulders)	Immobilization of the arm in ER can enhance compression of the damaged labrum on the glenoid rim better than IR; some patients may benefit from IR immobilization
**Functional**				
Tawara *et al.* [40]	2021	Feasibility of muscle functional MRI for exercise-induced RC muscles	8 patients	High detectability of muscle activity in the shoulder using T2 signal calculated from MSSE-EPI images
**Protocol optimization**				
Sartoretti *et al.* [177]	2019	Reduction of procedure time in routine clinical practice with C-SENSE technique	2519 exams (1109 before C-SENSE and 1410 after C-SENSE implementation)	C- SENSE allows significant acceleration of MR examinations and a considerable increase in the total number of MR examinations is possible
Gao *et al.* [178]	2021	Comparison of high-resolution SMS accelerated TSE with routine TSE imaging	71 patients	SMS-TSE sequence can significantly reduce scanning time with similar image quality compared to TSE sequences
Niitsu *et al.* [179]	2022	Performance of motion-robust MRI of the shoulder using C-SENSE MultiVane	31 patients	Newly developed C-SENSE MV technique reduces motion artifacts and increases the detectability of anatomical structures compared with standard sequences
lazikHou *et al.* [180]	2022	Comparison of CAIPIRINHA-accelerated 3D FS-SPACE MRI with 2D MRI sequences for shoulder pathology	49 patients	Performance of 6-minute 3D FS-CAIPIRINA SPACE is similar to that of 10-minute 2D TSE MRI
Kohli *et al.* [181]	2022	Comparison of BLADE and rectilinear techniques at 3.0 T scanning to improve image quality and motion degradation reduction in shoulder MRI	57 patients	BLADE reduces motion degradation and improves image quality
Liu *et al.* [182]	2023	Performance of 3D SPACE MRI with CAIPIRINHA fourfold acceleration for assessing LHB tendon	42 patients	Fourfold acceleration CAIPIRINHA reduces acquisition time of SPACE MR in the shoulder, and is highly accurate in detecting LHB tendon conditions
**Other**				
Lazik-Palm *et al.* [183]	2020	7.0 T evaluation of suspected RC lesions	8 patients	7.0 T MRI of the shoulder with diagnostic image quality is feasible; diagnostic performance did not reach the current results of 3.0 T MRI

2D – 2 dimensional. 3D – 3 dimensional. 3D-MEDIC – 3D multiple echo data image combination. AC – adhesive capsulitis. ARCR – arthroscopic rotator cuff repair. BLADE – balanced steady state free precession line acquisition with undersampling. CAIPIRINHA – controlled aliasing in parallel imaging results in higher acceleration. CE – contrast-enhanced. C-SENSE – compressed sensing. CT – computed tomography. CXR – conventional x-ray. DTI – diffusion tensor imaging. EPI – echo-planar imaging. ER – external rotation. FI – fatty infiltration. FLASH – Fast low angle shot. FRACTURE – fast-field-echo resembling a CT using restricted echo-spacing. GRE – gradient recalled echo. IDEAL – iterative decomposition of water and fat with echo asymmetry and least-squared estimation. LHB – long head of the biceps. FS – fat-saturated. FSh – frozen shoulder. GHJ – glenohumeral joint. IR – internal rotation. ISP – infraspinatus. MMF – macromolecular fraction. MPR – multiplanar reconstruction. MRI – magnetic resonance imaging. MSSE – Multisection excitation by simultaneous spin-echo interleaving. MV – multivane. NPV – negative predictive value. OA – osteoarthritis. PD – proton density. RC – rotator cuff. RCT – rotator cuff tear. ROM – range of motion. SAI – subacromial impingement. SIS – subacromial impingement syndrome. SMS – simultaneous multislice imaging. SPACE – sampling perfection with application optimized contrast using different flip angle evolution. SSP – supraspinatus. T – tesla. US – ultrasound. UTE – ultra-short time to echo. VIBE – volumetric interpolated breath-hold examination. ZTE – zero-echo time.

Table S4. AI and shoulder MRI-related articles.

Author	Year	Technique	Clinical application	Patients / dataset	Findings / application
**Segmentation**					
Rodrigues *et al.* [184]	2020	CNN	Accuracy of 3D bone models for GHJ	100 patients	Feasibility of a time-efficient, fully automated MRI segmentation method for the GHJ based on CNNs that creates accurate 3D MRI models and reliable quantifications of glenoid bone loss

Author	Year	Technique	Clinical application	Patients / dataset	Findings / application
Conze *et al*. [186]	2020	Convolutional encoder-decoder	Healthy versus pathological learning transferability in shoulder muscle MRI segmentation	12 patients (24 shoulders)	Features extracted from unimpaired limbs are suited for pathological anatomies while acting as efficient data augmentation strategy; deeper networks are able to efficiently discriminate individual muscles from surrounding anatomical structures
Medina *et al*. [185]	2021	CNN, U-net-based CNN	Segmentation of rotator cuff muscles with MRI	258 cases (model A), 1048 cases (model B)	A CNN classification method was able to select an appropriate shoulder Y-view and a U-net-based CNN accurately segmented multiple RC muscles at that level
Wang *et al*. [187]	2021	PCNN, FCN, AlexNet segmentation model, U-Net bone segmentation model	Automatic segmentation of GHJ bone with MRI	100 images	Models accurately locate the shoulder humerus head and articular bone from four segmentation models
Mu *et al*. [188]	2021	U-Net, Alex Net	Automatic segmentation of GHJ bone with MRI	100 images	Models accurately located the shoulder and humeral head and articular bones, and the spatial coherence of the image sequence was used to predict the required area
Kim *et al*. [189]	2022	3D U-Net CNN	Automatic segmentation of RC tendon in 3D MRI	56 patients with RCT	Acceptable accuracy and reproducibility in automated segmentation of the RC tendon that could help clinicians reliably determine the cuff tendon tear pattern
Lee *et al*. [190]	2023	3D U-net CNN	Segmentation of posterosuperior RCT with 3D MRI	303 patients with RCT	Satisfactory outcomes for proposed DL model for RCT segmentation; software successfully visualized RCT lesions through 3D reconstructed image and provided information about tear shape and size
Hess *et al*. [191]	2023	nn-U-net	DL-based segmentation with inference accuracy prediction	171 MRAs (101 trained, 70 tested)	High accuracy on anisotropic diagnostic MRI from different centers with high variety of slice thicknesses and in-plane resolutions, comparable to other reports
Wong *et al*. [192]	2023	2D and 3D CNN	Comparison of MRI and CT DL bone shape models	55 patients	Augmentation of imaging data significantly improved 3D but not 2D model performance
Riem *et al*. [25]	2023	3D U-Net	Automatic 3D segmentation of RC muscle and fat from clinical MRI scans	232 patients (63 normal RC, 169 RCT)	Good performance in 3D segmentation and quantification of RC muscles and their respective FI from clinical MRI scans. SSP 2D FI and Goutallier grade showed significant yet weak correlation
Sezer *et al*. [193]	2023	Mask R-CNN	Mask region-based segmentation of humerus and scapula from PDWI axial shoulder MRI	665 patients (665 axial PDWI sequences)	Mask R-CNN-based segmentation of the humeral head and scapula from PDWI shoulder MRI had high success
Alipour *et al*. [194]	2024	CNN U-nets	Automatic MRI-based RC muscle segmentation	157 patients (79 healthy, 78 partial RCT)	Segmentation of SSP, SSC and ISP was possible with a level of precision akin to that of experienced radiologists
**Diagnosis / clinical**					
Sezer *et al*. [195]	2019	CapsNet	Capsule network-based classification of RC pathologies from MRI	1006 shoulders	Proposed CapsNet for the classification of RC injuries from coronal PD WI was found to be successful and the results were similar to those of an expert with a sensitivity of 98.66%
Kim *et al*. [196]	2019	CNN (FCN)	Automatic muscle atrophy measuring algorithm to calculate ratio of SSP / supraspinous fossa	240 patients	Fossa region and muscle region of the SSP Y-view of the RC muscle were detected by DL, as well as occupational ratio, providing a quantitative index to help clinicians
Kim *et al*. [222]	2020	VGG-16	MRI-based diagnosis of RCTs using DL and weighted linear combinations	2447 patients (1628 normal and 819 with RC tear)	For computer-aided diagnosis of RCTs in 3D MRI scans, use of a CNN with a weighted linear combination layer is able to produce higher diagnostic accuracy than human annotators, make a distinction between grade of RCT, and localize the pathological lesions
Shim *et al*. [197]	2020	VRN-based 3D CNN	Automated 3D RCTs classification with MRI	2124 patients	VRN-based 3D CNN outperformed orthopedists specialized in shoulder and general orthopedists in binary accuracy, top-1 accuracy, sensitivity, specificity precision, and diagnostic time

Author	Year	Technique	Clinical application	Patients / dataset	Findings / application
Ro *et al*. [198]	2021	CNN based on VGG109 network	DL framework and CA fatty infiltration analysis for supraspinatus muscle	240 patients	The proposed CNN exhibited fast and accurate segmentation of the SSP muscle and fossa, allowing automatic calculation of the occupation ratio
Key *et al*. [199]	2022	ViVGG19 feature extraction	Novel exemplar deep feature extraction-based shoulder RCT and LHB tendinosis detection using MRI	112 patients	The authors developed an effective model for RCT and LHB tendon disorders on shoulder MRI.
Yao *et al*. [200]	2022	DL reconstruction	Evaluation of a DL method for the automated detection of SSP tears on MRI	200 shoulders	DL is a feasible approach for automated detection of SSP tears on MRI
Saavedra *et al*. [201]	2023	CNN	Accuracy of CNN models to detect SSP fatty infiltration in shoulder MRI	900 MRI studies	CNN models demonstrated high accuracy in SSP muscle FI diagnosis
Lee *et al*. [203]	2023	YOLOv8 model	Automated RCT screening in 3 planes of shoulder MRI	794 patients (694 with RCT, 100 without RCT)	High sensitivity and accuracy in RCT detection model with all-plane training
Guo *et al*. [204]	2023	2D and 3D model based on Xception	Automatic diagnosis of SSP tears	770 shoulders (701 non-surgical, 69 post-surgical)	Adequate and efficient automatic diagnoses of SSP tears with comparable performance of junior MSK radiologists and orthopedic surgeons
Esfandiari *et al*. [205]	2023	CNN	MRI detection of RCTs using CNN	150 shoulders	Proposed CNN could diagnose shoulder injury from MRI images with acceptable accuracy, and was higher than the two light-weight pre-trained CNNs (SqueezeNet and MobileNetv2) used for comparison
Lin *et al*. [206]	2023	DL reconstruction	DL diagnosis and classification of RCT on shoulder MRI	11925 scans (11405 for development, 520 for final testing)	DL diagnosis of RCTs is feasible with excellent diagnostic performance, with model accuracy similar to subspecialty-trained musculoskeletal radiologists
Ni *et al*. [202]	2024	SLAP-Net	SLAP lesion detection in shoulder MRA using DL	514 patients	SLAP-Net has good diagnostic performance in identifying SLAP lesions, and the performance of this model is comparable to senior radiologists
Oeding *et al*. [207]	2024	ML model	Prediction of SSC tears based on preoperative imaging parameters (MRI)	202 shoulders	ML model can predict SSC tears in 85% of patients indicated for ARCR, and most important factors for prediction were direct changes in tendon morphology and signal, LHB tendon changes and MRI field strength
Fei *et al*. [208]	2024	Radiomics model	Novel MRI methods to diagnose RCT and predict postoperative re-tear	101 patients	Four-feature-based radiomics signature was observed to be effective for diagnosis of RCT, and classifies patients into RCT and non-RCT with AUC of 0,979, comparable to experienced radiologists
Zhang *et al*. [209]	2024	Edge-Variational GCN	DL prediction of re-tear after ARCR	1631 patients	The GCN algorithm can accurately predict RCT recurrence after ARCR before surgery, with 96,93% accuracy
**Protocol optimization**					
Koch *et al*. [210]	2021	DL network	Analysis and evaluation of a DL reconstruction approach with denoising for orthopedic MRI	54 patients (22 hip, 32 shoulder)	DL MRI reconstruction showed higher edge sharpness, rSNR and rCNR compared to conventional techniques
Obama *et al*. [212]	2022	Variety of CNNs	MRI for shoulder diseases: effect of C-SENSE and DL reconstruction on examination time and imaging quality compared with that of parallel imaging	30 patients	C-SENSE with and without DL reconstruction showed significantly shorter examination times than PI with and without DL reconstruction; CNR and SNR were higher in examinations with DL reconstruction compared to those without
Hahn *et al*. [213]	2022	CNN (pre-trained)	Image quality and diagnostic performance of accelerated shoulder MRI with DL reconstruction	105 patients, 110 shoulders	Accelerated sequences with DL reconstruction provide 67% scan time reduction with similar subjective image quality, artifacts, and diagnostic performance compared to standard sequences
Kaniewska *et al*. [211]	2023	PROPELLER sequences, Air Recon DL®	DL application to improve image quality and reduce scan time	30 patients	Accelerated PROPELLER sequences with DL post-processing have superior image quality and higher diagnostic confidence compared to conventional PROPELLER sequences

Author	Year	Technique	Clinical application	Patients / dataset	Findings / application
Dratsch *et al*. [214]	2023	Adaptive-CS-Net	Performance of combination of DL and C-SENSE in shoulder MRI	20 patients	Combination of DL and C-SENSE has the potential for further scan time reduction in 2D and 3D MRI of the shoulder
Feuerriegel *et al*. [215]	2023	Adaptive-CS-network	DL reconstruction method for denoising and image enhancement of shoulder MRI in patients with shoulder pain	38 patients	There was improved image quality as well as higher SNR using C-SENSE with DL-based framework
Hahn *et al*. [216]	2023	AIR™ Recon DL	Comparison of PROPELLER with DL reconstruction with conventional reconstruction for shoulder MRI evaluation	92 patients, 100 shoulders	Accelerated PROPELLER with DL reconstruction showed even better image quality than conventional PROPELLER with conventional reconstruction and comparable interrater agreement for shoulder pathologies
Shiraishi *et al*. [217]	2023	DL	Performance of combination of C-SENSE and DL for shoulder MRI with various sequences	37 patients	DL reconstruction method can increase image quality of C-SENSE T1WI, T2WI and FS T2WI of the shoulder, compared to wavelet denoising method
Liu *et al*. [218]	2024	DL reconstruction	Comparison of shoulder MRI scans with and without DL in terms of image quality and scan time	470 patients (70 patients examined with DL-MRI and non-DL-MRI; 400 patients with DL-MRI)	Shoulder DL-MRI can greatly reduce scan time and improve image quality compared to non-DL-MRI
Feuerriegel *et al*. [219]	2024	Adaptive-CS-network	DL-based image quality enhancement of CT-like MRI in patients with suspected trauma	32 patients	Evaluation of traumatic shoulder pathologies is feasible using DL-based algorithm for reconstruction of high-resolution CT-like MRI
Herrmann *et al*. [220]	2024	DL reconstruction	Diagnostic performance and image quality of 5-minute MRI scans with DL protocol for shoulder imaging	30 patients	Novel 5-minute TSE DL-protocol for the shoulder is feasible in daily clinical practice at 1.5 and 3.0 T and reduces 50% of time, while improving image quality and equal diagnostic performance
Xie *et al*. [221]	2024	DL reconstruction	Comparison of standard and DL reconstructed TSE MRI of the shoulder	133 patients	TSE DL reconstruction led to reduced examination time and higher image quality with similar diagnostic confidence compared with standard TSE MRI of the shoulder

2D – 2 dimensional. 3D – 3 dimensional. ARCR – arthroscopic rotator cuff tear. CNN – convoluted neural networks. CNR – contrast-to-noise ratio. C-SENSE – compressed sensing. CT – computed tomography. DL – deep learning. FI – fatty infiltration. FS – fat-saturated. GCN – graph convoluted networks. LHB – long head of the biceps. ML – machine learning. MRA – magnetic resonance arthrography. MRI – magnetic resonance imaging. PD – proton density. PI – parallel imaging. PROPELLER – periodically rotated overlapping parallel lines with enhanced reconstruction. RC – rotator cuff. RCT – rotator cuff tear. SSP -supraspinatus. SLAP – superior labral anterior to posterior. SNR – signal-to-noise ratio. ISP – infraspinatus. SSP – supraspinatus. TSE – turbo spin echo. VRN – value refinement network. WI – weighted imaging.

© 2025 The Author(s). Published by Bentham Science Publisher.

This is an open access article distributed under the terms of the Creative Commons Attribution 4.0 International Public License (CC-BY 4.0), a copy of which is available at: https://creativecommons.org/licenses/by/4.0/legalcode. This license permits unrestricted use, distribution, and reproduction in any medium, provided the original author and source are credited.
